# Semisynthetic Derivatives of Pentacyclic Triterpenes Bearing Heterocyclic Moieties with Therapeutic Potential

**DOI:** 10.3390/molecules27196552

**Published:** 2022-10-03

**Authors:** Gabriela Nistor, Cristina Trandafirescu, Alexandra Prodea, Andreea Milan, Andreea Cristea, Roxana Ghiulai, Roxana Racoviceanu, Alexandra Mioc, Marius Mioc, Viviana Ivan, Codruța Șoica

**Affiliations:** 1 Department of Pharmaceutical Chemistry, Faculty of Pharmacy, “Victor Babes” University of Medicine and Pharmacy Timisoara, Eftimie Murgu Square No. 2, 300041 Timisoara, Romania; 2 Research Centre for Pharmaco-Toxicological Evaluation, “Victor Babes” University of Medicine and Pharmacy, Eftimie Murgu Sq., No. 2, 300041 Timisoara, Romania; 3 Department of Anatomy, Physiology, Pathophysiology, Faculty of Pharmacy, Victor Babes University of Medicine and Pharmacy, 2nd Eftimie Murgu Sq., 300041 Timisoara, Romania; 4 Department of Internal Medicine II, Faculty of Medicine, “Victor Babes” University of Medicine and Pharmacy Timisoara, Eftimie Murgu Square No. 2, 300041 Timisoara, Romania

**Keywords:** pentacyclic triterpenes, lupeol, betulin, betulinic acid, betulonic acid, ursolic acid, oleanolic acid, maslinic acid, corosolic acid, heterocycles, bioconjugates, structure–activity relationship

## Abstract

Medicinal plants have been used by humans since ancient times for the treatment of various diseases and currently represent the main source of a variety of phytocompounds, such as triterpenes. Pentacyclic triterpenes have been subjected to numerous studies that have revealed various biological activities, such as anticancer, antidiabetic, anti-inflammatory, antimicrobial, and hepatoprotective effects, which can be employed in therapy. However, due to their high lipophilicity, which is considered to exert a significant influence on their bioavailability, their current use is limited. A frequent approach employed to overcome this obstacle is the chemical derivatization of the core structure with different types of moieties including heterocycles, which are considered key elements in medicinal chemistry. The present review aims to summarize the literature published in the last 10 years regarding the derivatives of pentacyclic triterpenes bearing heterocyclic moieties and focuses on the biologically active derivatives as well as their structure–activity relationships. Predominantly, the targeted positions for the derivatization of the triterpene skeleton are C-3 (hydroxyl/oxo group), C-28 (hydroxyl/carboxyl group), and C-30 (allylic group) or the extension of the main scaffold by fusing various heterocycles with the A-ring of the phytocompound. In addition, numerous derivatives also contain linker moieties that connect the triterpenic scaffold with heterocycles; one such linker, the triazole moiety, stands out as a key pharmacophore for its biological effect. All these studies support the hypothesis that triterpenoid conjugates with heterocyclic moieties may represent promising candidates for future clinical trials.

## 1. Introduction

Since ancient times, humans have used herbs to cure various diseases, and even though they were initially instinctively chosen, people began to know precisely which plants to use for a particular disease due to the experience gained over time [1]. Nowadays, these plants are known as medicinal plants and represent the main source for numerous active phytocompounds with beneficial properties for human health [2], as numerous drugs available, such as morphine, digitoxin, paclitaxel, artemisinin, and tiotropium, are either phytocompounds or their semisynthetic derivatives [3]. 

Pentacyclic triterpenes are a well-represented class of phytocompounds resulting from plants’ secondary metabolism, mainly through squalene cyclization [4]; they can be classified based on their structure, the vast majority being lupane, oleanane, or ursane derivatives [5]. Various studies have revealed that these phytocompounds are associated with a wide range of biological activities such as anticancer, anti-inflammatory, hepatoprotective, cardioprotective, and antidiabetic effects, among many others [6]. Unfortunately, their use in therapy is currently hampered by their high lipophilicity, which is considered to greatly affect their in vivo bioavailability [7]. In order to overcome this obstacle, several strategies have been employed to increase these compounds’ hydrophilicity, including chemical derivatization [8], cyclodextrin complexation [9], and liposomal nanoformulations [10]. Despite many such approaches, the scientific community has not yet been able to introduce a compound of this class or its derivative into therapy; bevirimat is the only triterpene derivative that has reached phase 2 clinical trials for its anti-HIV properties, but in 2010, these studies had to be discontinued due to the acquisition of drug resistance [11]. Without a doubt, the most common approach employed to enhance the therapeutic properties of these phytocompounds remains chemical derivatization, allowed by their complex core structure. Due to their versatility and high compatibility with various structures/biological targets, heterocycles have always been key elements in medicinal chemistry, being found in the structure of numerous drugs, drug candidates, and biologically active molecules [12].

The current work aims to summarize the most recent published studies on the topic of semisynthetic derivatives of pentacyclic triterpenes bearing heterocyclic moieties and focuses on biologically active derivatives and their structure–activity relationships (SAR). The studies cited were collected from two major databases recognized by the scientific community, PubMed (http://www.ncbi.nlm.nih.gov/pubmed, accessed on 13 May 2022) and Web of Science (WOS, www.webofknowledge.com, accessed on 13 May 2022). The databases were searched using combinations of the key terms “pentacyclic triterpenes” and “heterocycle”, covering the last 10 years. The inclusion criteria for the current manuscript included: (1) original articles; (2) abstracts or full text available; (3) articles containing data about pentacyclic triterpene heterocyclic derivatives and bioconjugates. The exclusion criteria included: (1) duplicates; (2) articles in languages other than English; (3) review-type papers; (4) irrelevant articles for the topic; (5) articles that discussed only the synthesis of the derivatives without their biological assessment; (6) articles that discussed ring-modified derivatives. Regarding the studies that focused on obtaining not only heterocyclic derivatives but also aryl-derivatives, the review also presents these aryl-derivatives due to the fact that they exhibited significant biological activities in their respective studies, even if they could not be classified as heterocyclic derivatives or bioconjugates. The database screening resulted in a total of 143 articles; however, after the application of the above-mentioned inclusion and exclusion criteria, only 90 articles were subjected to further analysis.

## 2. Lupeol Derivatives

**Figure 1:**
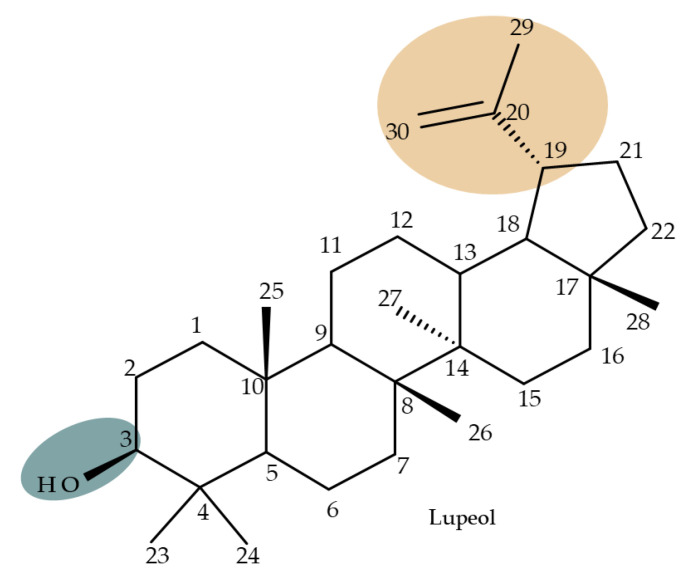
Frequent targets for the derivatization of lupeol.

Lupeol (lup-20(29)-en-3β-ol; Figure 1) is a lupane-type pentacyclic triterpene found in various medicinal plants that is associated with a variety of therapeutic activities, including anticancer, anti-inflammatory, and antiparasitic effects [13]. Several semisynthetic derivatives have been obtained to improve its bioavailability and therapeutic properties, mainly through the derivatization of the C3-OH and C19-isopropenyl positions (Figure 1) [14]. 

Khan et al. obtained a series of C19-isopropylene-modified lupeol derivatives and further assessed their influence on in vitro glucose absorption. The results obtained for several heterocyclic derivatives either containing a pyrazoline moiety (Lup 1–3; Table 1) or obtained through C19-isopropylene cyclization (Lup 4–7) showed that the introduction of heterocyclic moieties to the lupeol scaffold induces an inferior effect on the L6 skeletal muscle cells’ glucose absorption compared with acyl-derivatives tested under the same environment [15]. Complementary to these findings, the same research group synthesized several phenyl and pyrazoline lupeol derivatives through C3 and C19 chemical modulation (Lup 8–11) that showed strong inhibitory effects against protein tyrosine phosphatase-1B (PTP-1B), a novel target protein for the development of type 2 diabetes medication. Following SAR analysis, the following aspects emerged: (1) the insertion of a carbonyl or pyrazoline moiety into the C-19-isopropenyl group led to activity loss; (2) both the phenyl ring and the substituted aliphatic ring increased the compound’s PTP-1B inhibitory activity [16]. Moreover, the inhibitory effect exerted against PTP-1B provides valuable information regarding the possible mechanism of action exerted in diabetes that seems to be linked to the inhibition of the insulin signal transduction pathway, which is responsible for increasing the sensitivity of membrane insulin receptors [17] and, subsequently, reducing the associated insulin resistance. Collectively, these studies show that lupeol derivatization at C3-OH and C-19-isopropylene positions could be further used to obtain promising new antidiabetic drugs that can reduce insulin resistance in diabetic patients.

A series of lupeol derivatives was synthesized by Castro et al. through C3, C16, or C-19-isopropylene chemical modulation, followed by their cytotoxicity assessment against two prostate cancer cell lines, LNCaP and PC-3. Among the tested derivatives, two compounds bearing a double esterification at the C3- and C-16 hydroxyl groups with benzoyl chloride (Lup 12) or p-bromo-benzoyl chloride (Lup 13), respectively, exerted lower cytotoxicity compared with the lead compound IC_50_ > 70 µM, a Lupeol–C16-sulfate derivative, as well as with doxorubicin used as a standard drug [18]. The authors concluded that grafting a sulfate group onto a sterol core structure is beneficial for the development of prostate cancer treatments, a hypothesis supported by similar in vitro studies [19,20]; however, further studies are needed to explain in finer detail the superior effect of the sulfate derivative compared to the non-sulfate-heterocyclic derivatives.

## 3. Betulin

**Figure 2:**
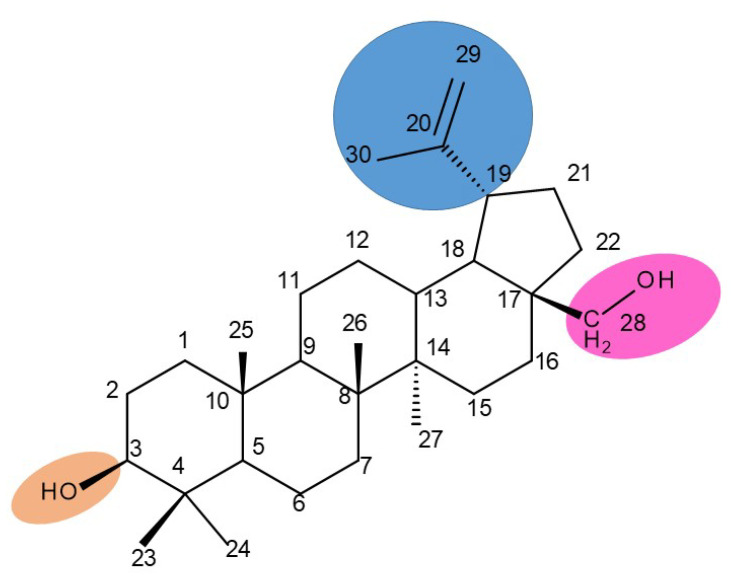
Frequent targets for the derivatization of betulin.

Betulin (lup-20(29)-ene-3β,28-diol, betulinol) is a lupane-type pentacyclic triterpenoid bearing two C3, C28-hydroxyle, and one isopropylene moiety at C19 [21]; this phytocompound can be found in various plant species, in particular, members of the Betulaceae family (i.e., *Betula alba*, *B. pubescens*, *B. pendula)* [22]. Betulin has been shown to exhibit a wide spectrum of pharmacological activities, including not only its well-known anticancer, antiviral, and antimicrobial effects but also newly explored antidiabetic, anti-inflammatory, antidyslipidemic, cardioprotective, anxiolytic, antidepressant, and neuroprotective activities [23]. In order to address the issue of its poor pharmacokinetics, low water solubility, and poor bioavailability, most structure–activity relationship (SAR) studies have focused on obtaining more hydrophilic derivatives by modifying the C-28 (hydroxyl moiety), C3 (hydroxyl moiety) or the C20–29 double bond (alkene moiety) groups (Figure 2) [24]. 

### 3.1. C3 Modifications

Saponins represent a heterogeneous class of triterpenoids or steroid glycosides that are widely distributed in nature and exhibit various biological activities, including not only antiproliferative effects against many types of cancers but also antiviral, antifungal, and anti-inflammatory activities [25]. However, since saponins containing a betulin scaffold rarely occur naturally, their synthesis has become an issue of interest for many researchers in this field [26]. It was reported that betulin derivatives, obtained through the attachment of modified sugar fragments both at the C-28 and C-3 positions, exhibited strong cytotoxic properties against G361 (malignant melanoma), MCF7 (breast adenocarcinoma), and HeLa (cervical carcinoma) cell lines; the derivatives were synthesized through the glycosylation of the previously acetylated C-3 and C-28 hydroxyl groups with arabinopyranosides, whose 3,4-diol groups remained protected. The most cytotoxically active compound was Bet 1 (Table 2), obtained by attaching an L-arabinopyranosyl group bearing two methoxy groups at C-3, while the C-28 hydroxyl group remained unmodified with an IC50 = 1.5 µM [27].

### 3.2. C28 Modifications

Tomanek et al. tested the biological benefits of 1,4-quinone-conjugated betulin against seven cancer cell lines. Data showed that the quinone moiety attached to betulin at C-28 through a triazole linker was essential for exhibiting anticancer activity; such compounds exerted the strongest anticancer activity against lung cancer (A549 cell line). SAR analysis revealed that the position of the nitrogen atom plays a significant role in terms of future biological effects; thus, compounds (Bet 2–5) that contain a 5,8-isoquinolinedione group exhibited higher activity than those containing a 5,8-quinolinedione moiety. Furthermore, the particular structure of the 1,4-quinone scaffold strongly influences the potency of the biological activity in the following sequence: 2-methyl-5,8-quinolinedione > 1,4-naphtoquinone > 5,8-isoquinolinedione > 5,8-quinolinedione [28]. 

The triazole linker was also used by Anh et al., who synthesized a series of eleven compounds through click chemistry by coupling C28-betulin-triazoles with the antiviral 3′-azido-3′deoxythydimine (AZT); the compounds were further tested in vitro against KB (human epithelial carcinoma) and HepG2 (liver carcinoma) cancer cell lines. The results showed that all compounds exerted at least moderate cytotoxic activity against both cancer cell lines; in particular, the hybrid Bet 6 (Table 2), containing one C28-fumaryl moiety, showed the highest anticancer activity, with IC50 values of 0.3 μM against KB and 1.3 μM against HepG2 cancer cells. Consequently, one may state that the addition of AZT to 28-O-acylated betulin may lead to highly effective anticancer hybrid compounds [29]. 

The assessment of betulin bioconjugates in terms of their effects on the mitochondrial membrane potential and, subsequently, on the oxidative phosphorylation was conducted by Dubinin et al., who synthesized a novel betulin conjugate by attaching the (E)-4-(1h Indol-3-yl-vinyl)pyridine fragment to the C-28 bromoalkyl betulin ester (Bet 7) and evaluated its cytotoxic activity in isolated rat mitochondria. The authors previously demonstrated that betulin can solely modify the mitochondrial membrane; in addition, the newly developed betulin conjugate exhibited a strong cytotoxic effect by selectively accumulating in mitochondria, thus leading to the suppression of respiration and oxidative phosphorylation as well as ROS overexpression, finally resulting in cell death [30]. 

Betulin imidazole derivatives have been tested against leishmaniasis, a highly difficult-to-treat tropical disease; Sousa et al. obtained a series of nine betulin derivatives that were further assessed in terms of pharmacological interaction with miltefosine, an antileishmanial drug currently used in polytherapy. According to SAR analysis, the insertion of imidazole moieties at the C-28 position through an ester bond induced highly promising inhibitory effects against *Leishmania infantum* growth; by contrast, the addition of carbamate moieties in the same position provided lower inhibitory effects compared with the parent compound. The most active compound against the promastigote forms of *L. infantum* was 3-hydroxy-20-lupan-29-oxo-yl-1H-imidazole-1-carboxylate (Bet 8), having its C-20-29 double bond oxidized into an aldehyde and displaying an imidazole scaffold grafted at the C-28 position through an ester bond, which revealed an IC50 value of 50.8 μM; furthermore, when administered in combination with miltefosine, the same betulin derivative exerted a synergistic effect, with the IC50 value decreasing to 6 μM. Most importantly, the compound did not cause any significant macrophage necrosis, thus suggesting little to no toxicity to the host cell [31]. 

The insertion of an isoxazole moiety at C-28 through the oxidation of the hydroxyl group followed by condensation and cycloaddition led to a series of compounds that were further tested against MCF7 breast cancer, U-87 MG multiforme glioblastoma, A549 lung carcinoma, and HepG2 hepatocarcinoma cell lines, as well as in hTERT human fibroblasts. The SAR study proved that the substituents attached to the isoxazole ring significantly influenced the overall cytotoxic effects; as an example, the saponin-like derivatives containing glucose substituents grafted on the isoxazole fragment induced cytotoxic effects against A549 and MCF7 cell lines, but no activity was recorded in the HepG2 cells. The most promising results were reported for derivatives bearing small hydrophilic substituents on the isoxazole ring, such as hydroxymethyl (Bet 9–10), 2-hydroxypropan-2-yl (Bet 11), (2-hydroxyethoxy)methyl (Bet 12), carboxyl (Bet 13), and (acetylthio)methyl (Bet-14), for which IC_50_ values within the micromolar range were calculated; moreover, the selected compounds proved to be harmless against healthy human fibroblasts [32]. 

Betulin and artesunic acid were chosen for the development of new molecules that could effectively treat the drug-resistant *P. falciparum* malaria parasite. A series of hybrid derivatives were synthesized and analyzed in vitro against malaria parasites as well as human cytomegalovirus (HCMV). The hybrids were obtained with several esterification reactions, either between individual molecules of betulin and artesunic acid or by additionally using ferrocene moieties as linkers. The most active compound was Bet 15, obtained using C28-betulin esterification with one molecule of artesunic acid, which exhibited a 20-fold activity increase compared to botulin, as indicated by the EC_50_ value of 0.24 μM. However, compared with the effect of chloroquine against *P. falciparum*, none of the derivatives presented significantly improved antimalarial activity [33]. 

### 3.3. C30 (Allylic) Modifications

Pyridinium–betulin derivatives were revealed to alter the properties of the mitochondrial membrane [34]; consequently, the authors synthesized three 4-methyl- and 3,5-methyl-pyridinium betulin derivatives by attaching the substituted pyridinium fragments to the C-30 position and further tested the resulting compounds on artificial membrane systems (liposomes), prokaryotic organisms (*E. coli)*, and eukaryotic cells (rat thymocytes), as well as on rat liver mitochondria. The study showed that all three compounds inhibited the growth of *E. coli* without exerting a cytotoxic effect on rat thymocytes. However, the presence of the methyl substituents on the pyridine ring was essential for their activity in mitochondria, with a SAR assessment proving that 30-pyridinium-1-yl-lup-20(29)-ene-3,28-diol bromide, which lacks methyl groups, was not able to induce mitochondrial swelling and aggregation; hence, it did not cause membrane permeabilization. Moreover, Bet 16 (Table 2), containing two methyl groups at the p-position and m-position of the pyridine ring, was able to permeabilize the mitochondrial membrane; the compound presumably caused lipid pore formation, therefore increasing the proton permeability of the lipid phase mitochondrial membrane, hence causing organelle dysfunction. Moreover, Bet 16 could exert a protonophore uncoupling effect that was not shown by the other two compounds due to their lack of lipophilicity [35]. 

Similarly, three derivatives were synthesized using pyridinium fragments; the conjugates were obtained by attaching the pyridinium fragment to the C-30 allylic bond of betulin, betulonic acid, and betulin 28-O-acetate, leading to the increased polarity of the molecule and creating new amphiphilic betulin conjugates. The antibacterial and antifungal properties of the three derivatives and their triterpenic analogs were tested against both Gram-positive *Staphylococcus aureus* and Gram-negative *Pseudomonas aeruginosa*, as well as on *Candida albicans* and *Cryptococcus neoformans* fungi. The antimicrobial effect of the initial triterpenic compounds was insignificant compared to their pyridinium derivatives, showing that the addition of pyridinium moieties led to an increase in the biological activity and proving the importance of pyridinium fragments for the antimicrobial effect. The most promising antimicrobial results were reported for compound Bet 17, obtained by attaching a pyridinium salt to C-30 on the betulin skeleton, which exhibited strong inhibitory activity against both fungi (minimum inhibitory concentration of 8 μg/mL) and *S. aureus* (minimum inhibitory concentration of 1 μg/mL) [36]

### 3.4. C3, C28, C30 Modifications

The previously mentioned C28 modulation by using 1,4-quinone moieties can also be conducted in other positions on the betulin scaffold; a large series of betulin-1,4-quinone hybrids were synthesized by conjugating betulin with various compounds bearing the 1,4-quinone fragment through the use of a linker. Different 1,4-quinone fragments were attached to C-3, C-28, and C-30 positions, respectively, of either betulin or betulin derivatives, such as 3-acetylbetulin, 28-acetylbetulin, 28-propionylbetulin, and 30-hydroxy-3,28-diacetylbetulin. All derivatives were tested against a panel of human cancer cell lines, revealing that, in several compounds, the presence of the betulinyloxy group connected to the 1,4-quinone fragment increases their anticancer activity (Bet 18–22; Table 2). Further SAR analysis showed that the efficacy of the antitumor effects strongly depends on the tested cell line but mostly on the type of the 1,4-quinone moiety. As such, a higher activity against amelanotic melanoma cells compared with melanotic ones was recorded for the majority of the hybrids, while the potency of the 1,4-quinone moiety varied in the following order: 1,4-naphtoquinone > 2-methyl-5,8-quinolinedione > 5,8-quinolindione. Furthermore, the position of the substituents in the 1,4-quinone groups is essential; i.e., the introduction of a methyl group at the C2 position of the 5,8-quinolindione moiety causes important changes in terms of anticancer activity against breast malignancies. In addition, the insertion position of the 1,4-quinone group on the triterpene scaffold may alter the overall cytotoxic effect. Testing the obtained hybrids against several cancer cell lines with different levels of the NQO1 protein (A549-lung, MCF7-breast, C32-melanoma), the authors reported the highest activity against lung cancer for most hybrids. The anticancer effects were correlated with a molecular docking study in order to assess a potential interaction between the compounds and the human NADPH-quinone oxidoreductase (NQO1) protein site; it was noticed that the biological activity of triterpenes increased in cell lines with higher levels of the NQO1 protein, such as melanoma (C-32) and breast (MCF-7) and lung (A-549) cancer. The docking data confirmed that the biological effects of the studied compounds are the result of an interaction between the 1,4-quinone fragment and the active site of the enzyme [37].

The neuroprotective activity of several triterpenoid derivatives containing various sugar moieties was tested in vitro on neuron-like SH-SY5Y neuroblastoma cells; the compounds were prepared by attaching triazole-modified sugar moieties through ester bonds to the C30 position in the molecule of C3, C28-diacetylated betulin. The most efficient neuroprotective compound proved to be one betulin conjugate containing a tetraacetyl-β-glucose moiety (Bet 23; Table 2); furthermore, the elimination of the sugar fragment resulted in a free triazole betulin derivate (Bet 24), both compounds being able to modulate oxidative stress and exert neuroprotective effects in glutamate-induced neurodegeneration models. The results also revealed that even though compound Bet 24, a triazole-substituted acetylated-betulin, exhibited the most effective neuroprotective effect in the lowest concentration (1 μM), it also proved very cytotoxic in higher concentrations (10 μM). The evaluation of the two betulin derivatives on caspase-3 and -7 activity revealed only moderate effects, thus indicating that the underlying mechanism of neuroprotection needs further investigation; however, the two semisynthetic derivatives could serve as lead structures for future studies regarding the impact of triazole substitution [38]. Betulin glycoconjugates obtained as a result of attaching N-acetyl-D-galactosamine fragments to the C-3 or/and C-28 positions in betulin’s molecule also using triazole as a linker were tested in vitro against HepG2 and Huh7 hepatocellular carcinoma cells, PC3 prostate cancer cells, and A549 lung adenocarcinoma cells. The bivalent derivatives, in particular, compound Bet 25, bearing two modified galactosamine fragments at C-3 and C-28, respectively, exerted stronger cytostatic effects against HepG2 cells compared with monovalent glycoconjugates by inducing G0/G1 cell cycle arrest [39]. In opposition to these findings, Grymel et al., who synthesized a series of betulin glycoconjugates by attaching D-glucose or D-galactose through triazole linkers to the C-3 and/or C-28 positions, reported that the glycoconjugates containing only one sugar unit exhibited equal or lower biological activity compared with the parent compound. Moreover, when tested in HCT116 cancer cells, the disubstituted glycoconjugates exerted a stimulatory effect on the growth of cancer cells. The authors formulated the hypothesis that the sugar moieties may be degraded by hydrolytic enzymes, thus providing the cells with an extra source of energy. In support of this hypothesis, compound Bet 26, containing only the free 1,2,3-triazole moiety (without the sugar fragment), exhibited impressive cytotoxic activity, but also proved to be extremely toxic against normal human dermal fibroblast–neonatal cells; these findings further confirmed the key role played by the triazole moiety for the occurrence of cytotoxic activity [40]. In fact, the relevance of substituting betulin with triazole heterocycles was also emphasized by Bebenek et al., who synthesized triazole derivatives by adding organic azides to alkyl betulin derivatives; the cytotoxicity of the compounds was tested against T47D human ductal carcinoma, MCF-7 human adenocarcinoma, SNB-19 glioblastoma, Colo-829 human malignant melanoma, and C-32 human amelanotic melanoma cell lines. Furthermore, their antibacterial activity was evaluated against Gram+ and Gram– bacteria. The bioconjugates contained either one triazole moiety at the C-28 position or two triazole moieties at both C-3 and C-28; in addition, various aliphatic or aromatic substituents were inserted in the triazole fragments. The most promising compound in terms of biological activity was compound Bet 27, containing two 3-hydroxypropyl-triazole rings at C-3 and C-28, respectively, which exhibited cytotoxic effects against all tested cancer cell lines (IC_50_ ranging between 6.5–68.3 µM). Furthermore, compound Bet 28, containing a 30-deoxythymidine-5′-yl moiety fragment at C-28, was the only one with antibacterial activity against the two Gram- bacterial strains, *K. pneumoniae* and *E. coli* [41].

### 3.5. C-3 and C28 Modifications

The simultaneous modification of both the C-3 and C-28 positions in the betulin molecule may lead to the synthesis of novel mitochondria-targeted derivatives; such derivatives were obtained by attaching triphenylphosphonium moieties to the C-3 and/or C-28 acylated hydroxylic groups, resulting in nineteen betulin conjugates, which have been tested against four cancer cell lines, including human lung carcinoma (A549), human primary glioblastoma (U87), human cervical carcinoma (HeLa), and human colorectal carcinoma (HCT116), as well as one normal colon epithelial cell line (NCM460). After biological assessment, the most promising compound, Bet 29 (Table 2), which was obtained through the conjugation of the previously acetylated betulin derivative (3-O-(3′-acetylphenylacetate)-betulin) with one triphenylphosphonium moiety at the C-28 position, revealed a strong inhibitory effect against HCT116 cancer cells (IC_50_ = 0.66 μM) by inducing cycle arrest in the G2/M phase. Furthermore, a structure–activity relationship study on the series of the 19 derivatives suggested that the preferential integration of the triphenylphosphonium moiety into the C28 position might optimize the overall cytotoxic effect [42]. 

The impact of grafting triphenylphosphonium moieties to the betulin scaffold was also investigated by Tsepaeva et al.; in a similar manner to the previous study, the triphenylphosphonium moiety was inserted into both the C-28 and C-3 positions, or into the C-28 position alone, through an acyl linker. The anticancer effects of the resulting compounds were assessed against several cancer cell lines: human breast cancer (MCF-7), prostate adenocarcinoma (PC-3), and vinblastine-resistant human breast cancer (MCF-7/Vinb), as well as on human skin fibroblast (HSF). Although most disubstituted compounds exhibited nanomolar range IC50 values, the most active derivative, Bet 30, possessing only one triphenylphosphonium moiety grafted at C-28, exerted superior cytotoxic activity against all tested cancer cell lines, but particularly against MCF-7/Vinb, with an IC_50_ of 0.045 μM. A SAR evaluation revealed that the triphenylphosphonium-substituted compounds induced stronger biological activities than both the parent compound and its acylated derivatives. Furthermore, the length of the alkyl chain linked to the ester bond significantly influenced the cytotoxic effect; as such, the methylene group induced lower biological activity compared with propylene and butylene fragments [43]. 

Considering the key role heterocycles play as pharmacophores in medicinal chemistry, Csuk et al. synthesized 24 betulin-derived propargylamines by first subjecting betulin to a series of transformations in order to obtain 28-propargyl betulins, which were then further conjugated with different types of N-bearing moieties through Mannich reactions using alkynes, aldehydes, and amines. The C-3 hydroxyl function was altered in some derivatives; hence, several hybrids were betulone derivatives as a result of oxidation, while others had their hydroxyl group previously acetylated. The isopropenyl moiety of the lupane skeleton remained unaffected for all derivatives. The betulin conjugates were further analyzed in terms of anticancer activity against nine human cancer cell lines, allowing for the formulation of some conclusions regarding their structure–activity relationships. A SAR analysis emphasized the importance of the C-28 alkynic function in the biological effect reported for the synthesized compounds. Furthermore, the introduction of N-heterocyclic substituents and amino moieties on the triterpenic scaffold led to significantly improved cytotoxic effects, while 3-O-acetyl hybrids exhibited higher biological activities compared to their deacetylated parent analogs [44]. 

In order to evaluate the mechanism of mitochondrial toxicity, a series of dimethylaminopyridine derivatives with triterpenoid scaffolds were synthesized by Bernardo et al. by attaching one, two, or three pyridinium moieties to C-3, C-28, or C-30, respectively, in the molecule of betulin (four compounds) or betulinic acid (one compound); the five derivatives were selected from a larger series of previously synthesized compounds [45] due to showing the highest cytotoxic activity against melanoma cells. Among the five compounds, Bet 31, a dimethylaminopyridinium acetoxyl betulin derivative containing two dimethylaminopyridinium moieties at C-3 and C-28, respectively, had already been emphasized as the strongest mitochondrial disruptor in melanoma cells; overall, the number, position, and orientation of the dimethylaminopyridinium groups were directly correlated with the effectiveness of mitochondrial disruption [34]. The authors decided to further decipher the underlying mechanism of action by conducting tests on other tumor cell lines, MCF-7 and Hs 5787 breast cancer, as well as on BJ normal fibroblasts and isolated hepatic mitochondria; they revealed that all derivatives displayed a planar geometry, which provides affinity for the fatty acids in the structure of membrane phospholipids. Furthermore, the polar groups in their structure become protonated in the physiological environment and naturally migrate toward the negatively charged mitochondrial matrix. The authors were also able to confirm a previously formulated hypothesis concerning the high relevance of the position occupied by the dimethylaminopyridinium moiety in the triterpene molecule. Compounds Bet 32 and Bet 33 exhibit highly similar structures, with the dimethylaminopyridinium moiety migrating from position C28 in Bet 32 to position C3 in Bet 33; however, their biological effects are significantly different. Thus, although both compounds are potent mitochondrial disruptors, only Bet 32 interferes with the mitochondrial phosphorylative system. The authors concluded that effective triterpene derivatives must show an amphiphilic nature and have the potential of being positively charged in order to interact with the negative mitochondria potential [34]. 

Another approach for the synthesis of novel betulin derivatives consisted of the introduction of 2-amino-2-deoxy-D-glucopyranosyl and -D-galactopyranosyl moieties, respectively, to the C30 position through a glycosidic bond. The resulting compounds, 28-acetylbetulin-3-yl-2-amino-2-deoxy-D-glucopyranoside and betulin-3-yl-2-amino-2-deoxy-D-gluco- and D-galactopyranoside (Bet 34–36), were further tested against the MCF-7 breast cancer cell line and HDFa human dermal fibroblasts, as well as against human pathogenic bacteria and fungi. The three compounds exhibited highly similar structures, the main differences consisting of the orientation of the hydroxyl groups in the sugar fragment and the presence/absence of C28-acetyl esterification. The experimental data indicated that all three saponins induced cytotoxic activities in both normal and cancer cells; however, betulin-3-yl-D-galactosamine (Bet 36) was the most active compound against MCF-7 cells (IC_50 =_ 1.74µM) while moderately acting against normal cells, thus exhibiting some degree of selectivity. In terms of antimicrobial properties, all three betulin-3-yl glycosaminosides have poor-to-no activity against fungi as well as Gram-positive and Gram-negative bacteria [46]. 

A study of betulin bidesmosides was also conducted by Korda et al.; the bioconjugates were obtained by attaching L-rhamnose, D-idose, and L-arabinose, respectively, directly to the betulin aglycone in both the C-3 and C-28 positions. The compounds were tested in vitro on CEM T-lymphoblastic leukemia, MCF7 breast adenocarcinoma, HeLa-cervical carcinoma, and G-361 malignant melanoma cell lines, as well as on normal BJ skin fibroblasts. The SAR study revealed that the presence of the L-rhamnose moiety is essential for the cytotoxic effect of betulin bioconjugates, with its presence in either C-3 or C-28 positions inducing strong biological effects. Contrarily, the presence of L-arabinose as a substituent at C-3 decreases the biological activity of the compound, whereas its presence at C-28 has no influence over the cytotoxic effect. The most active compounds against all cancer cell lines were Bet 37 and Bet 38, bearing L-rhamnose fragments at C-28 and C-3, respectively, with IC_50_ values below 2.5 μM for all cell lines [47]. Similar betulin bidesmosides were previously synthesized by Mihoub et al., who selected 28-O-L-rhamnopyranosylbetulin 3β-O-L-rhamnopyranoside (Bet 39) as the most active compound. The derivative, bearing two rhamnopyranosyl moieties at C3 and C28, was tested in vitro against six lung cancer cell lines (A549, NCI-H2087, NCI-H522, NCI-H1993, NCIH1755, and LLC1) and in vivo on tumor-bearing mice; it proved very active against all tested cancer cell lines, having IC_50_ values ranging between 2.9 and 5.9 μM. However, the compound did not show selective cytotoxicity against cancer cells and acted against the healthy cell lines MRC-5 and HEL299 as well, thus suggesting the possibility of some side effects in vivo. Contrary to expectations, when tested in vivo on healthy mice, no sign of toxicity was reported; moreover, Bet 39 was able to significantly inhibit tumor growth in tumor-bearing mice, presumably by enhancing cell apoptosis, as indicated by its reported in vitro ability to induce G2/M phase cell cycle arrest [48]. The presence of one L-rhamnose moiety in the molecule of betulin derivatives even has the ability to counteract the negative influence of other groups in terms of cytotoxic effects; the attachment of an ichopanol core to the triterpenoid skeleton induces a loss of cytotoxic activity, with the exception of compounds bearing rhamnose moieties, as shown by Sidoryk et al. The authors synthesized a series of betulin and betulinic acid saponin conjugates by attaching D-mannose, D-idose, D-arabinose, and L-rhamnose units to the C-28 position of the betulin skeleton; some of the derivatives were also acetylated at the C-3 position. The compounds were tested in vitro on CEM T-lymphoblastic leukemia, MCF-7 breast adenocarcinoma, and HeLa cervical carcinoma cell lines; the results showed that compound Bet 40, containing one L-rhamnoside moiety, exhibited moderate cytotoxicity and displayed IC_50_ values within the 23.6–34.4 μM range. The highest cytotoxicity was exerted by betulinal, which was active against CEM and HeLa cell lines, but, at the same time, it proved to be the most cytotoxic in normal cells as well. Following SAR evaluation, the authors concluded that the acetylation of the C-3 hydroxyl increased the biological activity of the bioconjugates compared with the parent compound [49]. 

### 3.6. A-Ring-Fused Heterocyclic Derivatives

Numerous derivatives were obtained through modifications of ring A; Grishko et al. synthesized a novel series of A-ring-fused heterocyclic betulone derivatives, which were further tested against five cancer cell lines (A549, HCT 116, Hep-2, MS, and RD TE32). According to a SAR study, the C(2)-C(3) fusion with oxazole, isoxazole, triazole, and triazine heterocycles, combined with a 28-hydroxyl moiety, induced far superior cytotoxic activities compared with their 28-acylated azole precursors. Moreover, the oxidation of the 28-hydroxyle moiety in the triazole-fused derivative led to significant suppression of the biological activity, while the presence of a 28-carboxyl fragment combined with a triazine moiety attached to the triterpenoid skeleton highly improved the cytotoxic effect. The study identified N-acetyltriazole betulin as a lead compound (Bet 41) due to its strong antiproliferative effect against all tested cancer cell lines, but particularly against HCT 116 (IC_50_ = 5.67 µM) cells, which showed the highest sensitivity to the newly synthesized compounds [50].

The anti-inflammatory properties of ring A-fused/C-D-fused heterocyclic betulin conjugates were tested by Laavola et al. by measuring the expression of nitric oxide synthase (iNOS) and other inflammation markers. According to a SAR, the conjugates obtained by fusing a heterocyclic group to ring A exerted the highest inhibitory activity, especially when pyridine or pyrazine moieties were used. Moreover, the derivatization of the C-28 hydroxymethyl moiety into an oxime group significantly improved the compound’s biological activity; by contrast, the indole derivatives demonstrated weaker activity compared with the parent compound. The study revealed that compound Bet 42 (pyrazole A-fused betulin derivative), having an oxidized C-28 hydroxyl function, exhibited the highest in vitro inhibitory effect on carrageenan-induced paw inflammation in mice, as indicated by the reduction in nitric oxide production as well as the expression of pro-inflammatory cytokines and prostaglandin synthase-2 (COX-2) [51].

## 4. Betulinic Acid

**Figure 3:**
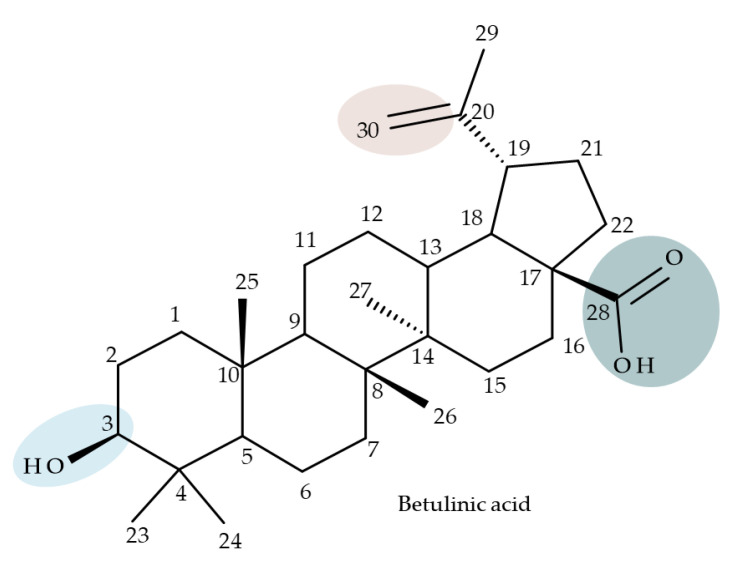
Frequent targets for the derivatization of betulinic acid.

Betulinic acid (BA; 3β-hydroxy-lup-20 (29)-en-28-oic acid; Figure 3) is a widespread lupane-type pentacyclic triterpene that has been the subject of numerous studies highlighting its biological activities in various pathologies such as diabetes, microbial and viral infections, inflammatory diseases, and numerous types of cancer, among others [52]. In order to improve its bioavailability and enhance its therapeutic effect, a large number of chemical modulations have been conducted, mostly in the C-3, C-28, and C-30 positions (Figure 3) [53], but also ring modifications in the BA main scaffold [54].

### 4.1. C2 Modifications

Wang et al. synthesized a BA-nucleoside bioconjugate (BA 1) through a click chemistry reaction between the C2-propargyl derivative of BA and 4′-azido-2′-deoxy-2′-fluoro-β-D-arabinocytidine—which exerted potent anti-HIV activity in vitro compared with the parent compounds—with an IC_50_ = 7.80 nM [55]. Furthermore, in a complementary study developed by the same research group, a stronger hepatoprotective effect of BA 1 compared with BA was found in vivo in an alcoholic liver disease mouse model. The derivative exerted a potent antioxidant effect in hepatocytes that was mainly attributed to the presence of the nucleosidic group, which subsequently increased the molecule’s hydrophilicity [56]. Altogether, these positive results obtained both in vitro and in vivo show that BA 1 should be further investigated for its potential as a hepatoprotective drug candidate.

### 4.2. C3 Modifications

Several nitrogen-containing heterocycles were conjugated with BA by reacting the triterpenic acid and C3-amino-BA with various acids bearing pyrrolidine, piperidine, pyrazine, and pyridine moieties; all compounds were tested in terms of cytotoxicity against a panel of eight non-drug-resistant (human colon carcinoma: HCT-116 and HT-29; human prostate cancer: PC-3 and DU-145; human breast cancer: MDA-MB-231, MCF-7, and T47D; murine breast cancer: 4T1), one multidrug-resistant (MCF-7/ADR), and one normal (HAF; human fibroblasts) cell lines. The tests revealed two amide derivatives, compounds BA 2 and BA 3, each containing one piperidine moiety, which have shown high selective cytotoxicity against the above-mentioned cancer cell lines with average IC_50_ values of 1.62 µM and 1.19 µM, respectively. The SAR evaluation revealed that the introduction of aromatic N-heterocycles (pyrazine and pyridine) is unfavorable for the cytotoxic effect of the resulting derivative. In contrast, the introduction of saturated N-heterocycles (pyrrolidine and piperidine) significantly increases cytotoxicity, particularly against the multidrug-resistant cell line MCF-7/ADR [57]. Complementary to these findings, BA derivatives obtained by either C3 or C28 esterification with different heteroaromatic amides containing a pyridine heterocycle showed strong cytotoxicity in vitro against G-361 (melanoma), MCF7 (breast cancer), HeLa (cervical cancer), and CEM (leukemia) cell lines with IC_50_ values ranging from 0.5 to 2.4 μM. A SAR evaluation also revealed that, in the series of C3-picolyl-BA derivatives (BA 4–6), the o- and m-substitution of the pyridine heterocycle favors cytotoxic activity, while in the series of C28-picolyl derivatives (BA 7–9), the p- and m-substitutions increase their cytotoxicity as opposed to the o-substitution [58]. Collectively, these findings suggest that the use of aromatic or saturated N-heterocycles for the derivatization of BA could lead to various levels of cytotoxicity against different types of cell lines. An evaluation using an extended panel of cell lines could provide a better overview of the cytotoxic effects of the newly synthesized derivatives; however, the application of this method is currently limited for most research groups due to the costs involved.

**Figure 4:**
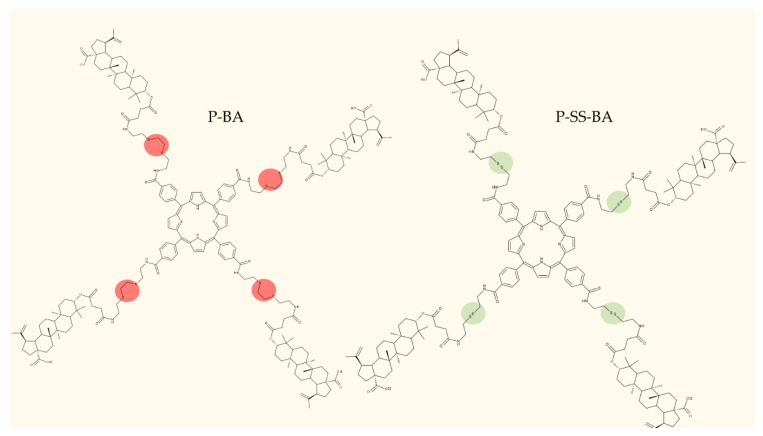
The chemical structure of P-BA and P-SS-BA (the red circle highlights the unbreakable bonds and the green circle highlights the breakable disulfide bonds in the given conditions).

The general approach to optimize BA bioavailability through chemical derivatization is to graft hydrophilic groups to the core structure in order to increase its hydrophilicity. Thereby, Sylla et al. obtained a series of C3-O-rhamnose-BA conjugates and tested their in vitro effects against DLD-1 (colorectal cancer) and WS1 (skin fibroblast) cell lines. Among the evaluated bioconjugates, compound BA 10, 3-O-α-L-rhamnopyranosyl-(1->4)-α-L-rhamnopyranosyl-BA, exerted selective and superior cytotoxicity against colorectal cancer cells (DLD-1 cell line, IC_50_ = 5 µM) compared with BA, while the tri- and tetra-rhamnose derivatives expressed no sign of cytotoxicity in tested cells [59]. This loss of cytotoxicity may be explained by the strong increase in hydrophilicity noticed for the tri- and tetra-rhamnose-BA derivatives, which could impede their penetration through the membrane of cancer cells. Contrary to this approach, Ozdemir et al. synthesized a phytosteroid-BA bioconjugate (BA 11) by linking diosgenin at the C3-OH position of BA through a 1,4-disubstituted-1,2,3-triazole; its cytotoxicity was assessed in vitro against CEM (leukemia), HeLa (cervical cancer), and HCT 116 (colon cancer) cell lines. The study revealed that, while the bioconjugate was inactive against tested cells (IC_50_ > 50 µM), a reaction intermediate, the C28-benzyl ester of the BA-diosgenin bioconjugate (BA 12), showed promising effects against the CEM cell line, with an IC_50_ = 6.5 µM [60]. This study shows that a bioconjugate obtained from two lipophilic structures, highly lipophilic itself, possesses significant cytotoxicity against certain cancer cells; furthermore, the presence of the benzyl ester moiety at the C28 position of the BA-diosgenin bioconjugate is favorable for the anti-leukemia effect in vitro, results that should be further assessed in vivo. The studies continued with an interesting approach to increase BA’s cell uptake and cytotoxicity by reacting two C3-succinylcystamine derivatives with 5,10,15,20-tetrakis(4-carboxy-phenyl)-21H,23H-porphyrin, resulting in the synthesis of two bioconjugates, P-BA and P–SS-BA (Figure 4). While P-SS-BA generated cytotoxic reactive oxygen species (ROS) when photostimulated in the presence of glutathione (GSH) due to the cleavage of the disulfide bond, the P-BA remained intact. Furthermore, the P-SS-BA uptake and cytotoxicity in 4T1 (breast cancer) cells were evaluated, revealing that the conjugate was internalized by the cancer cells and induced cytotoxic effects with an IC_50_ of 4.25 µM after photostimulation in the presence of glutathione [60]. However, a flaw in the study is the absence of the P-BA uptake and a cytotoxicity assessment that would have provided a better comparison between both the parent compound, BA, and the other bioconjugate, P-SS-BA. 

### 4.3. C28 Modifications

A series of BA-imidazole derivatives were obtained by linking the imidazole heterocycle to the C2, C3, and C28 positions, followed by testing their antileishmanial effects. One derivative containing two imidazole heterocycles linked to C2 and C28 (BA 13) was found to be the most active in vitro and was further associated with miltefosine, an antileishmanial drug, in order to identify a potential synergistic activity; unfortunately, the compound induced a slight decrease in the IC_50_ value compared with miltefosine alone [31]. When analyzing the SAR in the series, it was noticed that the presence of two imidazole heterocycles combined with the oxidation of C3 are favorable for the antileishmanial effect, suggesting that these derivatizations could be further employed for the development of BA-derived antileishmanial drugs. In a similar manner, Meira et al. synthesized BA derivatives through the introduction of various N-containing heterocycles, such as piperidine (BA 14,16,20,21), piperazine (BA 15,17), morpholine (BA 18), and thiomorpholine (BA 19), in the C28 position through an amidic bond; their trypanocidal activity was tested in vitro on infected mouse macrophages [61]. Among the piperidine derivatives, only the p-phenyl-piperidine derivative showed significant activity, suggesting that the substitution of the piperidine heterocycle with aromatic as opposed to allylic moieties favors antitrypanosomal activity; however, the introduction of a 4-(4-fluorophenyl)piperidine resulted in significantly reduced in vitro activity, probably due to increased lipophilicity, which prevents the transmembrane passage. The most active derivative of the series was obtained by introducing a morpholine heterocycle, which presumably improved the molecule’s affinity for the target site. A study supporting this hypothesis was developed by Ibezim et al. [62], who tested the affinity of various morpholine-containing compounds for trypanosomal triosephosphate isomerase (TTI), an essential enzyme for the survival and development of several *Trypanosoma* spp.; the study showed that morpholine derivatives exhibit high affinity for TTI. Collectively, these findings suggest that the morpholine derivatives of BA could represent an important resource for the development of potent antitrypanosomal drugs. 

A combination of various rings, such as piperazine, pyrrolidine, piperidine, cyclopentane, cyclohexane, and benzyl, was introduced to the C28 and/or C3 positions in the BA molecule in order to identify new and improved anticancer agents; their in vitro toxicity was assessed against HeLa (cervical cancer), HepG-2 (hepatocarcinoma), BGC-823 (gastric cancer), and SY-SY5Y (neuroblastoma) cell lines. The results revealed the following aspects regarding the SAR in the series: (1) the esterification of C28 with benzyl alcohol is favorable only when the C3 position simultaneously contains a piperazine heterocycle (BA 22)—otherwise, it leads to inactive derivatives; (2) the oxidation of C3 into a keto group, combined with the introduction of a cyclohexyl-amino-ethyl, cyclopentyl-amino-ethyl, or 1-ethyl-piperidine moiety (BA 23–25), at the C28 position is beneficial for a cytotoxic effect against HeLa and SK-SY5Y cell lines [63]. A BA-C28-pyrrolidine derivative that is capable of releasing nitric oxide (BA 26) was tested against a panel of five cancer cells, B16F10 (murine melanoma), MCF-7 (breast cancer), HCT-116 (colon cancer), A549 (lung cancer), and HepG-2 (hepatocarcinoma), alongside two non-cancer cells, Helf (lung fibroblast) and LO2 (hepatic cell). The results revealed higher cytotoxic effects compared with both BA and cisplatin against cancer cells (IC_50_ = 0.89–7.44 µM), as well as lower cytotoxicity against the tested non-cancer cells (IC_50_ = 17.76–27.21 µM) [64]. Therefore, one may conclude that the derivatization of BA through the esterification of the C28-carboxyle with various heterocycles, in particular, N-containing heterocycles, represents a promising alternative for the synthesis of potent cytotoxic derivatives. This hypothesis was confirmed by Kodr et al., who synthesized and evaluated the anticancer effect of 17 BA-piperazinyl and BA-aminopropyl derivatives obtained through the derivatization of C3 and C28; several derivatives were also marked with blue emitting BODIPY (4,4-difluoro-4-bora-3a,4a-diaza-s-indacene) moieties. The anticancer effect was compared with BA and bevirimate, a BA-derivative with potent anti-HIV activity, which was abandoned in phase two clinical trials due to resistance acquisition. A SAR evaluation was performed on those derivatives that exhibited IC_50_ values within the micromolar range (Table 3) and revealed that BA-pyperazinyl derivatives induce slightly higher cytotoxic effects compared with BA-aminopropyl derivatives; in addition, the esterification of C3 with succinic acid or BODIPY-COOH diminished the overall cytotoxicity, thus suggesting that free C3 (BA 27) plays a key role in the occurrence of cytotoxic activity [65]. Although the study showed clearly superior cytotoxicity compared with BA and beviramat, the same effect was also exerted against normal cells, thus limiting their development as therapeutic compounds. However, BODIPY-BA conjugates (BA 28–29) could be employed in the investigation of the cellular uptake, distribution, and mechanism of action in BA due to their fluorescent properties. This was exemplified in the work undertaken by Krajcovicova et al. [66], who used similar BODIPY-BA derivatives to measure their in vitro uptake into leukemia-derived cells. Other types of fluorescent groups are isatins, or bis-arylidene oxindole moieties, which were used to obtain a bis-arylidene oxindole-BA conjugate (BA 30) and visualize its selective uptake inside several cancer cell lines (mouse melanoma: B16F10; breast cancer: MCF-7 and MDA-MB-231; ovarian cancer: OVCAR-3; pancreatic cancer: PANC-1 and lung cancer: A549) compared with normal NiH3T3 fibroblasts and CHO hamster ovarian cells. This study also showed an increase in cancer cell apoptosis compared with normal cells, which was explained by the selective uptake and subsequent generation of ROS caused by the BA derivative inside the cancer cells [67]. These studies put into perspective the use of BA derivatives labeled with fluorescent moieties as potential biomarkers for pharmacokinetic studies of tritepenic compounds.

In a similar manner to the previously presented betulin, the triazole moiety was exploited for the derivatization of BA; a series of N1-substituted-1,2,3-triazole compounds were used as esterification partners for the C28-carboxyle group, resulting in derivatives with potential anticancer effects. In vitro tests against 4T1 (breast cancer) and MIA-PaCa-2 (pancreatic cancer) cell lines allowed for a SAR analysis, which revealed that the free C3-OH is essential for the cytotoxic activity since its oxidation into the keto group or esterification with succinic acid significantly reduced such effects. Furthermore, it was also noticed that some derivatives containing either pyrazinyl or indolyl moieties fused at the C2-C3 positions did not produce a significant effect on tested cells. Collectively, these findings enabled the selection of four lead molecules, each bearing a free C3-OH group and an N1-substituted-1,2,3-triazole moiety at the C28 position (BA 31–34), all exhibiting IC_50_ values ranging between 0.81 and 2.62 µM [68]. In the same line of investigations, the introduction of N-acyl-1,2,4-triazole or 2′-methylimidazole moieties to the C3 and/or C28 positions triggered potent cytotoxic effects against several cancer cell lines, including HepG-2 (hepatocarcinoma), HeLa (cervical cancer), HT-29 (colon cancer), and PC-3 (prostate cancer); interestingly, the most active derivative of the series, particularly against prostate cancer cells, contains two triazole moieties (BA 35) inserted in the C3 and C28 positions [69], thus suggesting that multiple triazole moieties may be particularly beneficial for BA cytotoxicity enhancement.

The synthesis of new derivatives using butanoic or triethylene glycol as spacers to attach a cationic triphenylphosphonium group at the C28 position of BA was conducted by Nedopekina et al., who introduced the acetylation of the C3 position in some derivatives, thus achieving cytotoxic derivatives (BA 36–40) with IC_50_ values below 3.0 µM against MCF-7 (breast cancer) and TET21N (neuroblastoma) cell lines. The acetylation of C3 alone resulted in one BA derivative with low in vitro cytotoxicity, thus highlighting the key role played by the cationic group in exercising the cytotoxic effect [70]. Likewise, Dubinin et al. used the same spacers to obtain other bioconjugates (BA 41–43; Table 4) [71,72] of BA with lipophilic cation F16 (E-4-(1H-indol-3-ylvinyl)-N-methylpyridinium iodide), which showed superior cytotoxicity in rat thymocytes compared with the parent compounds [73]. The triphenylphosphonium group can also be introduced to the C28 position of BA by using alkyl/alkoxyalkyl groups of variable length as linkers; such a compound, BA 44, has emerged as a lead molecule in terms of cytotoxic properties against MCF-7 (breast cancer) and PC-3 (prostate adenocarcinoma) cancer cells but also against normal HSF (skin fibroblasts) cells. Given the relatively high reduction in cell viability in healthy fibroblasts (IC_50_ = 2.40 µM) compared with the parent BA, the authors reported the preparation of a semisynthetic compound with low selectivity toward cancer cells [74]. The cationic moiety could also be linked directly to the triterpenic core to obtain cationic bioconjugates of BA. Through this approach, Kraft et al. obtained a series of triterpenoid-safirinium conjugates by directly linking the safirinium moiety to the triterpenoid scaffold; in vitro tests on malignant and non-malignant cell lines revealed that one such BA-safirinium conjugate (BA 45) exerted the highest cytotoxicity against all tested cancer cell lines (melanoma: A375; colorectal carcinoma: HT-29; breast cancer: MCF-7; ovarian cancer: A2780 and squamous carcinoma: FaDu) with an IC_50_ ranging from 3 to 7.5 µM. The active conjugate differed from the other compounds by being substituted with two hexyl fragments of the safirinium P moiety, which is linked through a piperazinyl ring at the C28 in BA scaffold, a substitution that could favor the transmembrane passage compared with substitution with ethyl or dodecyl moieties. Interestingly, while it is well known that BA possesses mitochondria-targeted effects and substitution with cationic moieties usually boosts such effects, the study revealed that the safirinium core could not penetrate the cell membrane; therefore, its BA-conjugate accumulated inside the endoplasmic reticulum, which seems to mediate its cytotoxicity [75]. Collectively, these findings highlight the favorable impact of introducing cationic moieties to the triterpenic scaffold in order to achieve cytotoxic effects against various cell lines.

One of the most frequent approaches in drug design consists of the synthesis of bioconjugates containing two bioactive components in order to reach an overall increased therapeutic effect, based on the combination of two individual mechanisms of action. In this line of investigation, Bori et al. synthesized an ester bioconjugate (BA 46) of bevirimate with azidothymidine, a nucleoside reverse transcriptase inhibitor that is linked through a 1,2,3-triazole moiety in the C28 carboxyl group of bevirimate; the bioconjugate revealed superior anti-HIV properties compared with the semisynthetic triterpenoid [76]. Within the modern trend of drug repurposing, such BA-azidothymidine-ester derivatives have also been investigated as anticancer agents; when tested against two tumor cell lines, KB (papilloma) and HepG2 (hepatocarcinoma), four bioconjugates (BA 47–50) obtained via click reaction between BA and azidothymidine, have shown increased cytotoxicity (IC_50_ = 5.9–8.5 µM) against either KB or HepG2 cells compared with the parent compounds. Of note, two of these compounds were also simultaneously subjected to C3 structural modifications: esterification with succinic acid (BA 48) and oxidation into the keto group (BA 50), thus suggesting that these alterations could add to overall cytotoxicity against tested cells [77]. Furthermore, the amide analogs of the four selected compounds were also evaluated against KB and HepG2 cells, revealing a marked decrease in cytotoxicity compared with the parent triterpene; this effect could be attributed to the ease of ester bond hydrolysis compared to amidic ones, which, in turn, allow for the rapid formation of the active components, BA and azidothymidine, inside cancer cells [78]. This hypothesis was validated by other studies as well; a series of ester bioconjugates of BA with artesunic acid and ferroquine, respectively, were synthesized and tested by Karagoz et al. against *Plasmodium falciparum*-infected erythrocytes and human cytomegalovirus (HCMV) hosted inside human fibroblasts. The study showed that one derivative obtained through the esterification of the C3 position of BA with artesunic acid (BA 51) exhibits a potent antimalarial and antiviral activity. Although the derivative showed stronger antimalarial effects compared with BA, the compound was less potent compared with artesunic acid and chloroquine phosphate, the latter being used as a standard drug for the antimalarial effect. Moreover, the antiviral effect of the same derivative was superior to both BA and the standard antiviral drug ganciclovir [33].

Previous studies have found the combination of BA and cisplatin to exert a synergic cytotoxic effect against several cancer cell lines [79,80]. In order to achieve an even higher cytotoxic effect, Emmerich et al. covalently linked the two active biocomponents, thus obtaining 3-O-acetyl-BA-cisplatin bioconjugates; however, their cytotoxicity dramatically decreased compared with parent molecules when tested against 518A2 (melanoma), A2780 (ovarian cancer), A549 (lung carcinoma), MCF-7 (breast cancer), and 8505C (anaplastic thyroid) cancer cells. Interestingly, when the reaction intermediates were also tested, 3-O-acetylbetulinic (2-(2-aminoethyl)aminoethyl)amide was found to exert superior cytotoxic activity (IC_50_ = 1.30–2.24 µM) compared with BA, thus suggesting that C3 acetylation combined with C28-grafted linear alkyl amide could enhance the transmembrane passage and, consequently, the activity of the bioconjugate [81]. Continuing their research, the same group synthesized another 3-O-acetyl-BA-cisplatin bioconjugate through an amide link in the C28-COOH group, aiming for anticancer effects against three human glioma cell lines, namely U251MG, LN229, and U251MG. However, similar to the previous study, the resulting derivative (BA 52) failed to provide improved cytotoxicity compared with the parent compounds, while a reaction intermediate, acetyloxy-BA-28-[2-(2-aminoethyl)aminoethyl]amide, significantly reduced cell viability in all cancer cells [82]. These in vitro findings suggest that, despite the reported synergistic effect exerted by the association between BA and cisplatin, the synthesis of their bioconjugates might not represent a suitable alternative to improving their combined cytotoxicity. However, since in vitro conditions do not identically replicate the in vivo environment, further in vivo studies should be developed to better assess the potential of these bioconjugates.

**Figure 5:**
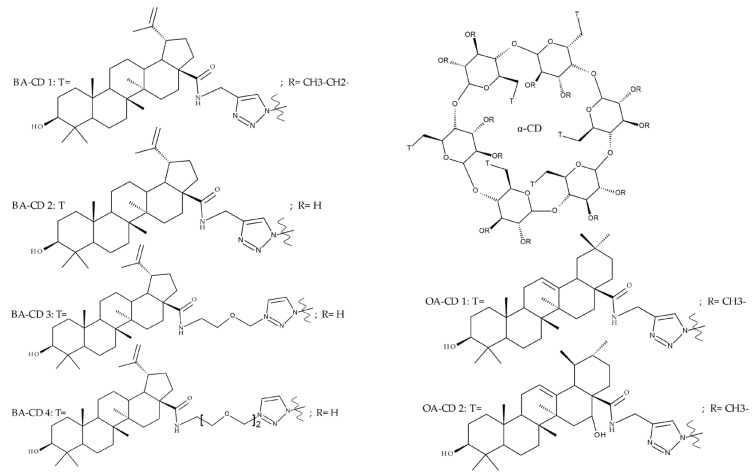
Derivatives of BA and OA with alpha-CD.

Given the assumption that the poor BA oral bioavailability is caused by its low water solubility, BA was conjugated with macromolecular compounds, such as cyclodextrins (CD), in order to achieve higher hydrophilicity. Although cyclodextrins are frequently used to incorporate lipophilic molecules, such as triterpenic acids, inside their inner cavity, the outer surface could also be used to obtain numerous derivatives with enhanced biological activity [83]. One of the chemical reactions employed for the synthesis of such derivatives is a copper-catalyzed “click” reaction [84] between two building blocks, an alkynic triterpene and an azide-derived CD. A 1,2,3-triazole moiety is formed as a result of the click reaction, which can further contribute to the chemical stability of the resulting derivative [85]. Using this approach, Chen et al. synthesized a per-O-acetylated-α-CD-BA conjugate, BA-CD 1 (Figure 5), obtained from BA and per-O-acetylated α-CD linked through a 1,2,3-triazole moiety; the bioconjugate revealed a potent antiviral effect against the A/WSN/33 strain of the influenza virus (IC_50_ = 5.20 µM) [86]. Continuing this line of investigation, the same research group synthesized a series of similar per-O-acetylated–CD-BA conjugates using per-O-acetylated α-, β-, and γ-CD and introducing an oligo-(ethylene glycol) of various lengths between the newly formed 1,2,3-triazole moiety and BA; they tested their antiviral activity against the above-mentioned strain of the influenza virus. A SAR analysis showed that the most potent antiviral activity could be identified for α-CD derivatives containing small oligo-(ethylene glycol) chains (BA-CD 2–4), while the β- and γ-CD derivatives showed either a decreased or total absence of antiviral activity [87]. The aspect that clearly stands out in the above-mentioned studies is the increased antiviral activity of per-O-acetylated α-CD-BA derivatives compared with their β- and γ-CD analogs, thus suggesting that the size of the resulting bioconjugate is an essential parameter for the antiviral activity. This observation is valid for other triterpenes as well; as an example, Tian et al. synthesized per-O-methylated-cyclodextrin-OA conjugates using per-O-methylated α-, β-, and γ-CD connected through a 1,2,3-triazole moiety to the triterpene scaffold and tested their antiviral activity against the same A/WSN/33 strain of the influenza virus. Similar to the previous studies, two per-O-methylated-α-CD-BA conjugates (OA-CD 1: IC_50_ = 4.70 µM and OA-CD 2: IC_50_ = 6.50 µM) differ only by the presence of a methyl or hydroxyl substituent on the triterpenic scaffold, showed the most promising antiviral effects without associated toxicity against host cells, thus suggesting the occurrence of high antiviral selectivity that should be further investigated [88].

Although the literature on the topic highlights many BA derivatives with superior biological activity, studies that have obtained derivatives with diminished activity compared with either BA or standard cytotoxic drugs should also be brought to attention in order to limit the waste of resources for fellow researchers. For example, Khlebnikova et al. obtained C28-modified F-containing-2-acylcycloalkane-1,3-diones-BA conjugates with lower cytotoxicity against MCF-7 (breast cancer) and U-87 MG (glioma) cells compared with doxorubicin [89]. Similarly, Ali et al. showed that the C28 BA-γ-butyrolactone and BA-benzyl-ester are less cytotoxic against the HCT116 (hepatocarcinoma) cell line compared with BA [90]. These structure modulations should be considered in future studies in order to orient investigations from an early stage.

### 4.4. C30 Modifications

Given the reported key pharmacophore role played by the triazole moiety, it was employed by Sidova et al. in the synthesis of BA-4-substituted-1,2,3-triazole derivatives via Cu-catalyzed Huisgen 1,3-cycloaddition to the C30 allylic group using C3 acetylated BA-derivatives as starting compounds; when tested in vitro against a panel of eight cancer and two normal cell lines, compound BA 53, containing an acetylated C3 and a 4-(2-formylphenyl)-1,2,3-triazole in the allylic position, showed significant cytotoxicity as well as selectivity against several types of leukemia cells such as CCRF-CEM (IC_50_ = 3.3 µM), K562 (IC_50_ = 3.6 µM), and K562-TAX (IC_50_ =3.9 µM) [91]. The insertion of two other N-containing heterocycles, pyridine (BA 54) and tetrahydropyridine (BA 55) into C30 in the molecule of 28-O-methyl-BA resulted in improved antibacterial effects against *Staphyloccocus aureus* compared with the parent compound, thus indicating that aromatic or liphatic N-containing heterocycles could be used for the synthesis of antimicrobial BA derivatives [92].

Another previously reported promising approach was the insertion of sugar moieties on triterpenoid scaffolds; therefore, among a series of C30-N-acetyl-D-galactosamine-triterpenes conjugates, two BA derivatives (BA 56–57) were selected and investigated in terms of cytotoxicity, revealing, however, inferior properties compared with BA and doxorubicin; despite this apparent failure in research, the two compounds showed an improved affinity for the asialoglycoprotein receptor (ASGPR-receptor), expressed mainly in mammalian hepatocytes, which is currently the target for the development of hepatotropic drugs [93]. Furthermore, when the N-acetyl-D-galactosamine moiety was inserted instead at C28 in the molecule of 3-O-acetyl-BA, the resulting bioconjugate (BA 58) displayed similar cytotoxic effects [94], thus suggesting that, while this derivatization increases the affinity for hepatic cells, it also reduces cytotoxicity. In light of these findings, it would be interesting to further investigate the anti-inflammatory effects of such derivatives in hepatic cells, as the anti-inflammatory properties of the parent BA are already well recognized.

In an effort to identify new pharmacophore groups for the chemical manipulation of BA, a series of biotinylated BA bioconjugates were synthesized through derivatizations in C3, C28, or C30; the bioconjugates revealed the same level of cytotoxicity as the parent compounds against CCRF-CEM (leukemia) and HCT116 (colorectal carcinoma) cell lines [96]; however, the study provided valuable information for future studies in terms of SAR, indicating that BA biotinylation does not alter its mechanism of action.

### 4.5. A-Ring-Fused Heterocyclic Derivatives

BA conjugates can also be obtained by fusing various heterocycles to the C2-C3 bond in ring A of the triterpene scaffold; among such conjugates, Xu et al. identified one derivative containing a pyrazole fused to the A-ring of BA, SH479, which showed remarkable activity regarding osteoclast differentiation compared with BA and, therefore, could be used in the treatment of osteoporosis [95]. In addition. later studies developed by independent research groups also revealed its therapeutic potential against other pathologies, such as triple-negative breast cancer [97], autoimmune encephalomyelitis [98], and arthritis [99]. Isoxazole and oxadiazole derivatives synthesized in a similar manner but starting from 23-hydroxy-BA were tested against a panel of five cancer cell lines, including HL-60 (leukemia), BEL-7402 (hepatocarcinoma), SF-763 (glioblastoma), HeLa (cervical cancer), and B16 (mouse melanoma). The study identified two BA derivatives and one isoxazole derivative bearing an amidic bond at C28, and the other resulted from opening the isoxazole ring and bearing a morpholine moiety at C28, which showed the most promising in vitro cytotoxic activity, which was further confirmed in vivo in a mouse xenograft model [100]; the experimental results emphasized the beneficial role of isoxazole in increasing cytotoxicity, in particular, when combined with electron-donating and/or polar substituents grafted at C28, which endows the molecule with an amphiphilic character. Collectively, these findings emphasize that the extension of the BA scaffold through the modulation of ring A could lead to derivatives of therapeutic value.

## 5. Betulonic Acid

**Figure 6:**
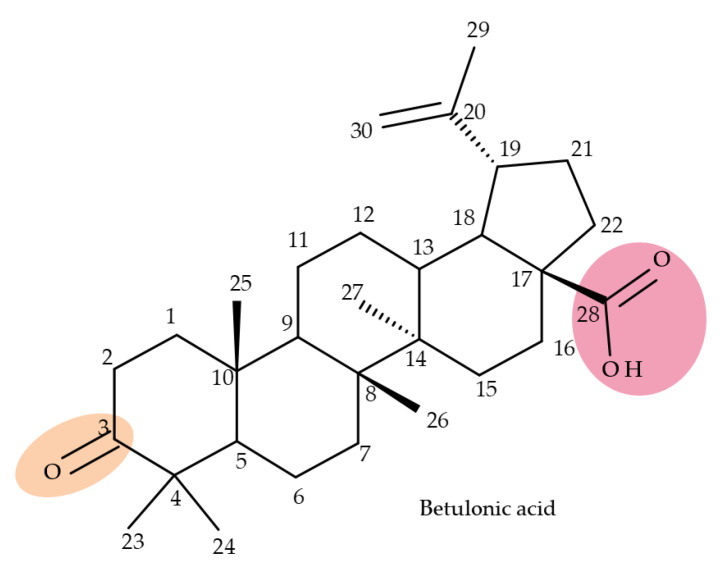
Frequent targets for the derivatization of betulonic acid.

Betulonic acid (BoA; 3β-3-Hydroxy-lup-20(29)-en-28-oic acid; Figure 6) is one of the most important pentacyclic lupine-type triterpenoids and has been found to be a promising candidate as an antitumor agent since it can inhibit the growth of different types of tumor cells. BoA also induces several biological effects, such as antiviral, antimicrobial, anti-inflammatory, antioxidant, hepatoprotective, and immunostimulant activities [101].

The introduction of piperidine or pyrrolidine nitroxyl to the C28 position of the BoA molecule through the formation of amidic bonds may result in the occurrence of optimized hepatoprotective properties compared with the parent compound. Sorokina et al. reported the synthesis of a piperidine–nitroxide derivative (BoA 1; Table 5) and a pyrrolidine–nitroxide derivative (BoA 2), obtained through the esterification of C28 in BoA, which exert anticholestatic properties in a model of cholestatic hepatitis in mice. Furthermore, the piperidine derivative was also able to increase the lifespan of animals without exerting antitumor effects, presumably due to its hepatoprotective activity [102]. The amidic bond was also exploited by Khlebnicova et al., who inserted fluorine-containing aromatic or aliphatic heterocycles into the C3 or C28 positions of BoA and further assessed their anti-inflammatory and antioxidant in vivo activity. Although most derivatives exhibited significant anti-inflammatory and antioxidant effects, the leading compound of the series was BoA 3, which is structurally characterized by the presence of a 2-perfluorobutanoyl-2-cyclopenten-1-one moiety at the C3 position and a methylated carboxyle at C28. Moreover, its cytotoxicity against three cell lines (leukemia: CEM-13 and U-937 and testicular epithelial mouse cell: TM-4) was also assessed, revealing inferior potency compared to the parent compound [103]; in this case, the introduction of fluorine-bearing moieties led to compounds lacking cytotoxic properties, contrary to the previously discussed BA fluorine derivatives, results that could be partly explained by the specific features of the cell lines used in the cytotoxicity assessment. The increase in the anti-inflammatory activity was also attained by Popovov et al., who synthesized derivatives of various lupane-triterpenes bearing 1,3,4- and 1,2,5-oxadiazoles connected to the C28-COOH position through an amino acid chain. Most 1,3,4-oxadiazole-BoA derivatives (BoA 4–5) significantly exceeded the activity of the parent compound, while the 1,2,5-oxadiazole derivatives showed inferior anti-inflammatory activity compared with BoA [104], suggesting that the presence of a 1,3,4-oxadiazole moiety linked through an amino acid chain to the triterpenic acid favors an anti-inflammatory in vivo effect. 

Another important heterocycle found in BoA derivatives with anti-inflammatory properties is the previously mentioned triazole. Vasilevsky et al. synthesized several BoA derivatives with anti-inflammatory activity, containing a substituted 1,2,3-triazole moiety at the C28 position; the activity has been attributed both to the triterpenoid scaffold and the sidechain containing the triazole moiety. The SAR evaluation revealed that the anti-inflammatory effect increases upon replacing the hexyl substituent of the triazole moiety with an aryl substituent, in particular, an acetylphenyl group, which appears in the structure of the lead compound, BoA 6 [105]. Similarly, Lipeeva et al. obtained several BoA conjugates with coumarins through a 1,2,3-triazole moiety; among the tested compounds, a BoA-oreoselone derivative (BoA 7) showed a marked anti-inflammatory activity that was annulled by the introduction of an additional substituent at the C9 position of the oreoselone moiety (BoA 8) [106]. 

The triazole heterocycle was also highlighted as a key pharmacophore in the structure of various BoA derivatives with anticancer properties. A series of derivatives of 3-acetylbetulin and betulone containing a 1,2,3-triazole moiety were synthesized through a “click” reaction in the C28-COOH position of triterpenic compounds and assessed in terms of cytotoxicity against three cancer cell lines, C-32 (melanoma), T47D (breast cancer), and SNB-19 (glioblastoma). The study reported that, as a general rule, the substitution of the triazole moiety with carbonyl or acyl substituents such as acetyl induces stronger anticancer activity against the C-32 amelanotic melanoma cell line compared with substitution with aryl substituents. Moreover, a BoA conjugate with 3′-deoxythymidine-5′-yl (BoA 9) proved to be the lead compound against the SNB-19 (glioblastoma) cell line, with an IC_50_ = 0.17 μM, an effect that has been associated with its decrease in lipophilicity [107]. In addition to the triazole moiety, other heterocycles are also able to increase the anticancer properties of BoA derivatives, as shown by Borková et al., who extended the structure of the triterpenic acid by fusing an aminothiazole ring to the C2-C3 bond in ring A (BoA 10); cytotoxic tests against eight cancer (leukemia: CCRF-CEM, K562, CEM-DNR, and K562-TAX; lung cancer: A549; colon cancer: HCT116, HCT116p53−/−; osteosarcoma: U2OS) and two non-cancer cell lines (fibroblasts: BJ and MRC-5) indicated significant anticancer activity only against the CCRF-CEM cell line, with an IC_50_ = 2.4 μM [108]. Another example is the pyridine moiety, which was introduced by Khusnutdinova et al. at C2 in the structure of 28-Oxo-allobetulone (BoA 11) through an ethylene linker, resulting in the synthesis of 3-pyridylideno-28-oxo-allobetulone (BoA 12), which was further tested in vitro against a panel of 60 cancerous cell lines. The experimental results showed that BoA12 was active against several cancer types, including leukemia, its activity being triggered by the presence of the 3-pyridylideno-fragment; this hypothesis was validated by the lack of activity reported for its 4-pyridylideno-analog. The authors concluded that the position of the nitrogen atom in the pyridyl fragment of the semisynthetic derivatives exerts a strong influence on both the selectivity and potency of the cytotoxic effect [109]. Unfortunately, in certain cases, the introduction of heterocycles to the structure of BoA could lead to activity loss, as reported by Kazakova et al., who identified a BoA dihydropyrazine derivative (BoA 13) as completely inactive against tested cancer cells as opposed to branched-amino-BoA and BA derivatives [110]. 

A series of indole- and pyrazine-fused ring A derivatives of lupane, oleanane, ursane, and dammarane-type triterpenoids were synthesized from their corresponding 3-oxo-derivatives; the C2–3 indole triterpenoid derivatives were obtained by Fischer indolization of 3-oxo phytocompounds with phenylhydrazine, while the C2–3 pyrazine derivatives were obtained by reacting the 3-oxo compounds with ethylenediamine. The newly synthesized hybrids were tested for their inhibitory activity against α-glucosidase, allowing some conclusions to be drawn in terms of SAR; thus, a SAR analysis revealed that the main triterpenic core significantly impacts the inhibitory activity, as indicated by the higher efficacy of lupane, oleanane, and ursane indole derivatives compared with their allobetulin and dammarane indole analogs. In addition, the activity of 2,3-indolotriterpenoids exceeded that of their corresponding 3-oxo-derivatives; particularly, 2-3 indolobetulonic acid (BoA 14) exhibited strong α-glucosidase inhibitory effects compared with BoA, proving to be 221-fold more active than acarbose [111].

## 6. Ursolic Acid

**Figure 7:**
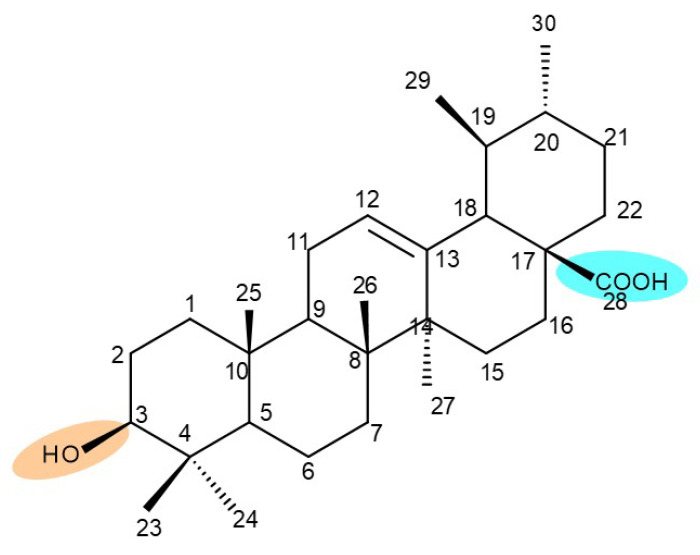
Frequent targets for the derivatization of ursolic acid.

Ursolic acid (UA) is an ursane-type triterpenoid found in nature either in its free form or as the aglycone of triterpenoid saponins; it can be extracted from a large a variety of plants, but especially from apple juice; moreover, it was reported to exhibit a wide spectrum of pharmacological activities, including antitumor, antioxidant, anti-inflammatory, cardioprotective, antiviral, etc., effects [112]. Taking into account its poor pharmacokinetics, this represents a huge drawback in drug formulation, causing poor oral absorption and bioavailability; many researchers have focused on developing UA derivatives with enhanced therapeutic effects in the last two decades [113]. The most accessible positions for chemical derivatization are indicated in Figure 7. 

The rhamnose moiety, previously reported as part of the pharmacophore group due to the cytotoxic activity of other triterpenes, was also used for the derivatization of ursolic acid; the resulting saponins were subjected to in vitro tests against human colorectal adenocarcinoma (DLD-1) cells and healthy human skin fibroblasts, while their anti-inflammatory activity was quantified by assessing the NO production. The experimental data showed that the presence of a single or di-rhamnose unit did not improve the overall cytotoxic activity of the molecule compared with ursolic acid; moreover, the presence of three- and tetra-rhamnose units completely abolished the compounds’ cytotoxicity. In contrast, all derivatives, UA 1–4 (Table 6), effectively inhibited the NO overproduction, a parameter consistent with inflammatory events, regardless of the number of rhamnose units; the EC_50_ values ranged between 9.8 and 16 μM, indicating that the anti-inflammatory effect of each compound depends on the number of sugar units attached to the main skeleton [59]. 

The relevance of nitrogen-containing heterocycles regarding the cytotoxic effects of semisynthetic triterpenes was confirmed for ursane and lupane derivatives as well. 1,3,4, and 1,2,5-oxadiazole and furoxane moieties were attached to the triterpenoid scaffold through plain or modified triazole linkers, which form esteric bonds with the C3 or C28 carboxyle groups. Some derivatives contained a combination of triazole and oxadiazole functions attached to the ursane and lupane skeletons via a succinate linker. The furoxane derivatives presented an additional ester linkage bond that connected the triazole linker and the furoxane fragment to the C-28 position of the triterpenoid frame. In vitro tests against several cancer cell lines revealed the strong cytotoxic activity in MCF7 cells (breast carcinoma) of compounds UA 5 (IC_50_ = 22.9 µM) and UA 6 (IC_50_ = 1.55 µM) containing one furoxan ring linked to the triazole moiety. In addition, UA 6, obtained from UA 5 by acetylating the C-3 hydroxyl function, showed higher selectivity (S.I. = 6.72) for MCF-7 cells compared with both ursolic acid and doxorubicin. However, their biological activity was less effective against U-87-MG (multiforme glioblastoma), HepG2 (liver carcinoma), and A549 (lung carcinoma) cells compared with the parent compound. Moreover, the SAR analysis revealed that the introduction of an additional ester spacer between the triazole group and the main skeleton led to a significant decrease in biological activity. Betulonic acid derivatives with furoxan functions linked to the main skeleton through an amino acid linker showed no cytotoxic properties against the tested cancer cell lines. However, 1,2,3-triazole and 1,2,5-oxadiazole betulone hybrids showed selective cytotoxic activity against MCF-7 cell lines compared with the parent compound, demonstrating the importance of the triazole and oxadiazole functions toward the biological activity of the compounds [114]. 

As mentioned previously, the synthesis of triterpene–BODIPY derivatives has become an important tool for identifying and investigating the effect of the tested phytocompounds on different types of malignant and non-malignant cells. Therefore, Brandes et al. obtained a series of triterpenoid BODIPY FL derivates; the compounds were derivatized from C3-OH acetylated BA, OA, UA, GA, and Bet, followed by reactions with oxalyl chloride and piperazine-yielded amides. Initially, the authors obtained C-28 amidic derivatives or C-28 piperazinyl derivatives. The BODIPY FL function was predominantly attached to the C-28 position of the triterpenoid skeleton through an ester bond or to the piperazinyl function through an amidic bond. In a few derivatives, the BODIPY function was attached to the C-3 hydroxyl position through an ester bond, while the C-28 hydroxyl was acetylated. The compounds were further tested against several cancer cell lines, including A375 (melanoma), MCF-7 (breast cancer), HT-29 (human colorectal adenocarcinoma), and A2780 (ovarian adenocarcinoma). The results revealed that the parent triterpenoids showed little to no cytotoxicity against any cell line. However, the most effective cytotoxic effects against all cancer cell lines were proven for all 3-O-acetylated piperazinyl derivates amidic derivatives. The BODIPY hybrids exhibited little cytotoxicity; only the 3-O-acetyl-betulinic acid-derived BODIPY FL conjugate of UA (UA 7) exhibited a significant cytotoxic effect against the MCF-7 cell line with an EC_50_ = 11.3 µM, but no toxicity against the other cell lines. The SAR also revealed the importance of the length of the linker; hence, the piperazinyl and homopiperazinyl-spacered derivatives showed higher cytotoxic activity compared with ethylenediamine-spacered hybrids [115].

## 7. Maslinic and Corosolic Acids

**Figure 8:**
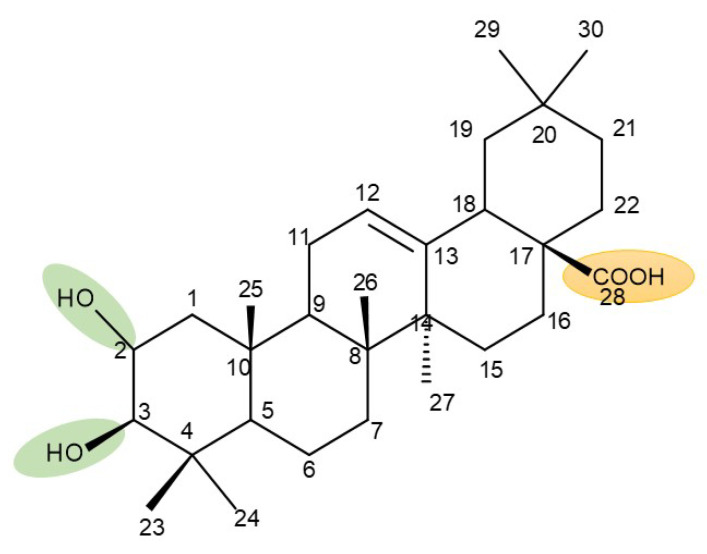
Frequent targets for the derivatization of maslinic acid.

**Figure 9:**
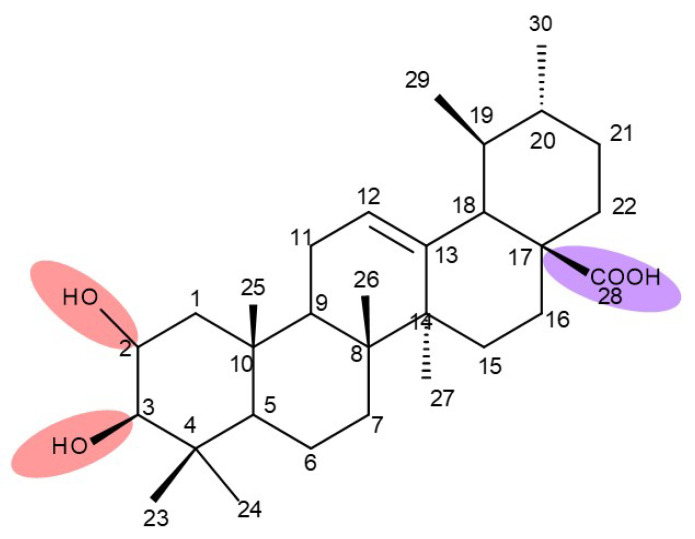
Frequent targets for the derivatization of corosolic acid.

Maslinic acid (MA) is a pentacyclic triterpene occurring in several vegetables and fruits that are part of the Mediterranean diet, exerting a wide spectrum of pharmacological activities such as antitumor, antidiabetic, cardioprotective, neuroprotective, antiparasitic, and growth-stimulating effects [116]. Corosolic acid (CA) is also a pentacyclic triterpene found predominantly in *Lagerstroemia speciosa* L. (banaba) with significant pharmacological potential in treating diabetes due to its insulin-like properties, which made it known as phyto-insulin [117]; in addition, corosolic acid acts as an anti-inflammatory and anti-neoplastic agent [66]. The most frequently modified chemical positions in the molecules of the two acids for the purpose of improving their pharmacological profiles are indicated in Figure 8 and Figure 9. 

**Figure 10:**
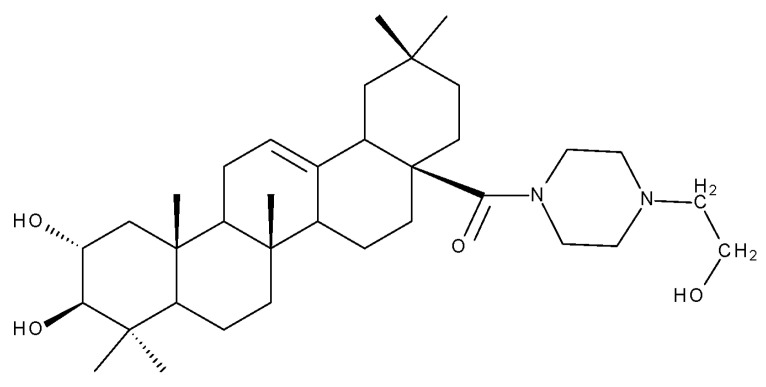
The chemical structure of MA 1.

One of the biggest challenges in managing type 2 diabetes is to control the amount of carbohydrate intake; inhibitors of α-glucosidase might ameliorate hyperglycemic episodes by inhibiting the conversion of oligosaccharides into monosaccharides, which, in turn, trigger high blood sugar. Unfortunately, conventional α-glucosidase inhibitors fail to provide high patient compliance due to several side effects, thus creating the need to develop more natural alternatives to modulate blood sugar levels [118]. Such natural alternatives may consist of semisynthetic derivatives of maslinic and corosolic acids; Liu et al. designed and synthesized a series of twenty-two C28-modified maslinic acid (MA) and corosolic acid (CA) derivatives with saturated nitrogen heterocyclic moieties (1-deoxynojirimycin or piperazine) that were further evaluated in vitro in order to establish their activities as α-glucosidase inhibitors. 1-deoxynojirimycin derivatives were obtained by coupling the 1-deoxynojirimycin moiety at the C-28 position on the lupane skeleton through a carbon chain linker, while the piperazine derivatives were synthesized by attaching the piperazine segment either directly at C-28 or through an amino group. The results showed that MA piperazine derivatives exerted higher α-glucosidase inhibitory activity compared with their CA counterparts. Maslinic acid derivative MA 1 (Figure 10), obtained by attaching a piperazine moiety with one free hydroxyl to C-28, showed higher inhibitory activity (IC_50_ = 499.06 μM) compared with acarbose (IC_50_ = 606 μM), thus revealing the importance of the free hydroxyl function in the piperazine ring regarding the hypoglycemic activity. This behavior led the authors to the conclusion that the presence of a free hydroxyl or amino group in the piperazine moiety attached to the main skeleton significantly enhances the α-glucosidase inhibition. In a series of 1-deoxynojirimycin derivatives, it was noticed that CA conjugates exerted higher α-glucosidase inhibitory activity compared with MA derivatives, while both were more effective than the parent compounds. Moreover, a SAR assessment revealed that the length of the carbon chain linker plays a crucial role in the hypoglycemic activity of the compounds; hence, a longer hydrophobic carbon chain causes a decrease in the inhibitory activity [119].

## 8. Oleanolic Acid

**Figure 11:**
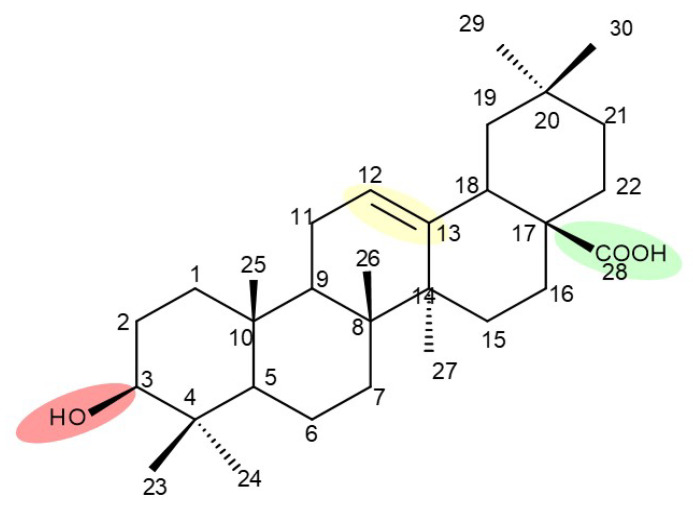
Frequent targets for the derivatization of oleanolic acid.

Oleanolic acid (OA, 3-beta-hydroxylean-12-en-18-oic acid) is an oleanane-type pentacyclic triterpenoid that can be found in nature as a free acid or as the aglycone of triterpenoid saponins [120]. The phytocompound has received much attention due to the fact that it is endowed with a plethora of pharmacological activities, including anticancer, antiviral, antiparasitic, cardioprotective, neuroprotective, and antidiabetic effects. In spite of its promising biological potential, it also exhibits similar bioavailability issues to the other triterpenoid acids; hence, many researchers have obtained several OA analogs through modifications of C-3, C-12, and C-28 (Figure 11), which exhibit improved pharmacological properties [121].

Referring only to the period of time covered by the present review, one must cite the study of Medvedeva et al., who synthesized lupane- and oleanane-type triterpenoids by conjugating azidothymidine fragments to the main skeleton at C-17 and C-20 through 1,2,3- triazole linkers; when tested against several cancer cell lines, including HCT-116 colon, A498 kidney, PC prostate, HL-60 leukemia, RPMI-8226 myeloma, and SR lymphoma cancer cells, the results revealed that most compounds exhibited high cytotoxic activity against all types of cancer cells. Moreover, OA 1 (C-17 1,2,4-oxadiazole oleanolic acid; Table 7) was emphasized as a lead compound, while OA 2 (7-N-carbonyl(pyridine-4-carbohydrazine) oleanolic acid) induced selective cytotoxic activity against HL-60 leukemia cells [122]. 

## 9. Conclusions

Long-life treatments with hardly tolerable synthetic therapies have become a challenge since more and more people are more likely to develop chronic pathologies. Classical therapies have become inconvenient to most patients, who would prefer natural alternatives instead. The most relevant examples in the matter are anticancer therapies, which are not only unbearable for most patients, but also do not always provide favorable outcomes. Moreover, patients who struggle with chronic pathologies such as cardiac diseases or diabetes usually develop other associated conditions. Hence, those patients need to manage their polytherapy, which usually causes pharmacological interactions, leading to an inefficient treatment. Many researchers have explored a plethora of natural alternatives in order to find life-long treatments that can ensure higher patient compliance and fewer side effects. Ever since the discovery of the selective anti-melanoma activity of betulinic acid by Pisha et al., triterpenes have gained much attention, with many studies being focused on analyzing their pharmacological properties. The phytocompounds have proved effective not only against several types of cancer but also revealed potent cardioprotective, neuroprotective, antiviral, antidiabetic, and antidyslipidemic properties. On one hand, they could be regarded as suitable therapeutic agents due to their lack of side effects, high compliance, and versatility of formulation. On the other hand, like many other natural compounds, triterpenes show one major drawback, represented by their low bioavailability, presumably related to their highly lipophilic structure; thus, their poor pharmacokinetic profile prevents the occurrence of in vivo pharmacological effects. One of the most explored directions of research to solve this major shortcoming is the synthesis of triterpenoid derivatives endowed with improved pharmacokinetic parameters compared with the parent compounds. The most frequently employed chemical modulations are the insertion of different types of moieties in the three key positions of the triterpene skeleton, C-3 (hydroxyl/oxo group), C-28 (hydroxyl/carboxyl group), and C-30 (allylic group) or the extension of the main scaffold by fusing various heterocycles with the A-ring of the phytocompound. Many such derivatives also contain linker moieties that connect the triterpenic scaffold with their reaction partners; one such linker, triazole, stood out as a key pharmacophore for the overall biological effect.

Considering the high number of studies that aim to achieve the feasible synthesis of compounds of natural origin with significantly improved pharmacological activities, one may hope to discover suitable alternatives to current treatments. However, there are still important issues that need to be addressed. Firstly, more elaborate in vivo studies have to be designed in order to assess the safety and efficacy of the newly synthesized conjugates; secondly, the underlying mechanism of action for both the semisynthetic derivatives, as well as their natural parent compounds, has yet to be fully elucidated. Despite these challenges, triterpenoid conjugates may represent promising candidates for future clinical trials.

## Figures and Tables

**Table 1:** Chemical structures of lupeol derivatives.

Compound	Structure	Substituents	Reference
**Lup 1–3**	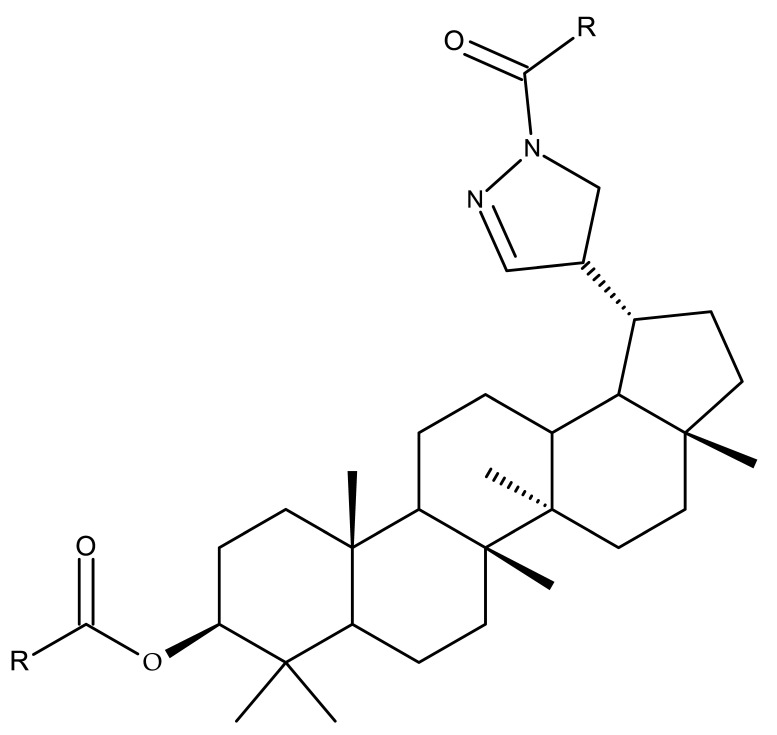	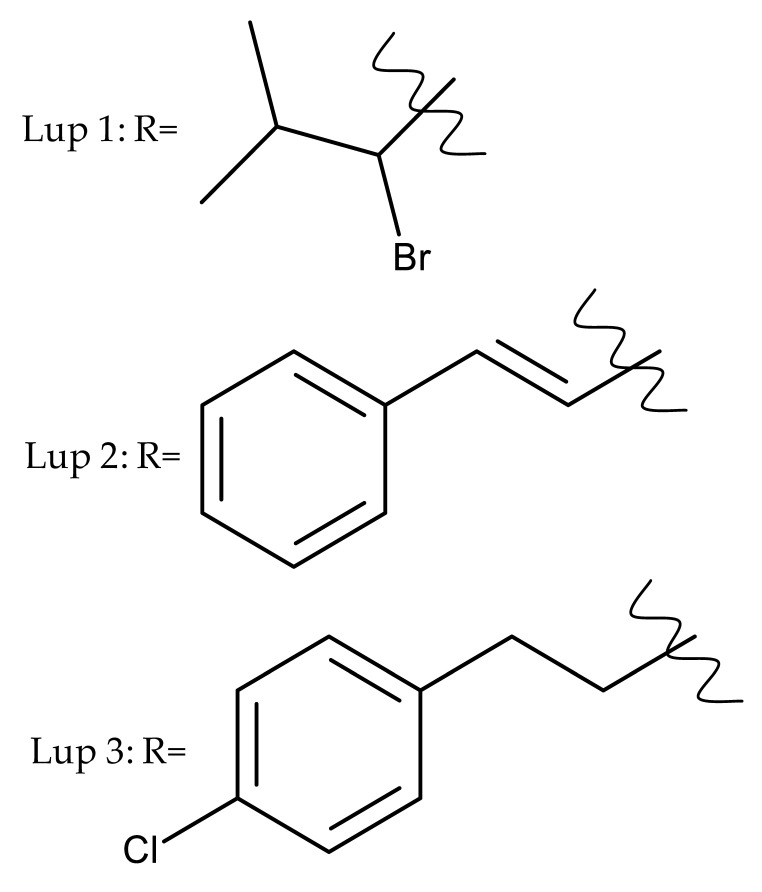	[15]
**Lup 4–7**	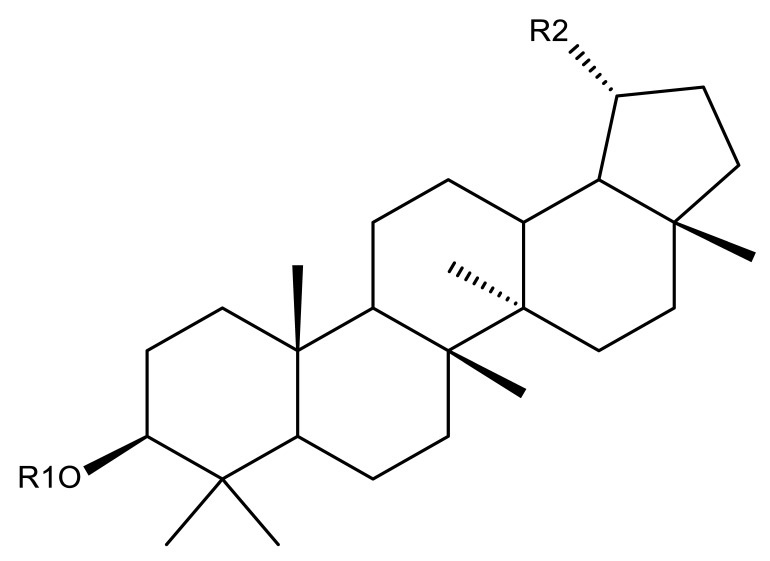	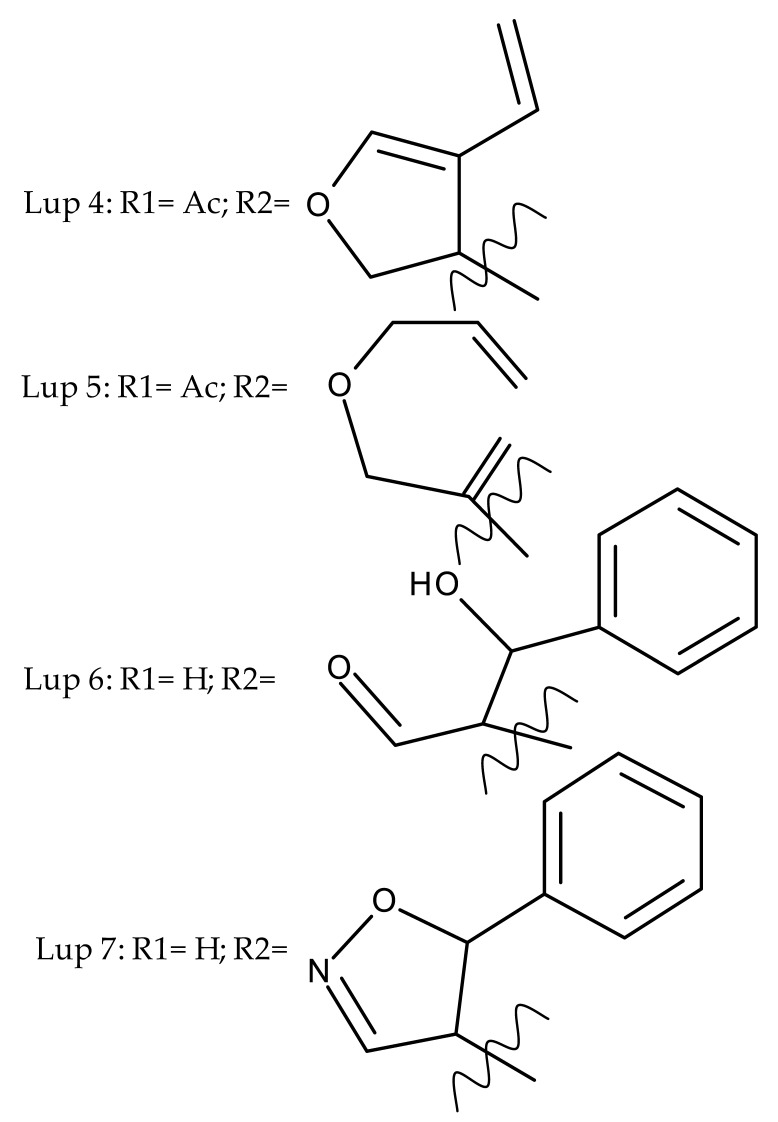	[15]
**Lup 8–9**	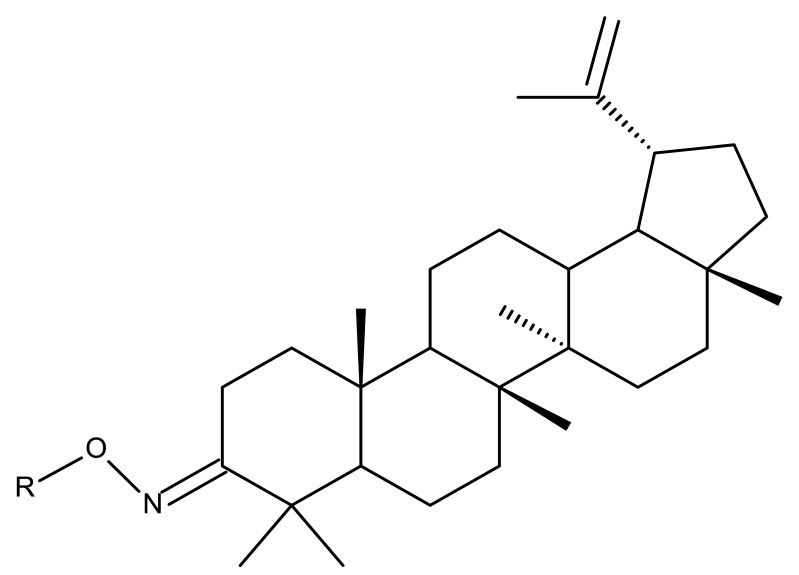	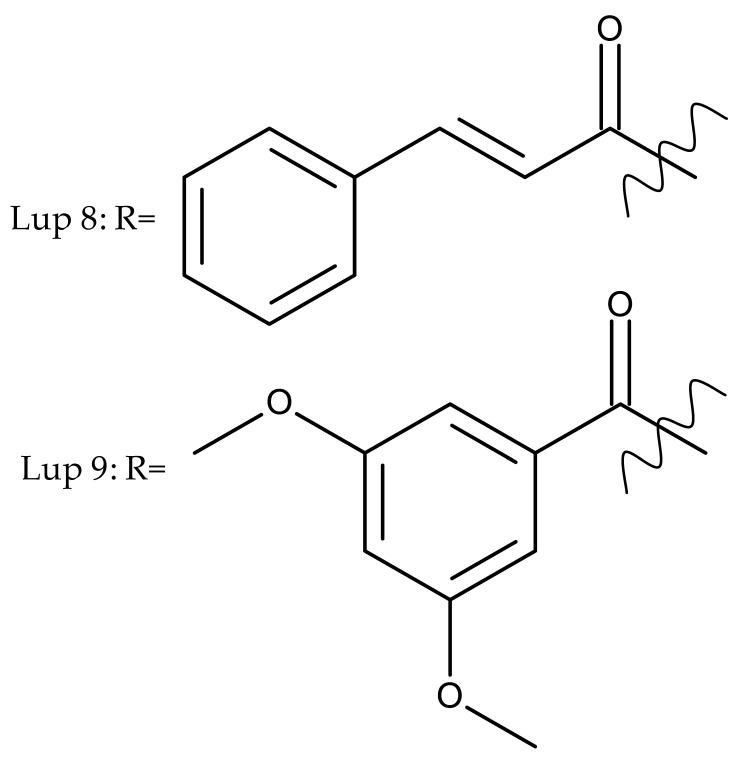	[16]
**Lup 10–11**	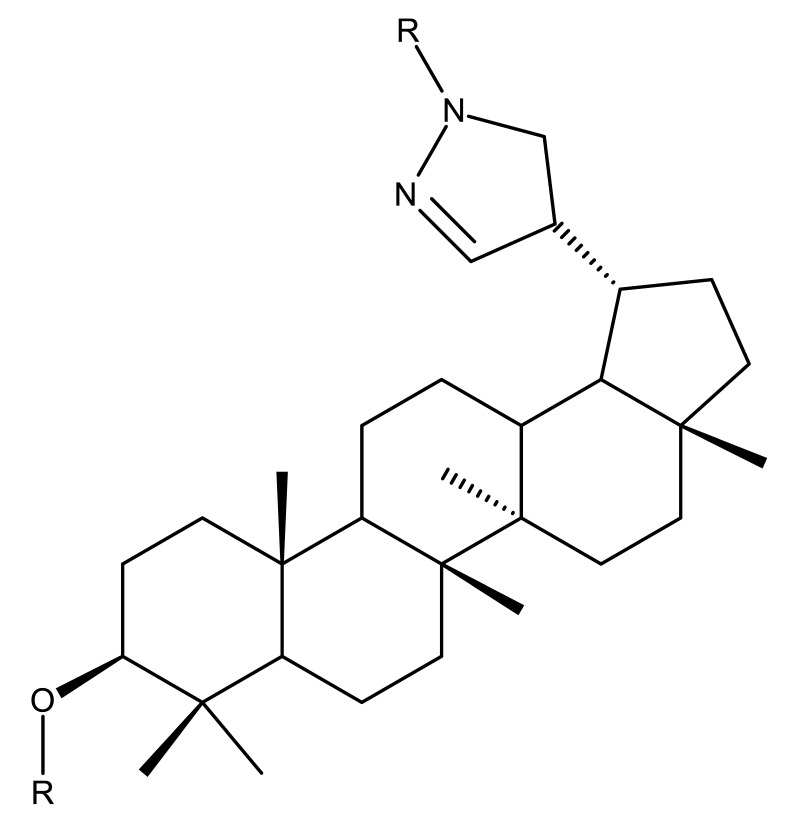	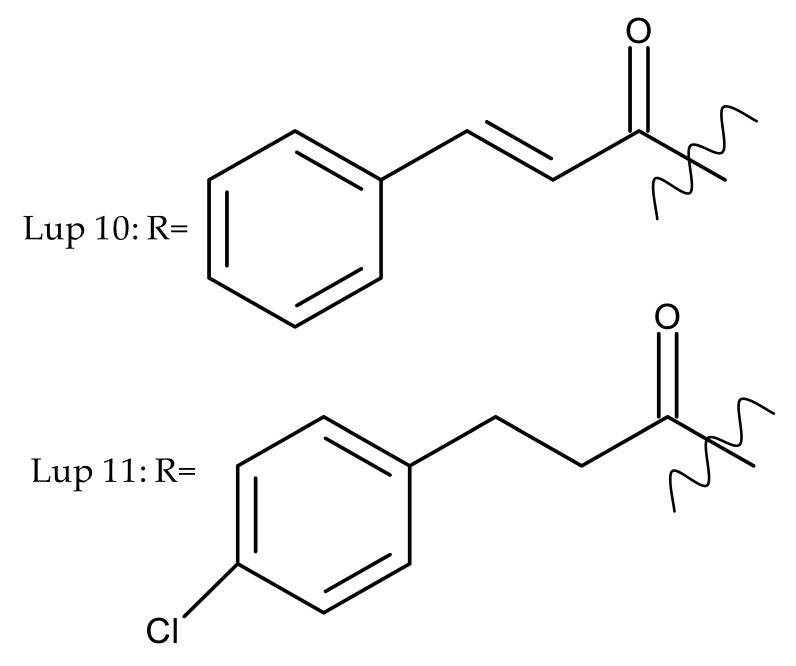	[16]
**Lup 12–13**	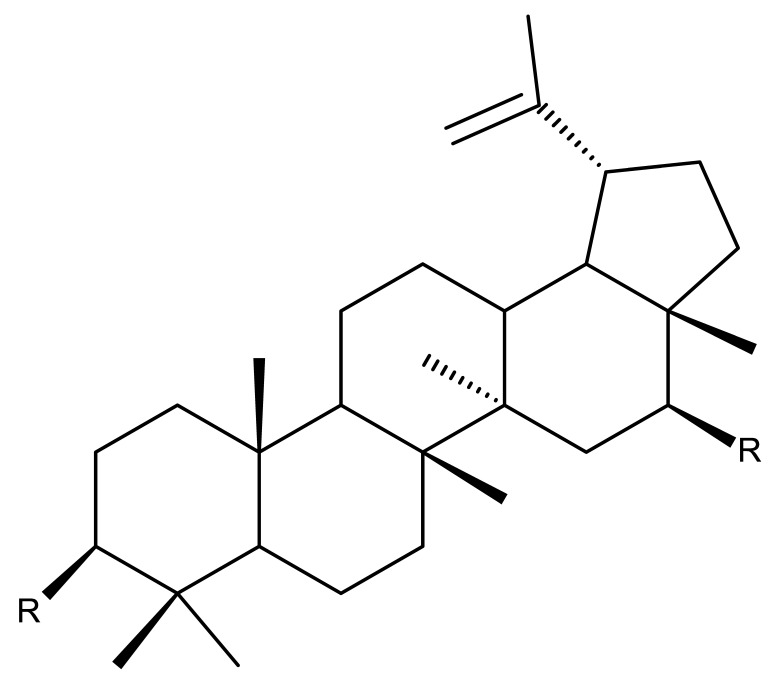	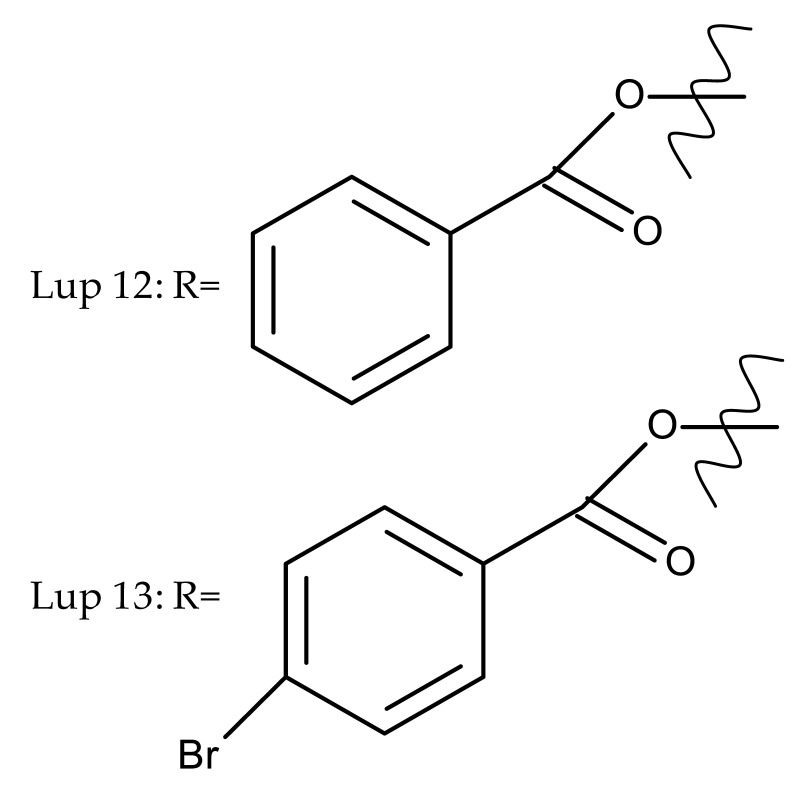	[18]

**Table 2:** The chemical structures of betulin derivatives.

Compound	Structure	Substituents	Reference
**Bet 1**	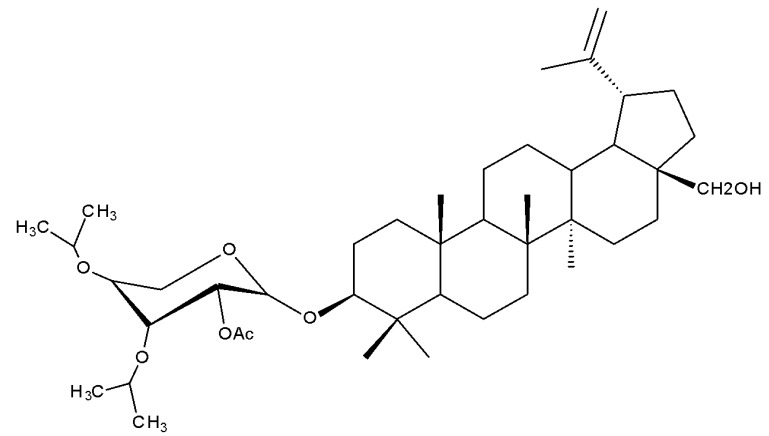	Ac: -C=O-CH_3_	[27]
**Bet 2–5**	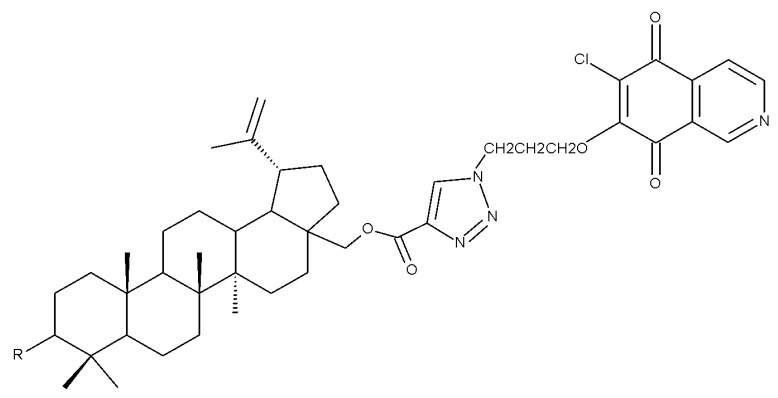	**Bet 2** R: -OH **Bet-3** R: =O **Bet-4** R: =OC(O)CH_3_ **Bet-5** R: =OC(O)CH_2_CH_3_	[28]
**Bet 6**	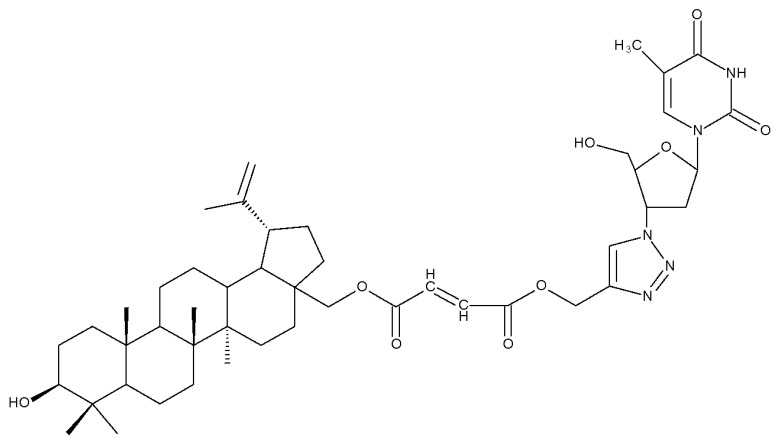		[29]
**Bet 7**	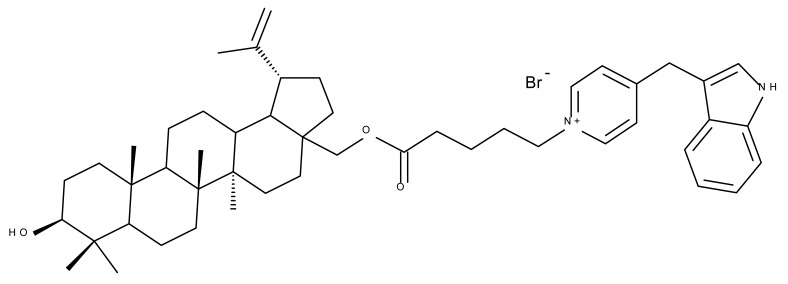		[30]
**Bet 8**	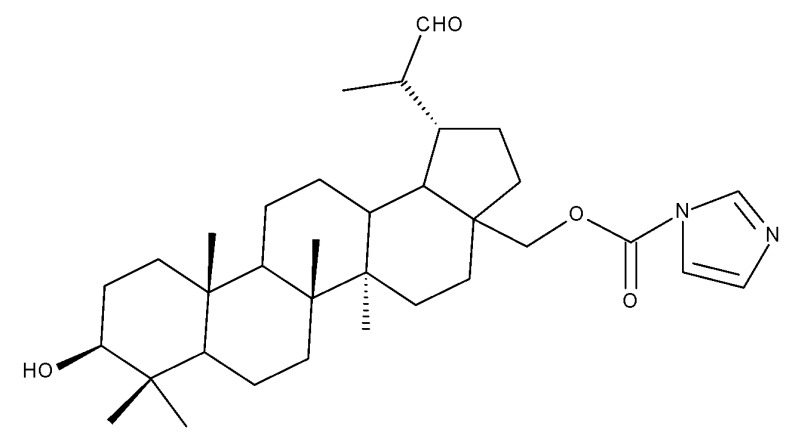		[31]
**Bet 9–10**	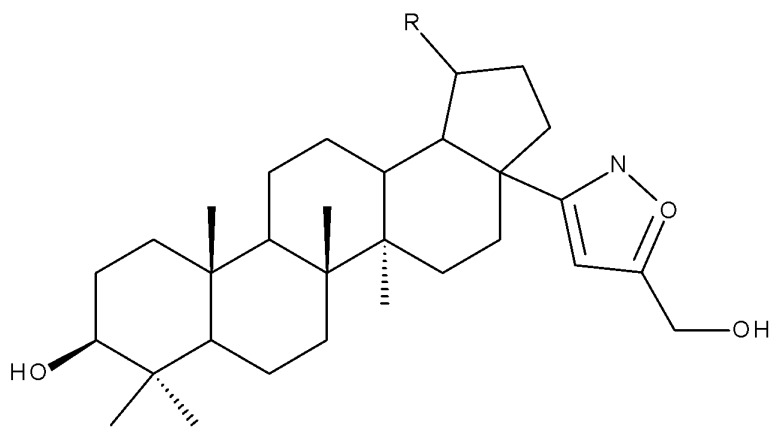	**Bet-9** R: 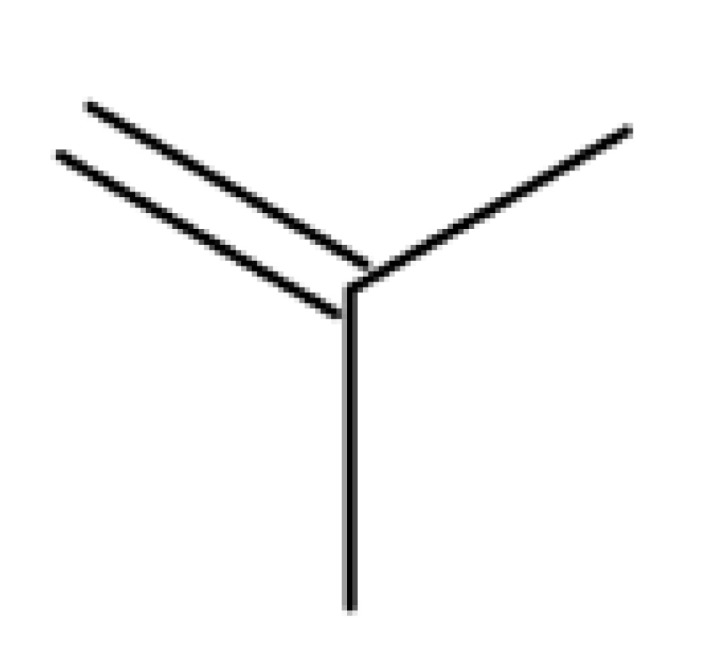 **Bet-10** R: 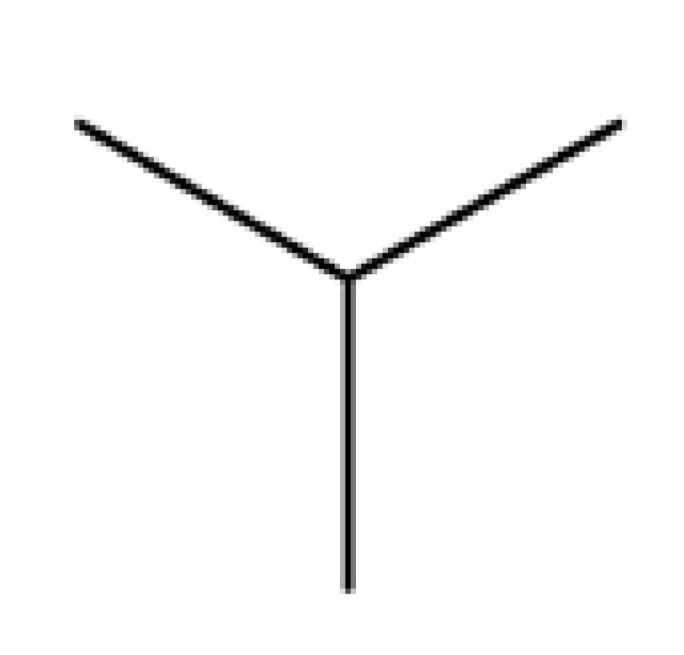	[32]
**Bet 11**	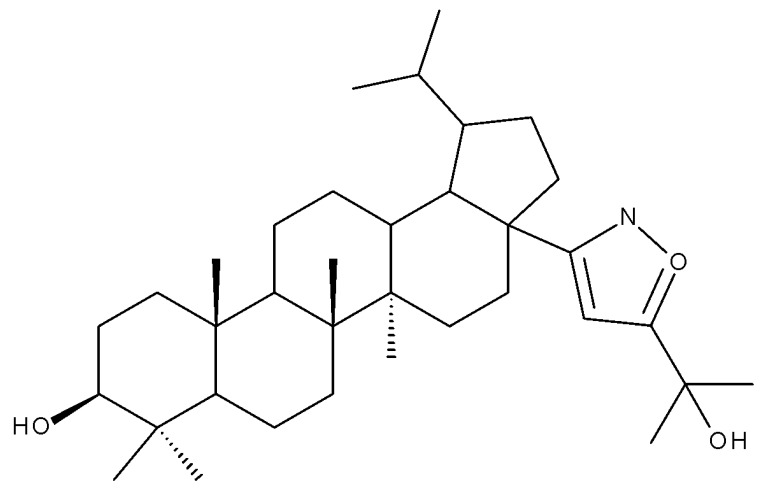		
**Bet 12**	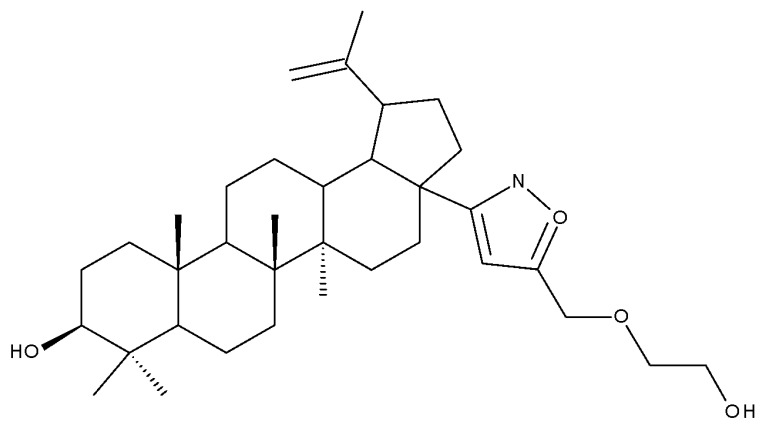		
**Bet 13**	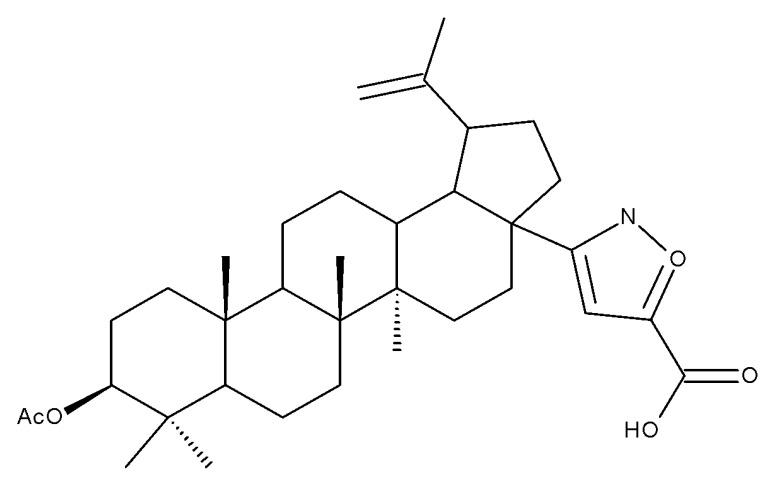	Ac: -C=O-CH_3_	
**Bet 14**	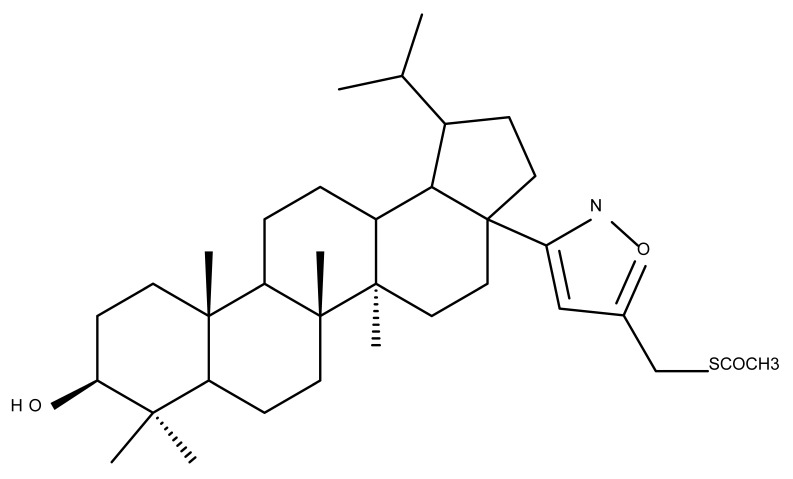		
**Bet 15**	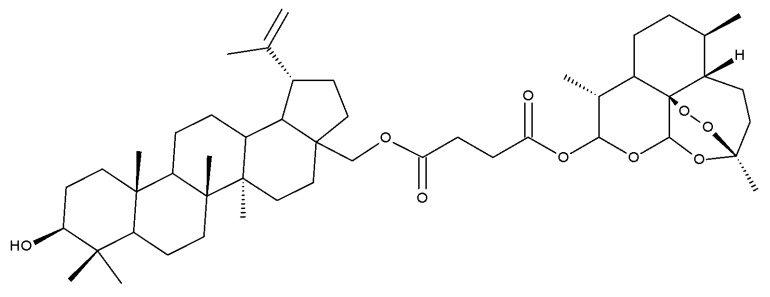		[33]
**Bet 16**	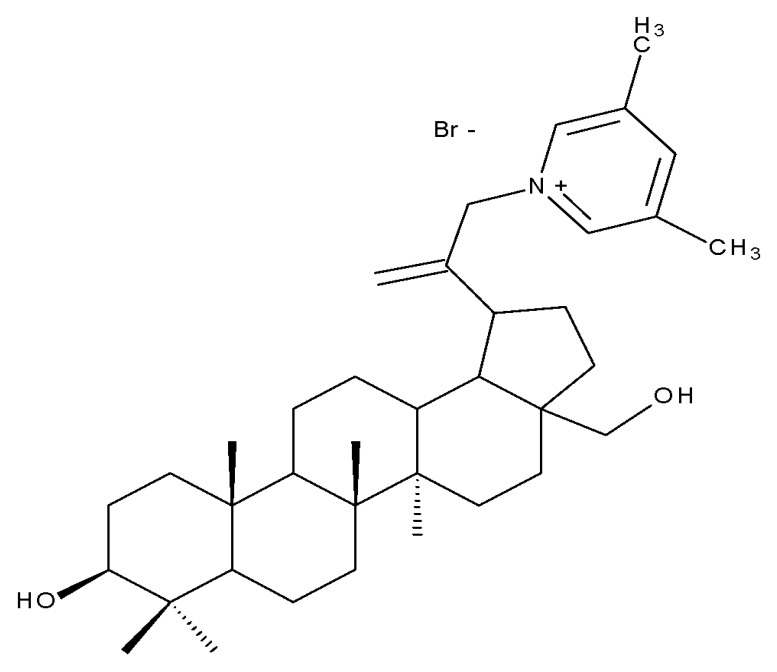		[35]
**Bet 17**	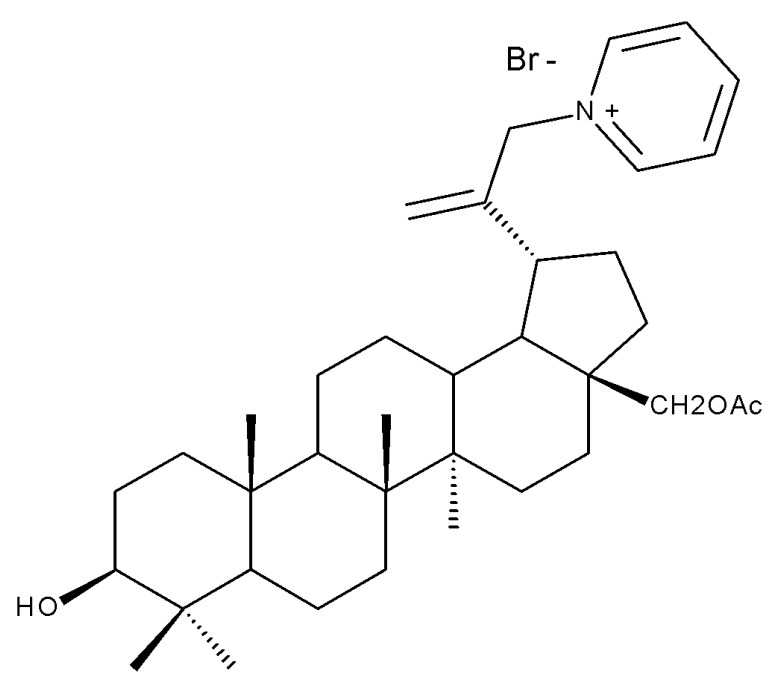	Ac: -C=O-CH_3_	[36]
**Bet 18–20**	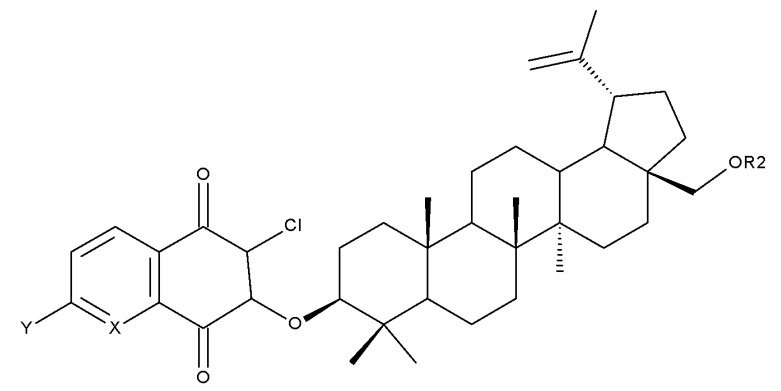	**Bet 18** R2: -C(O)C≡CH Y:H, X:N **Bet 19** R2: =C(O)CH3 Y:H, X: CH **Bet 20** R2: -C(O)C≡CH Y:H, X:CH	[37]
**Bet 21**	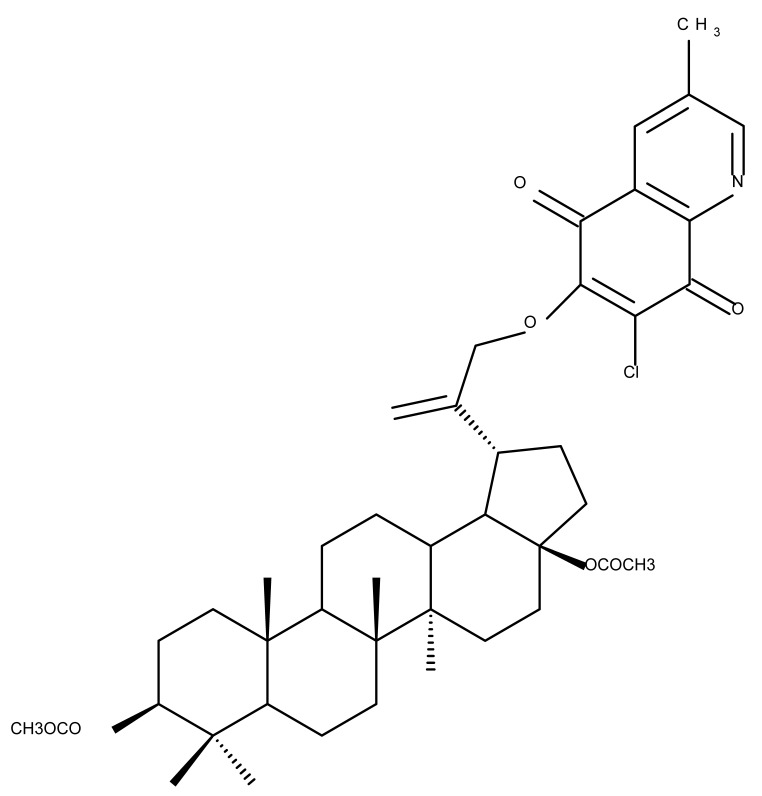		
**Bet 22**	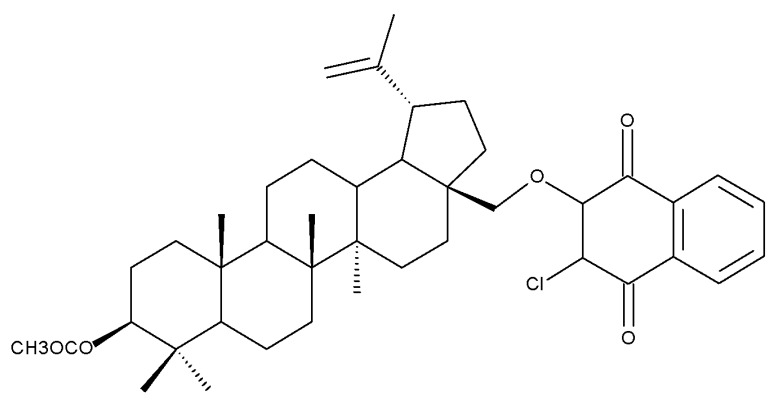		
**Bet 23**	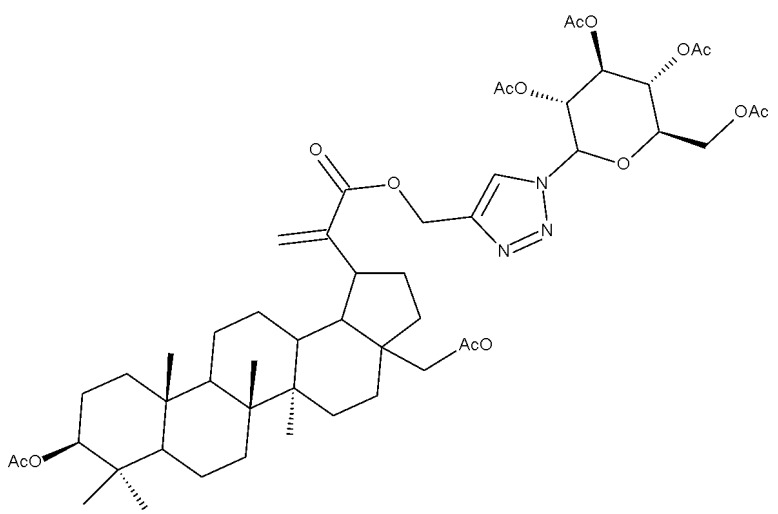	Ac: -C=O-CH_3_	[38]
**Bet 24**	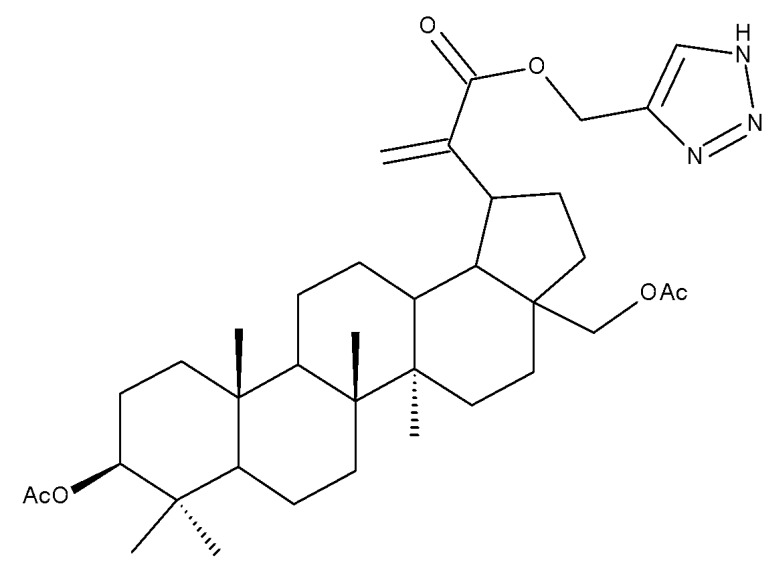		
**Bet 25**	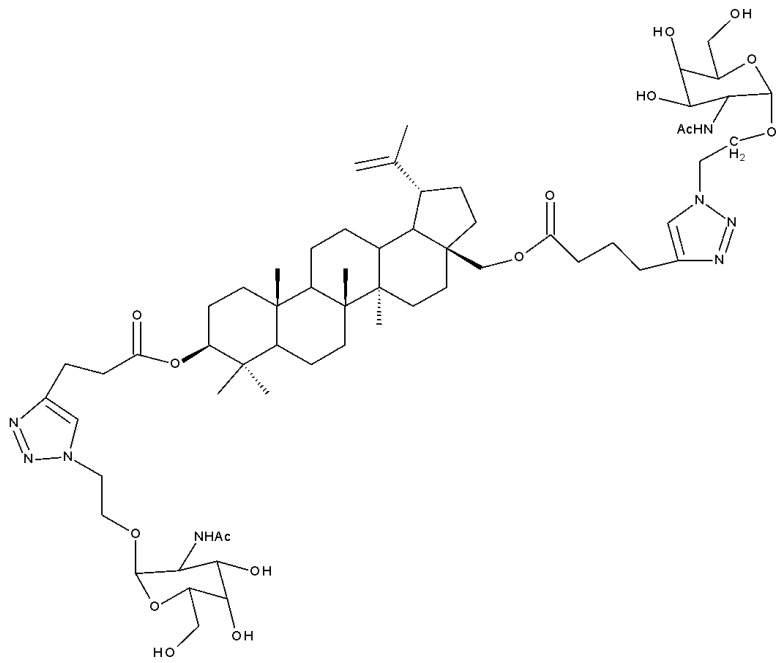	Ac: -C=O-CH_3_	[39]
**Bet 26**	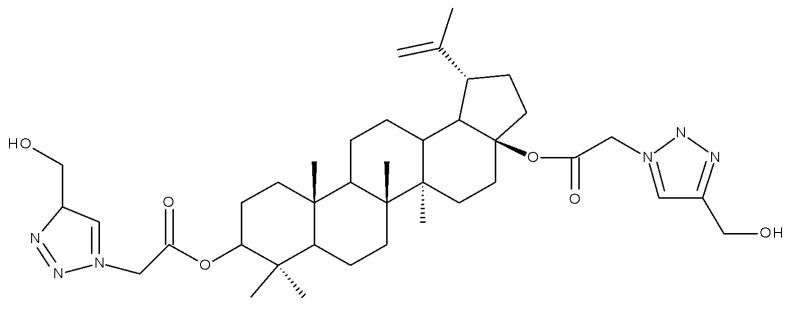		[40]
**Bet 27**	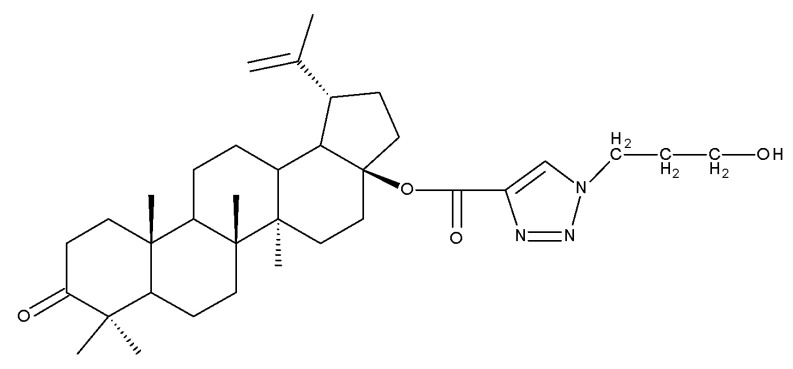		[41]
**Bet 28**	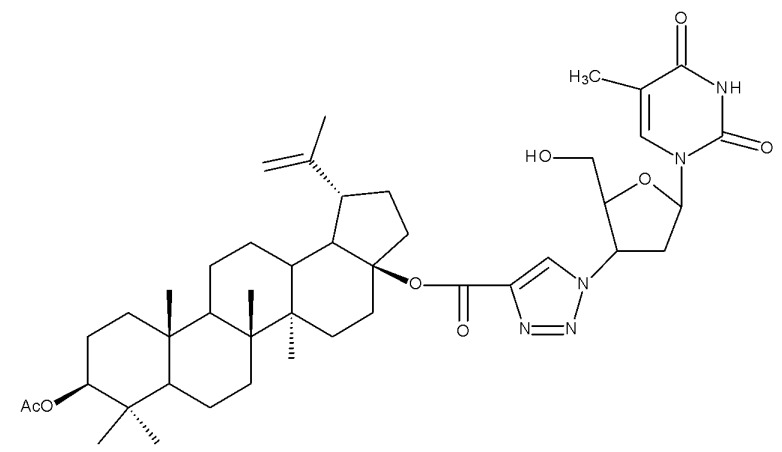	Ac: -C=O-CH_3_	
**Bet 29**	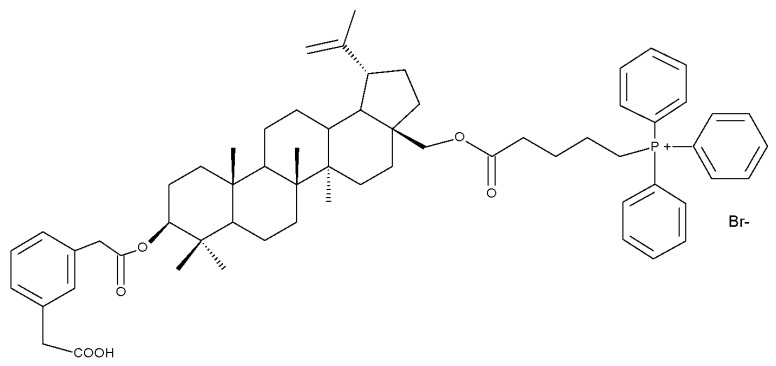		[42]
**Bet 30**	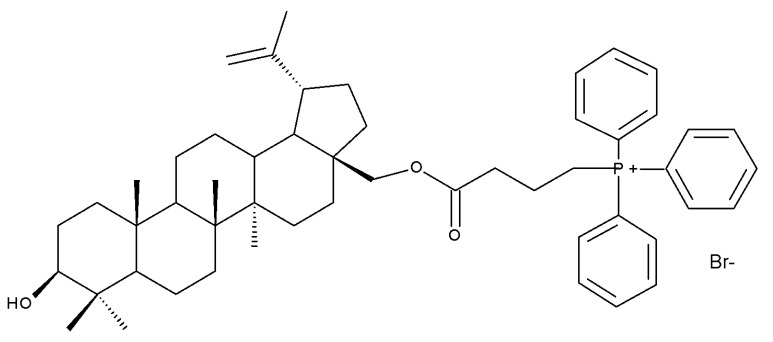		[43]
**Bet 31**	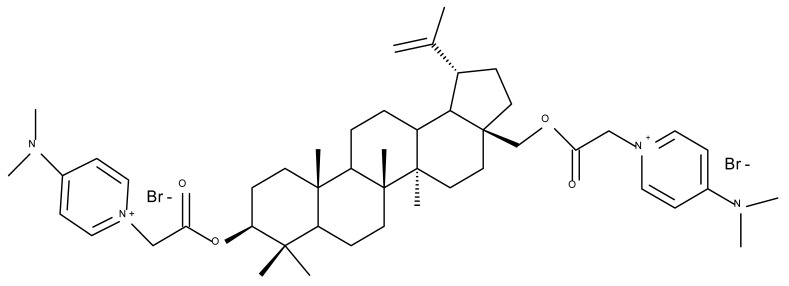		[34]
**Bet 32**	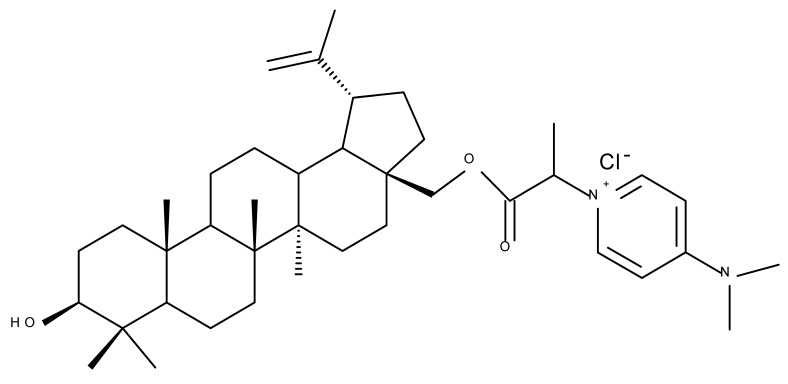		
**Bet 33**	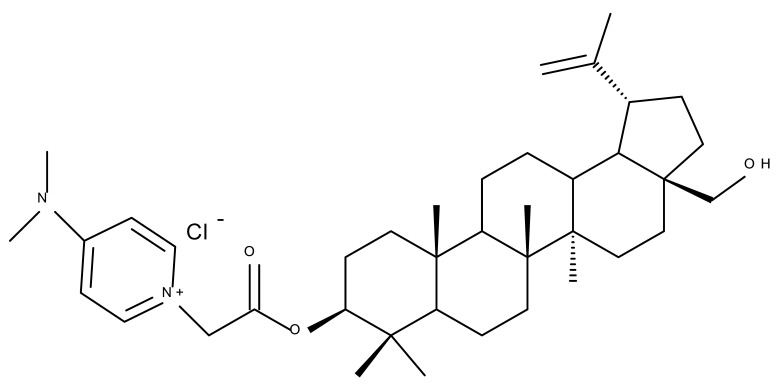		
**Bet 34–36**	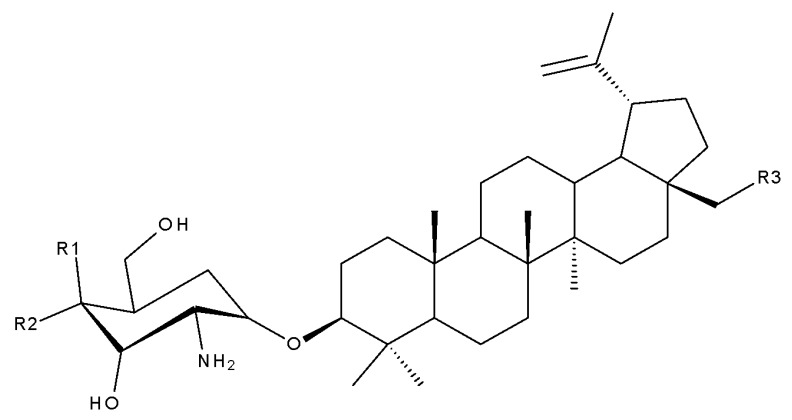	**Bet 36** R1: -H, R2: -OH, R3: -OCOCH_3_ **Bet 37** R1: -H R2 = R3: -OH **Bet 38** R1: -OH R2: -H R3: -OH	[46]
**Bet 37**	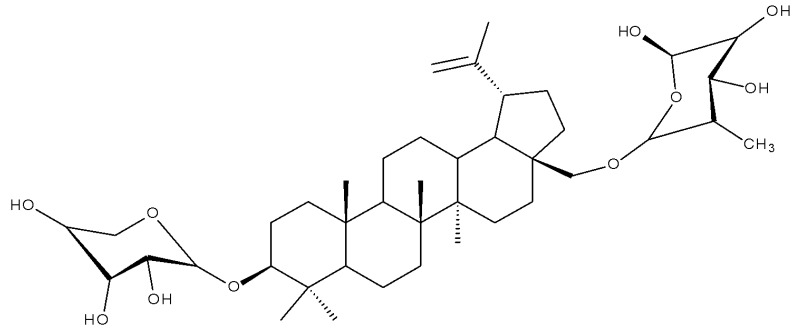		[47]
**Bet 38**	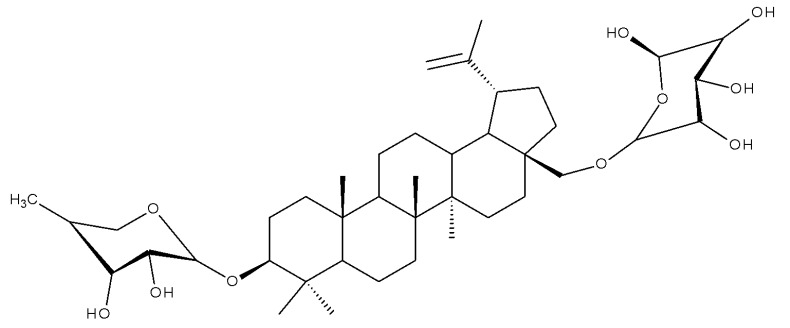		
**Bet 39**	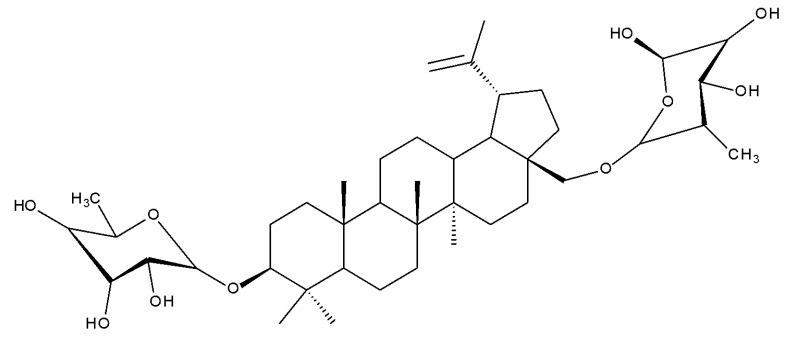		[48]
**Bet 40**	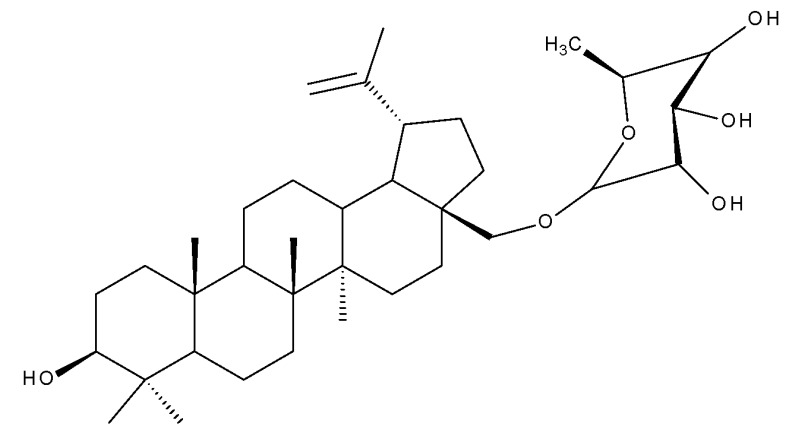		[49]
**Bet 41**	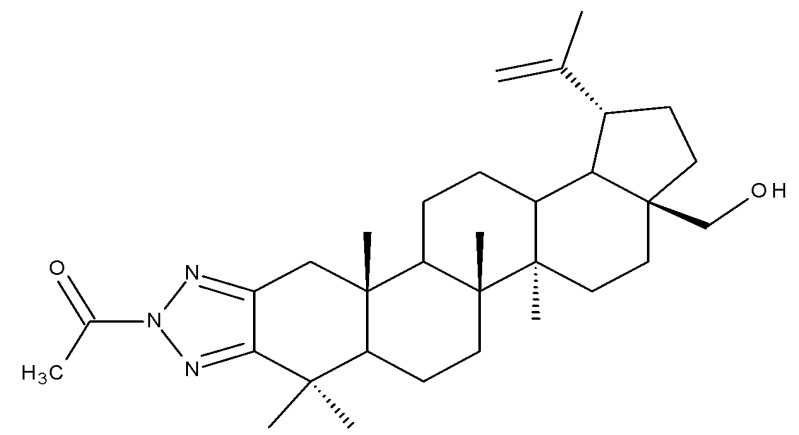		[50]
**Bet 42**	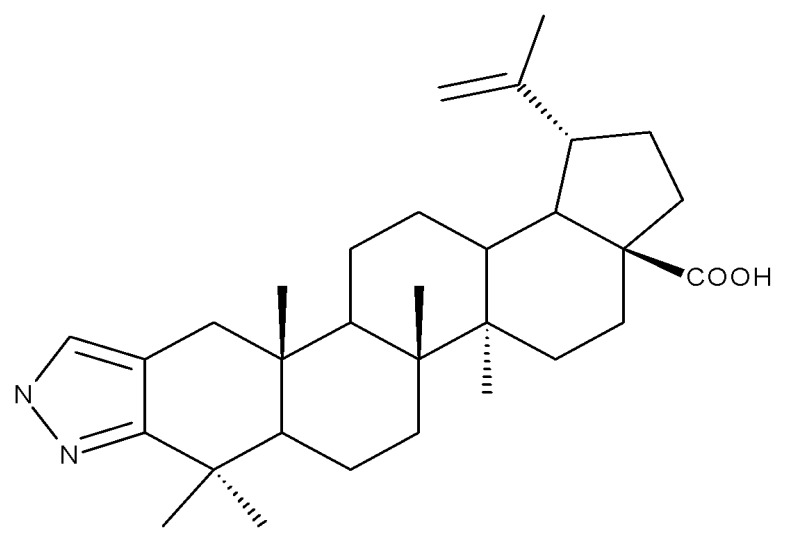		[51]

**Table 3:** In vitro cytotoxicity of BA-aminopropyl and BA-piperazinyl derivatives (IC_50_; µM).

Cell Line	Type of Cells Cancer/Non-Cancer	BA and BA Derivatives				
		BA	Beviramat	BA 27	BA 28	BA 29
CCRF-CEM	Leukemia	>50	12.82	0.29	1.55	2.92
CEM-DNR	Leukemia	23.05	22.17	0.35	11.53	7.00
K562	Leukemia	>50	23.60	0.40	5.25	23.19
K562-Tax	Leukemia	>50	22.03	0.52	31.80	12.21
A549	Lung adenocarcinoma	22.68	23.06	1.26	6.65	13.55
HCT116	Colorectal adenocarcinoma	>50	14.17	0.39	3.85	7.92
HCT116P53−/−	Colorectal adenocarcinoma	>50	18.20	0.44	3.39	8.80
U2OS	Osteosarcoma	29.69	27.63	0.42	5.00	12.38
MRC-5	Human fibroblasts	>50	>50	1.58	8.07	14.12
BJ	Human fibroblasts	>50	>50	1.59	8.37	15.49

**Table 4:** Chemical structures of BA derivatives.

**Compound**	**Structure**	**Substituents**	**Reference**
**BA 1**	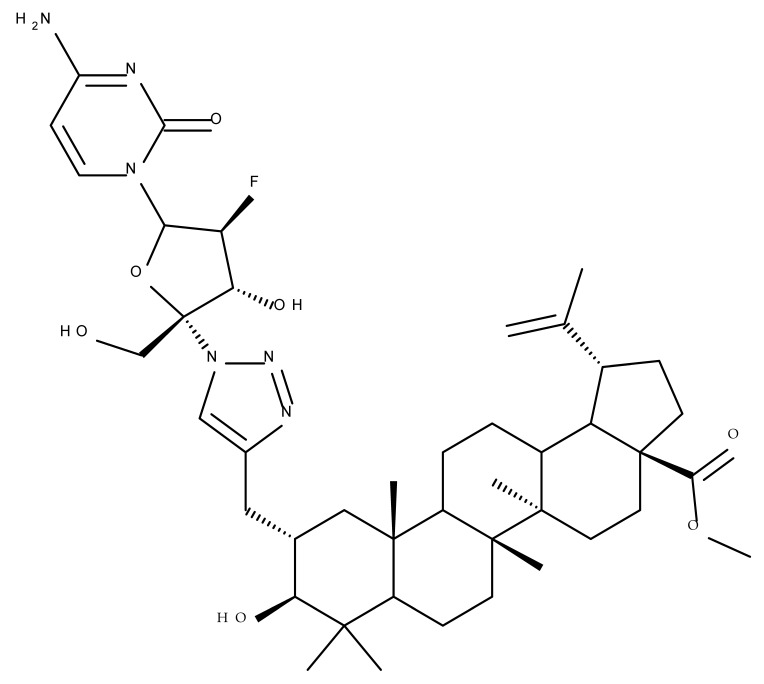	-	[56]
**BA 2–3**	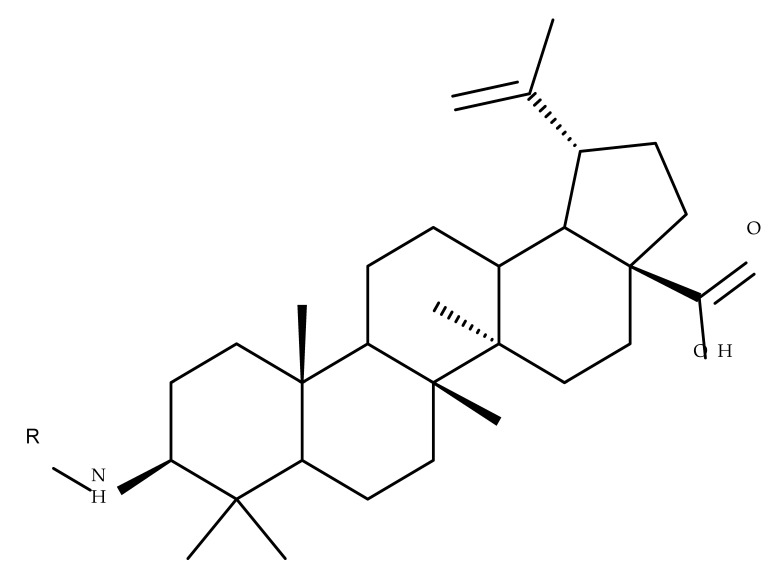	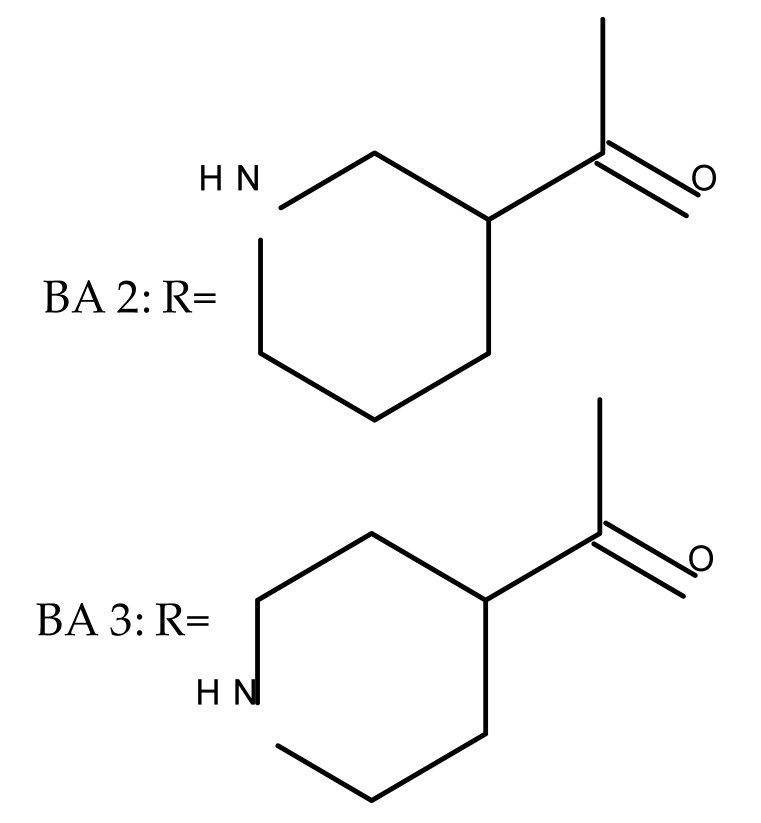	[57]
**BA 4–6**	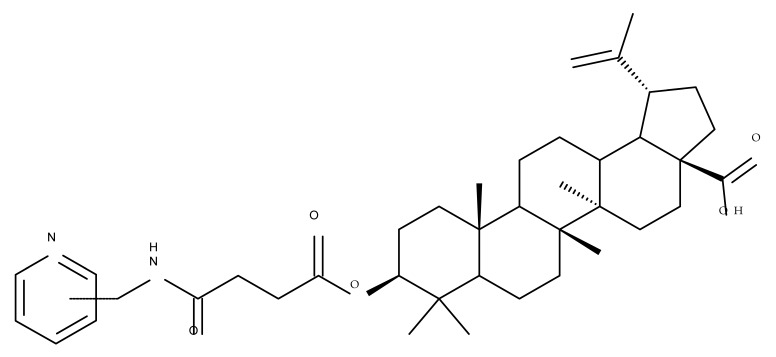	-	[23]
**BA 7–9**	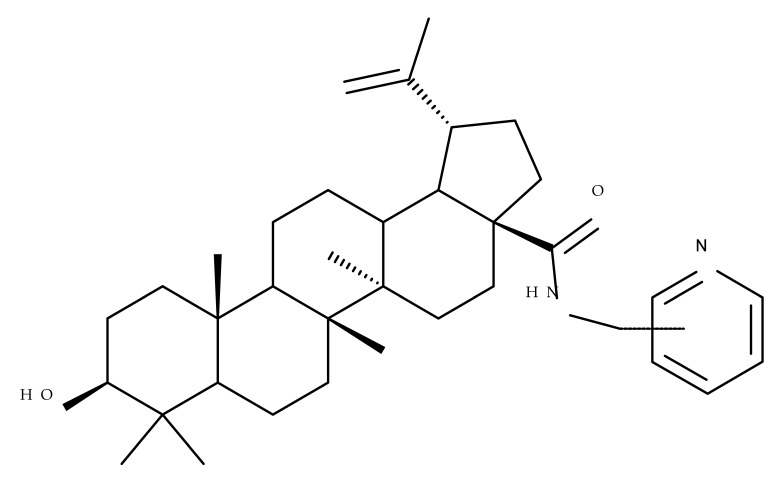	-	[23]
**BA 10**	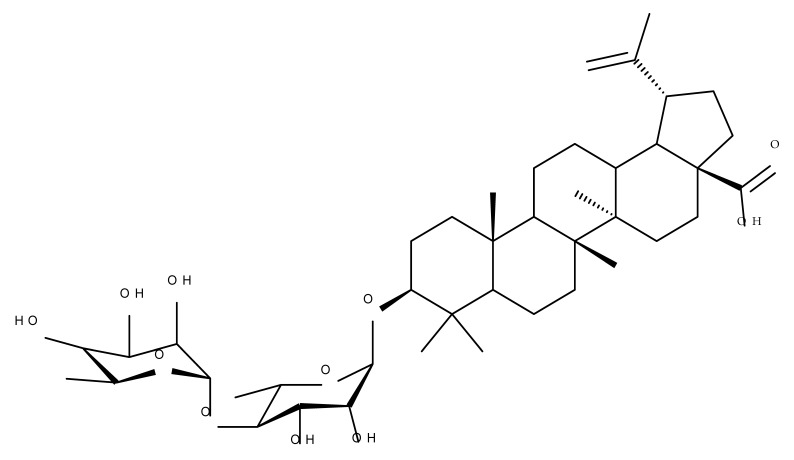	-	[59]
**BA 11–12**	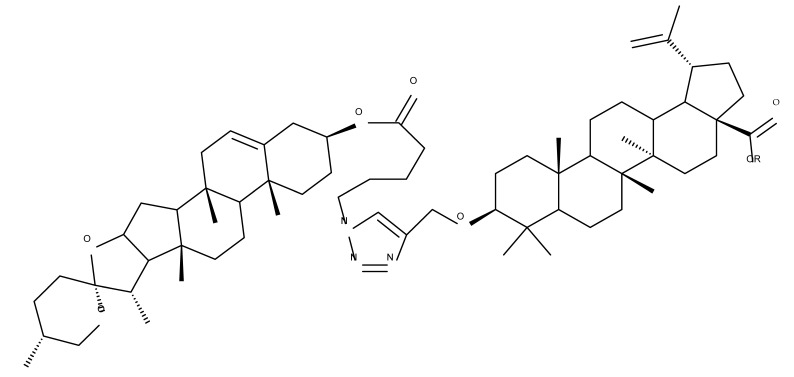	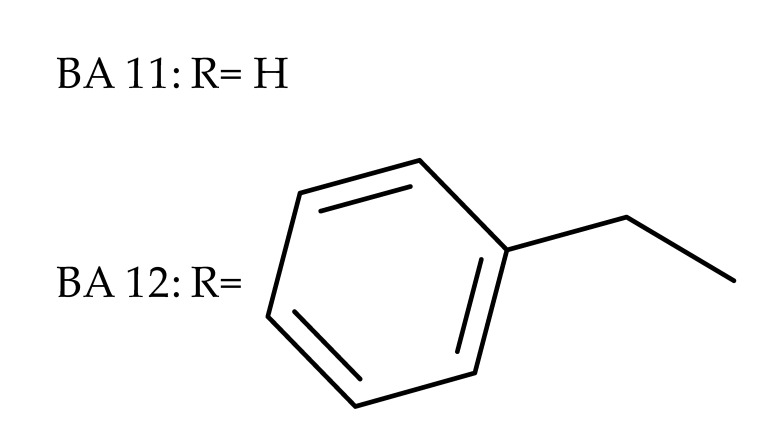	[60]
**BA 13**	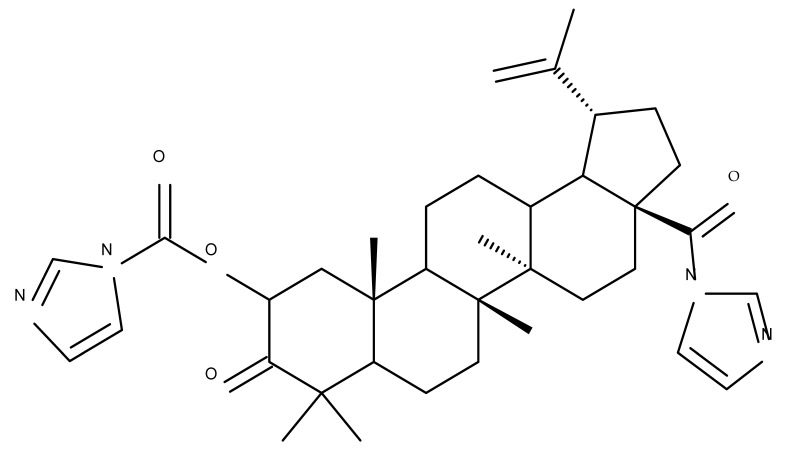	-	[31]
**BA 14–21**	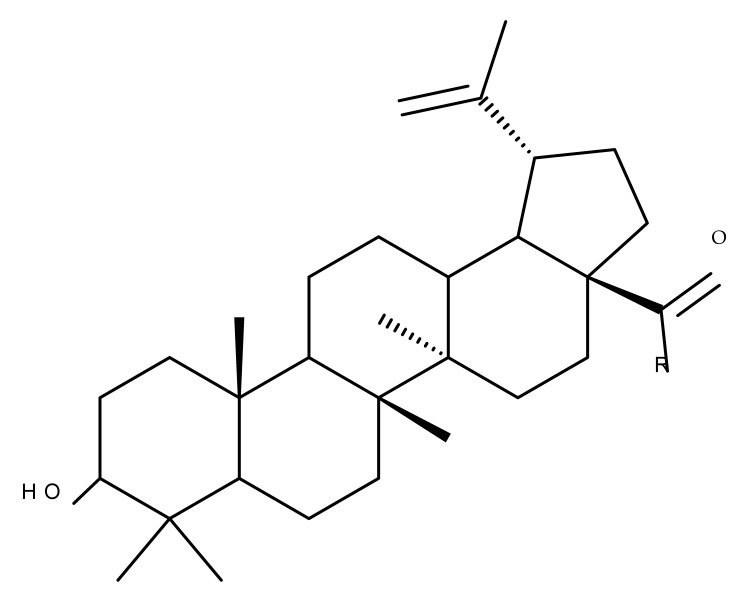	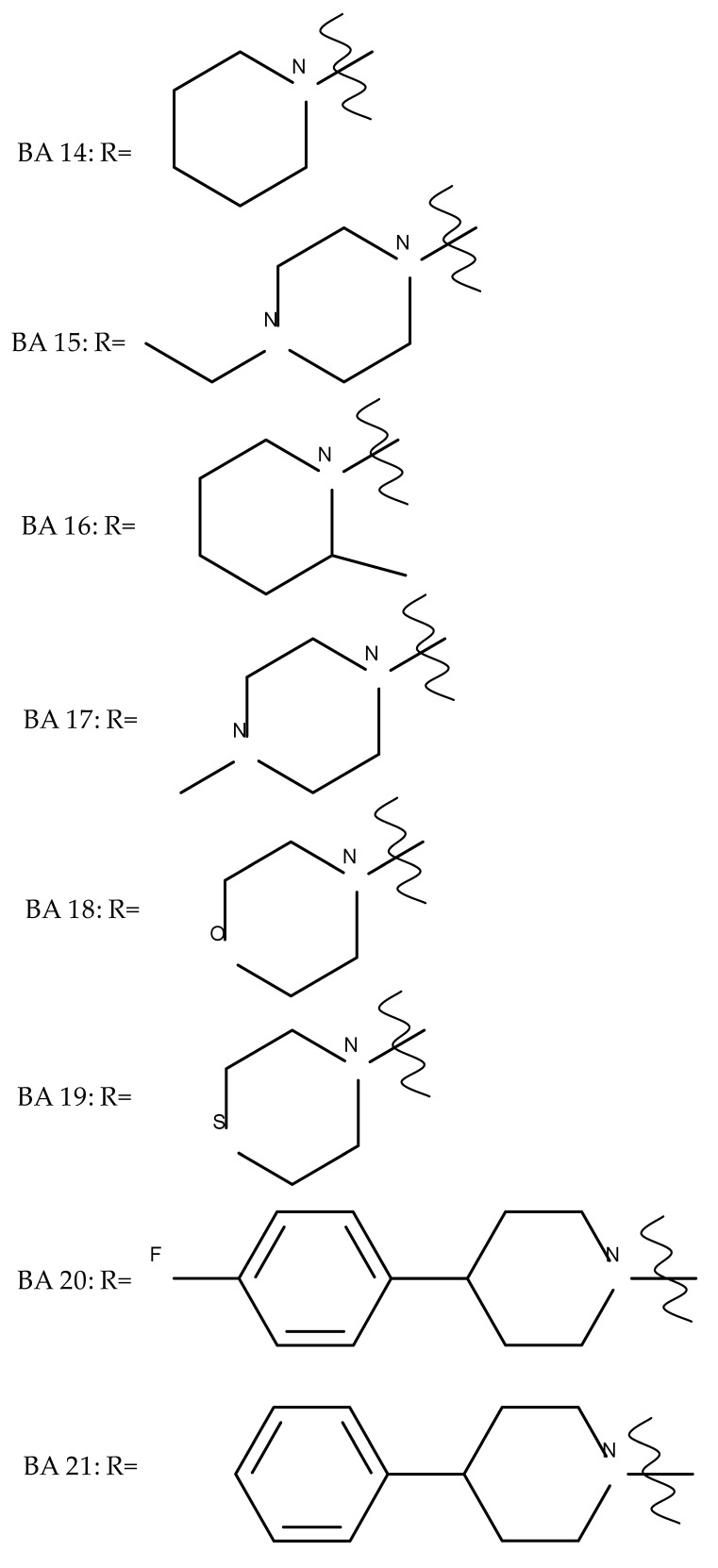	[61]
**BA 22**	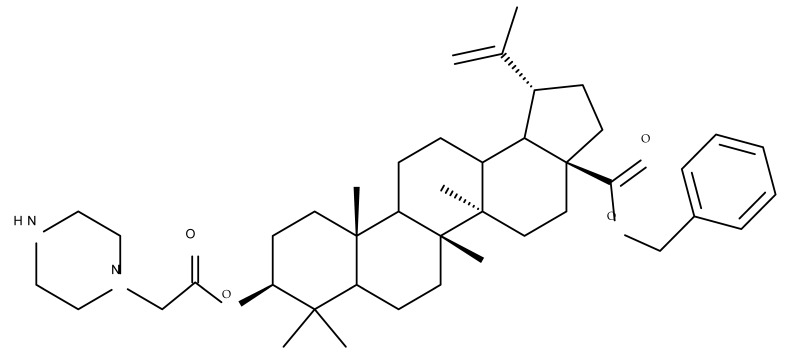	-	[63]
**BA 23–25**	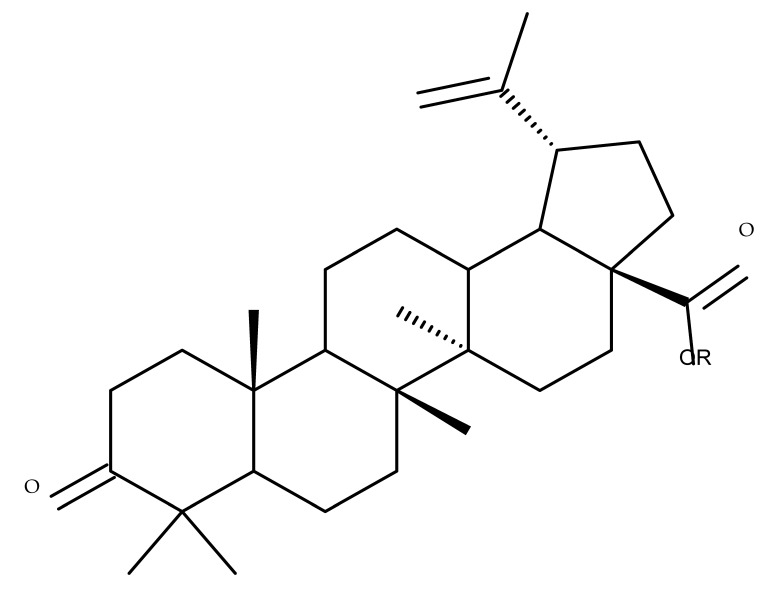	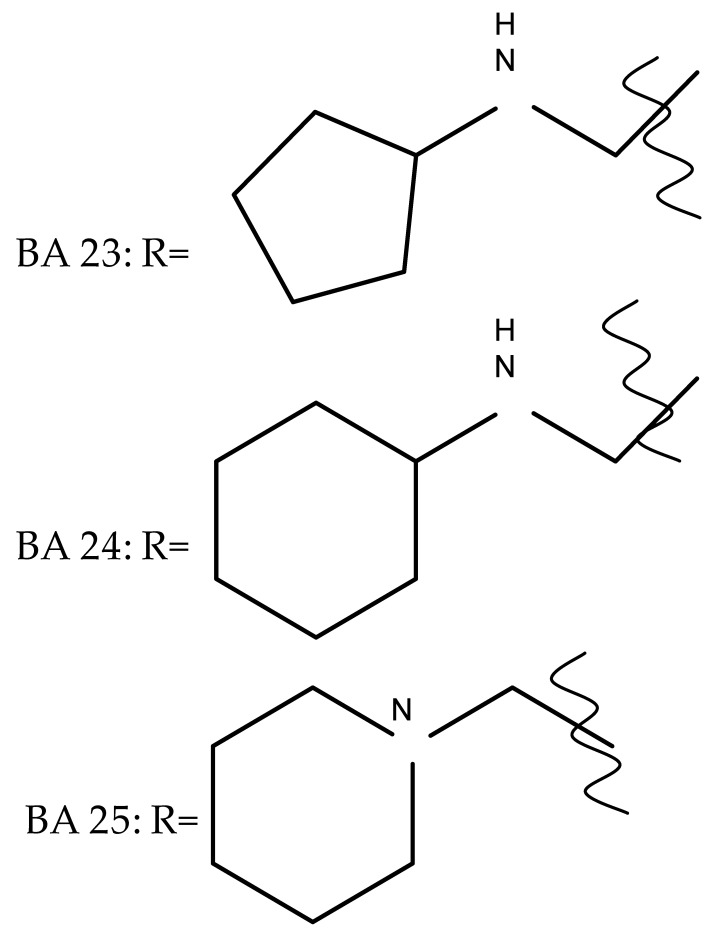	[63]
**BA 26**	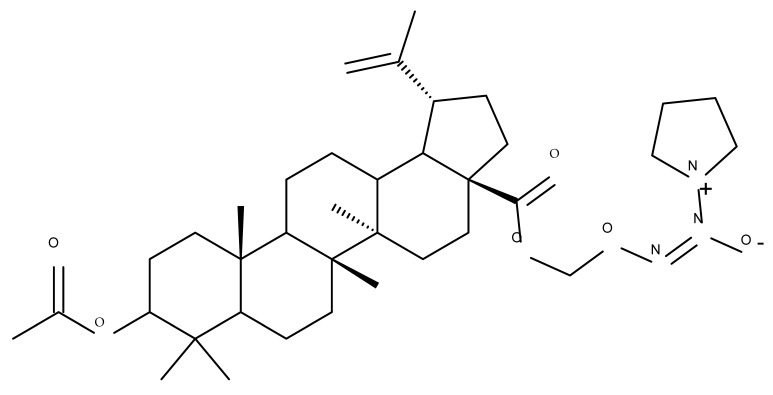		[64]
**Beviramat**	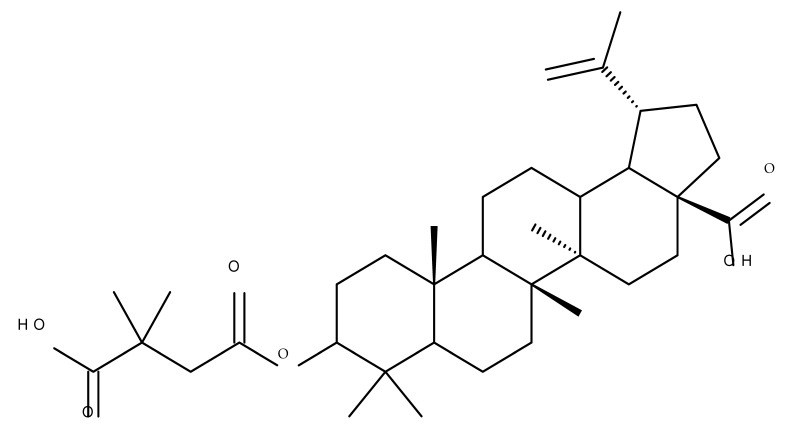		[65]
**BA 27**	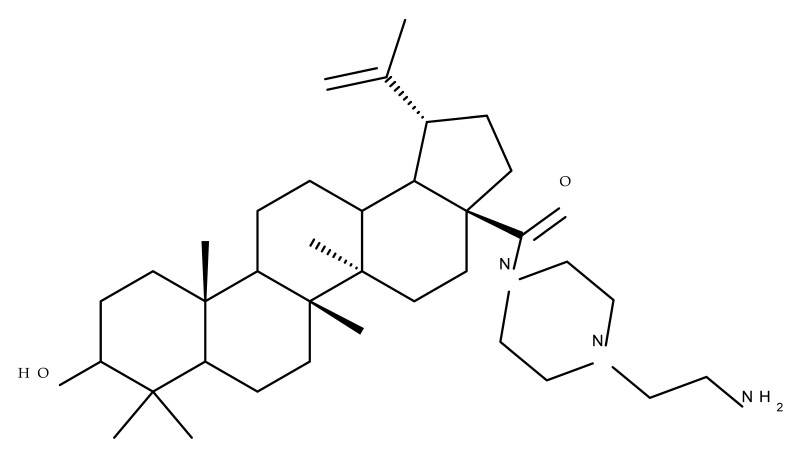		[65]
**BA 28**	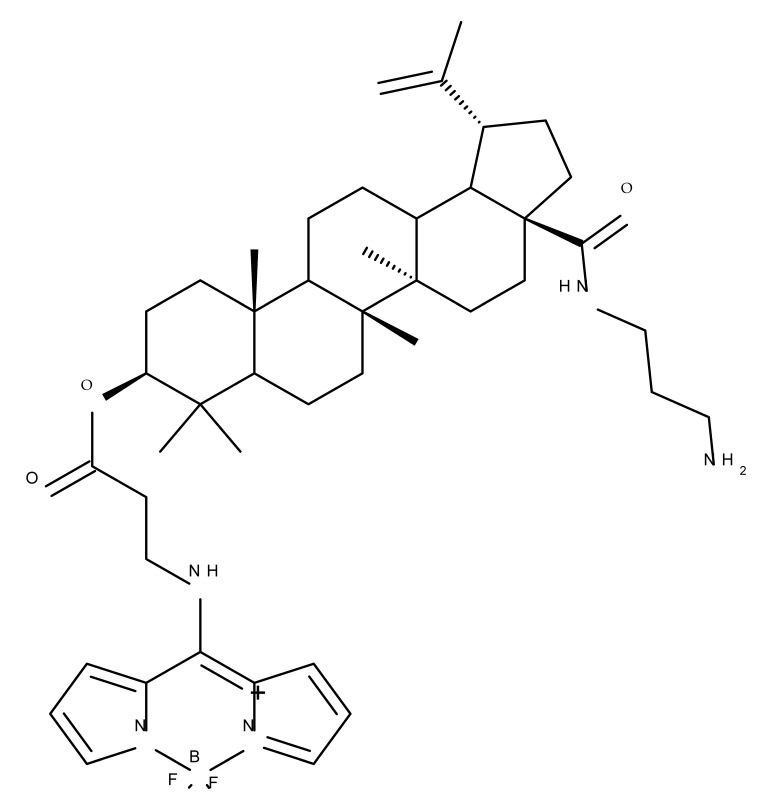		[65]
**BA 29**	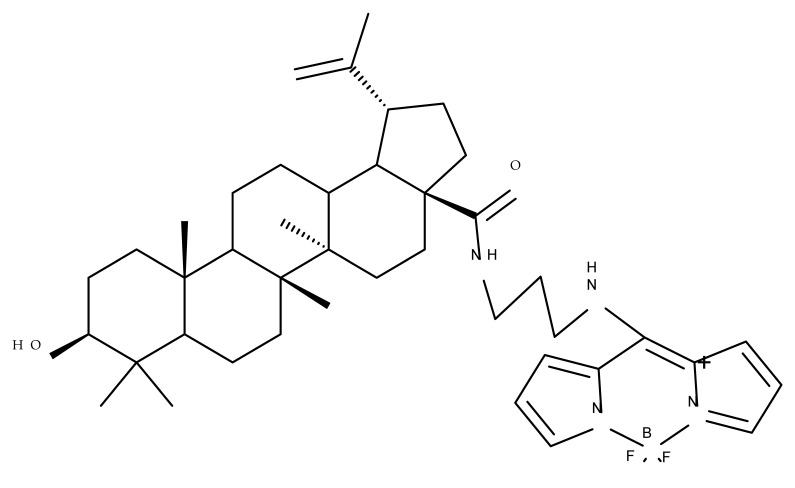		[65]
**BA 30**	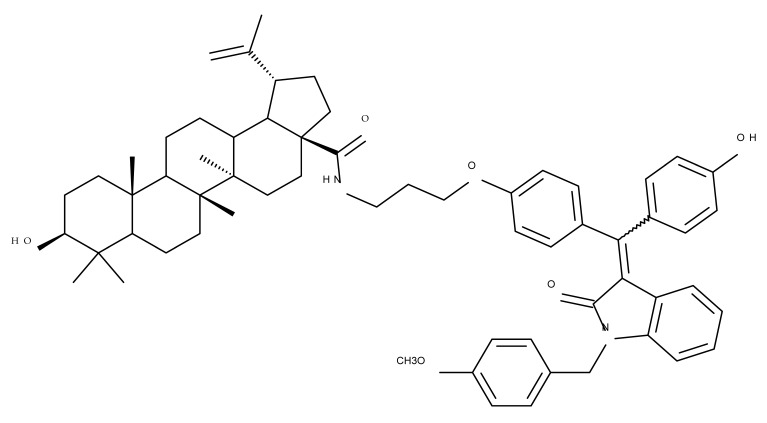		[67]
**BA 31–32**	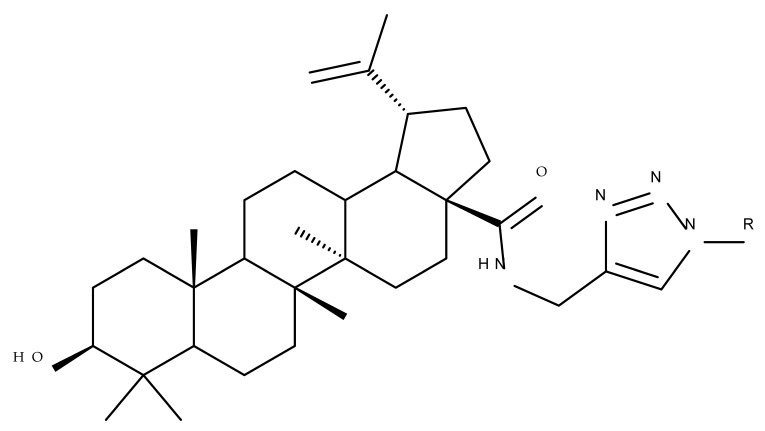	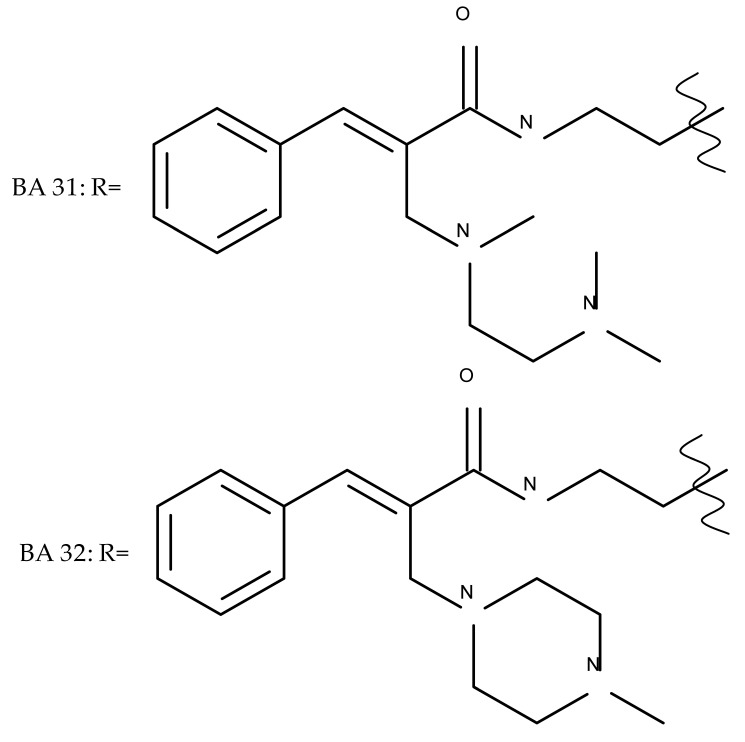	[68]
**BA 33–34**	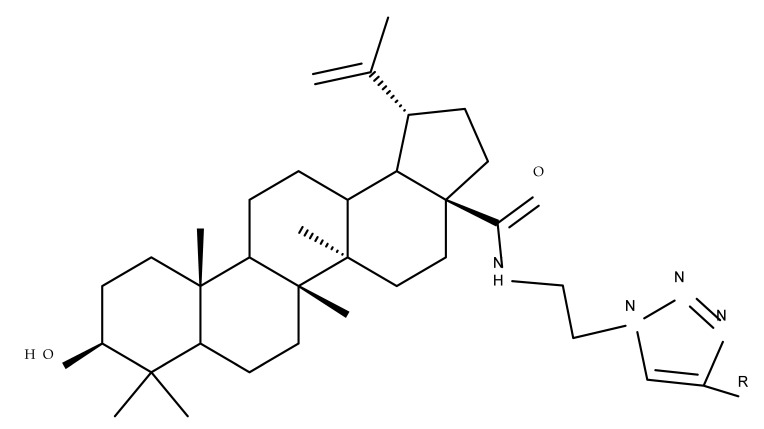	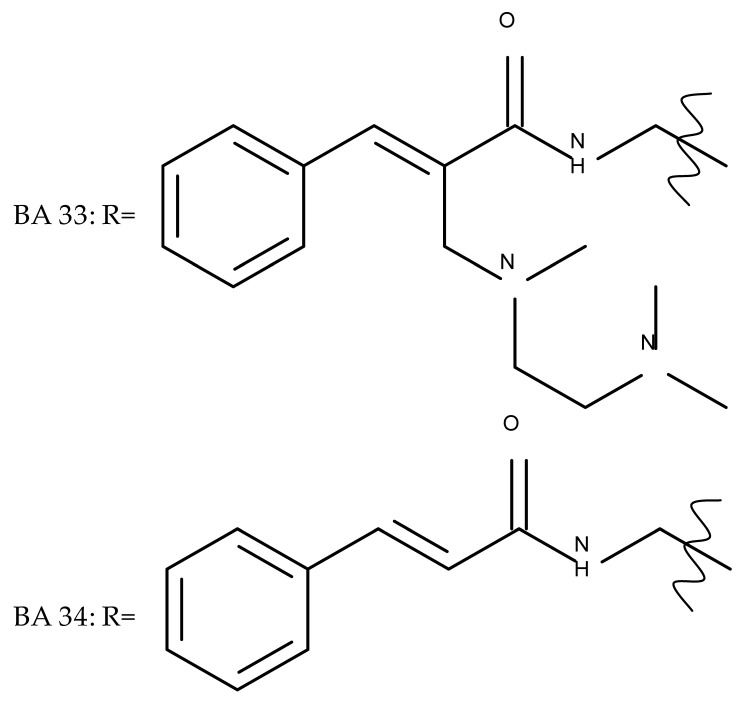	[68]
**BA 35**	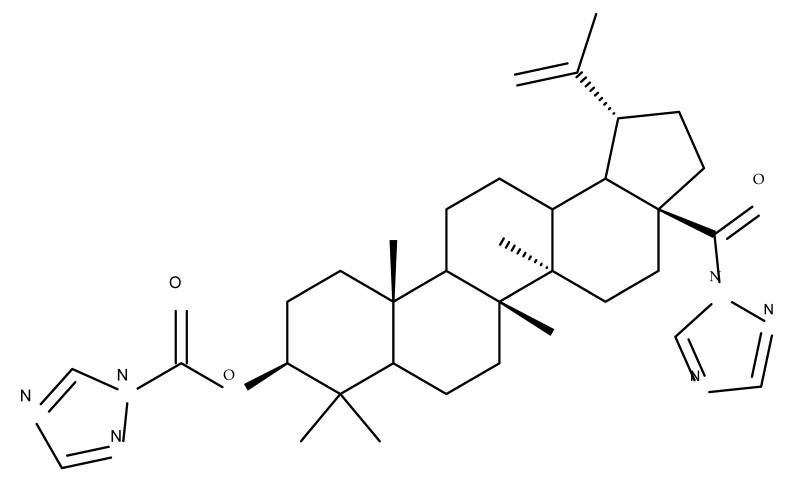	-	[69]
**BA 36**	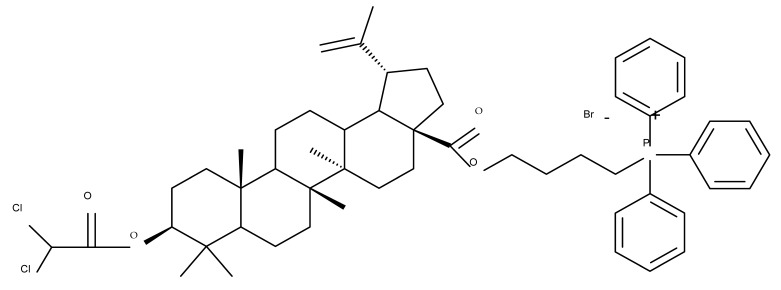	-	[70]
**BA 37–40**	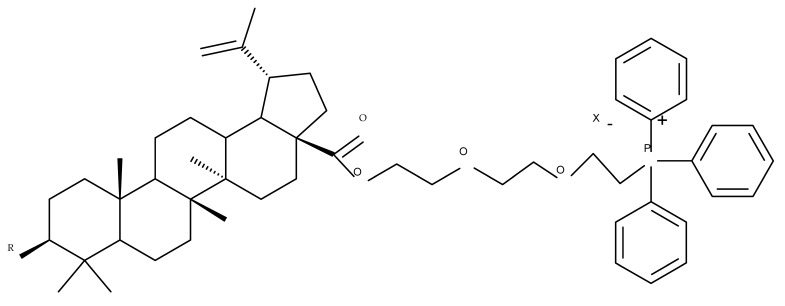	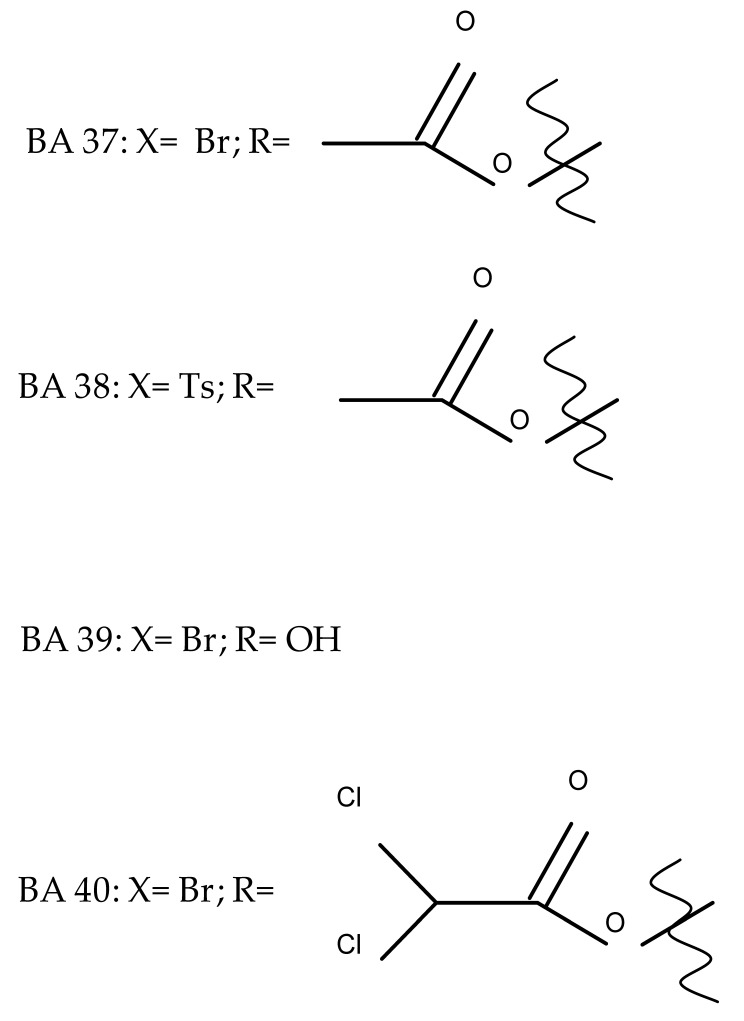	[70]
**BA 41–42**	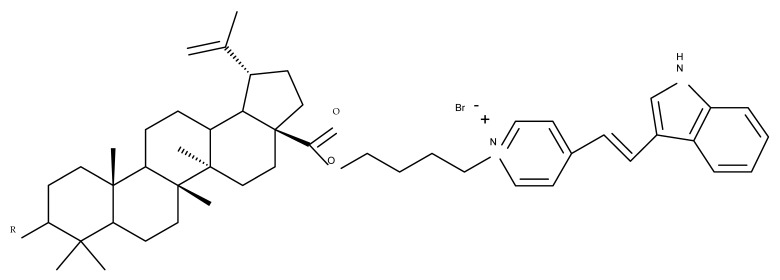	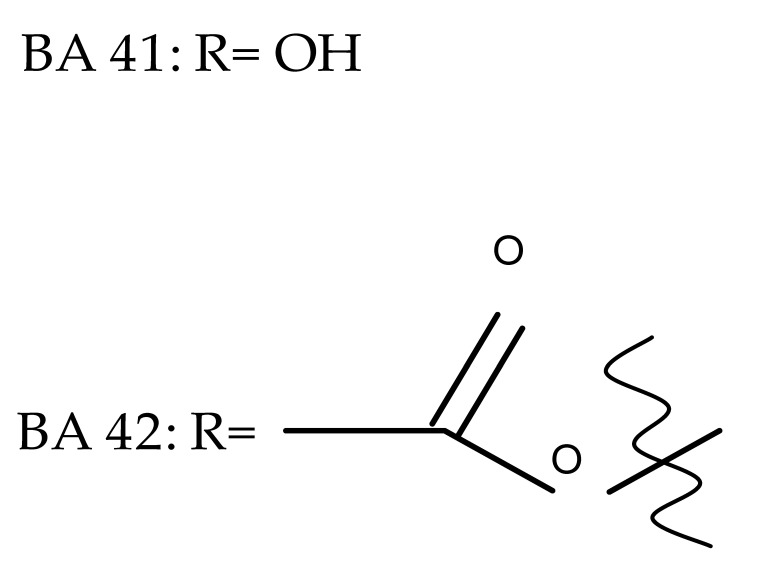	[73]
**BA 43**	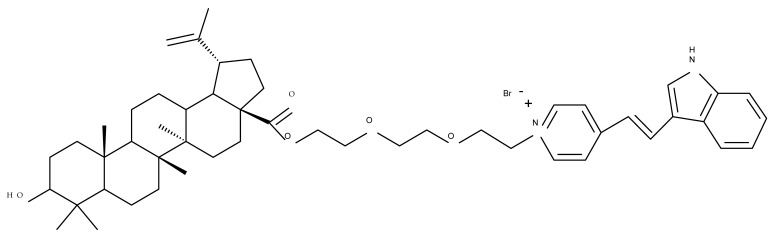		[73]
**BA 44**	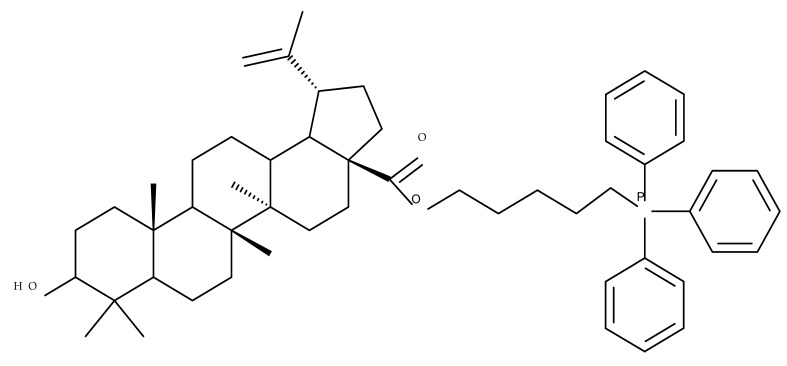	-	[74]
**BA 45**	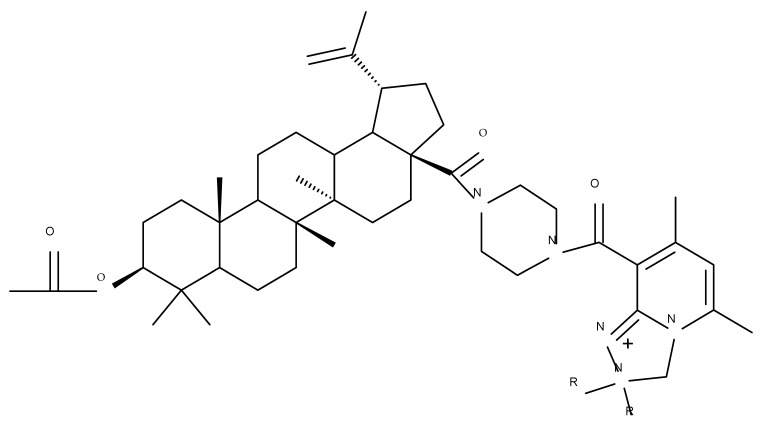	R = hexyl	[75]
**BA 46**	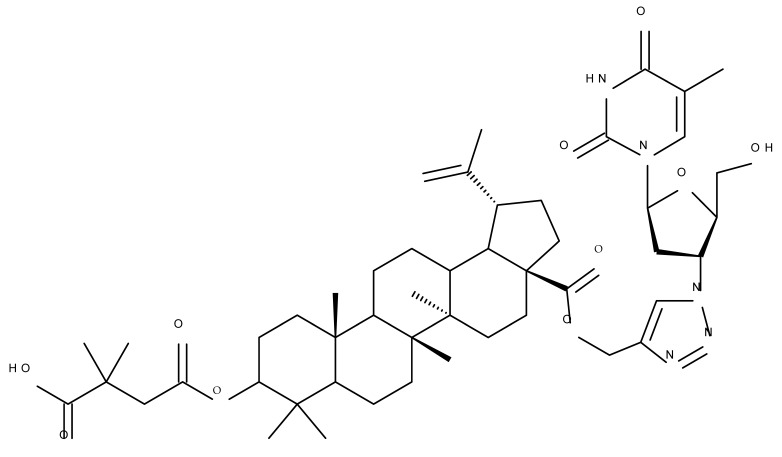		[76]
**BA 47**	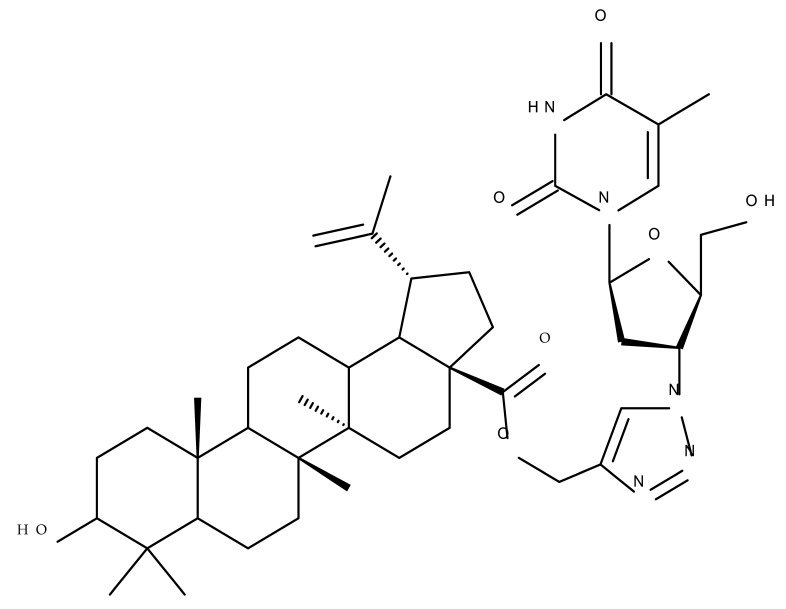		[77]
**BA 48–49**	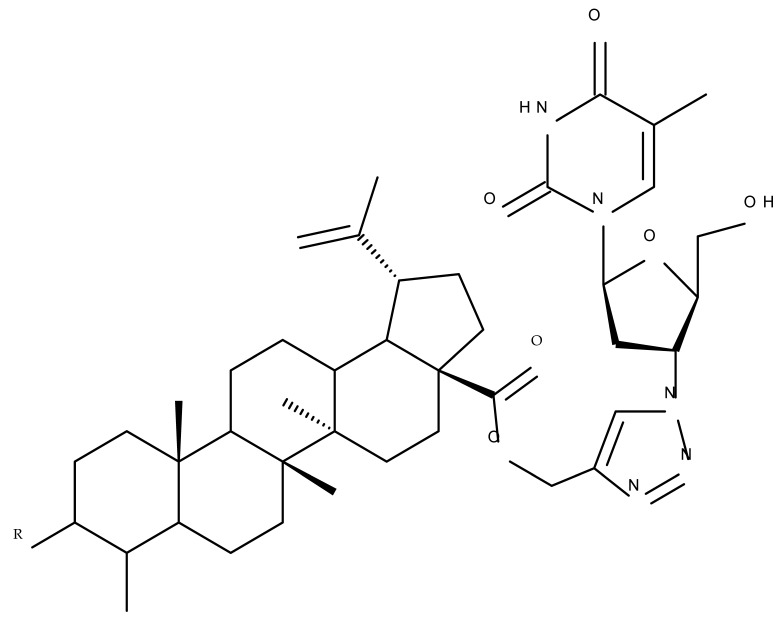	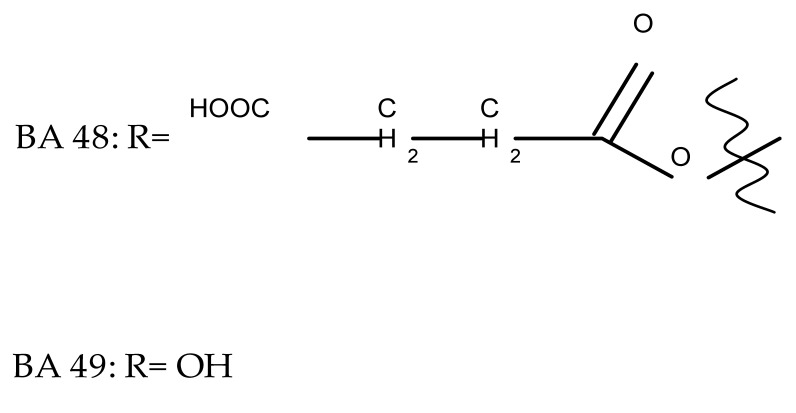	[77]
**BA 50**	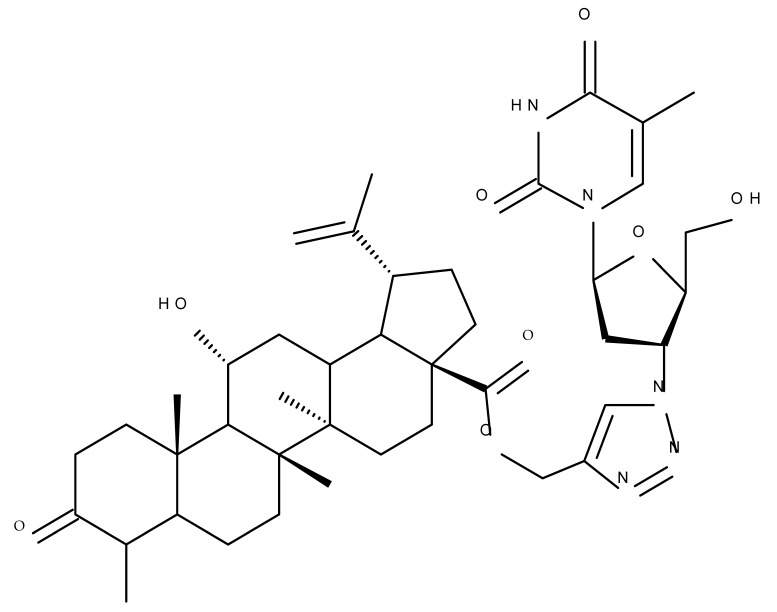	-	[77]
**BA 51**	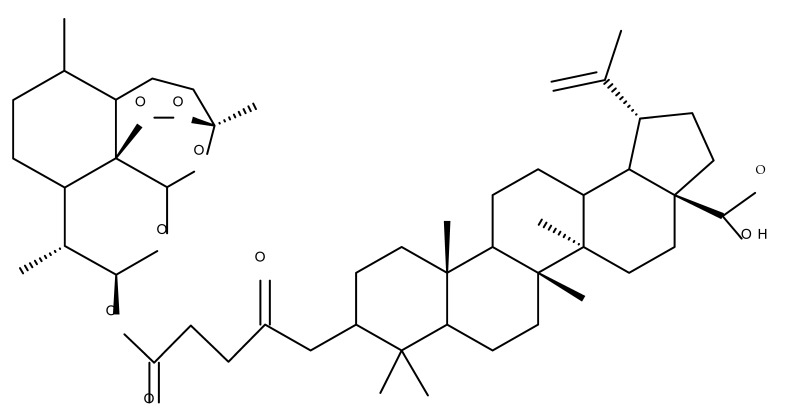	-	[33]
**BA 52**	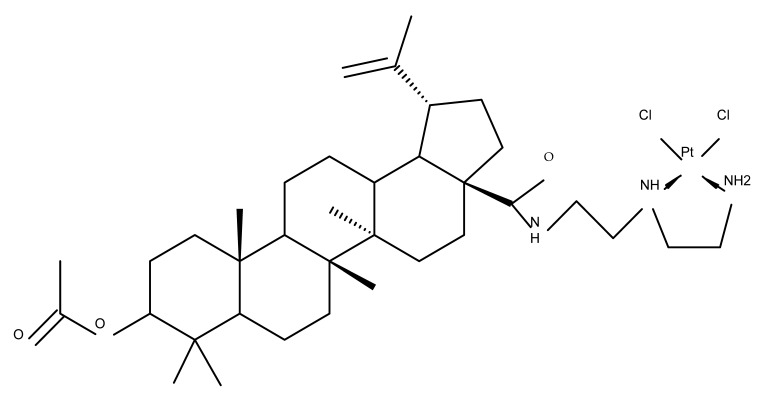		[82]
**BA 53**	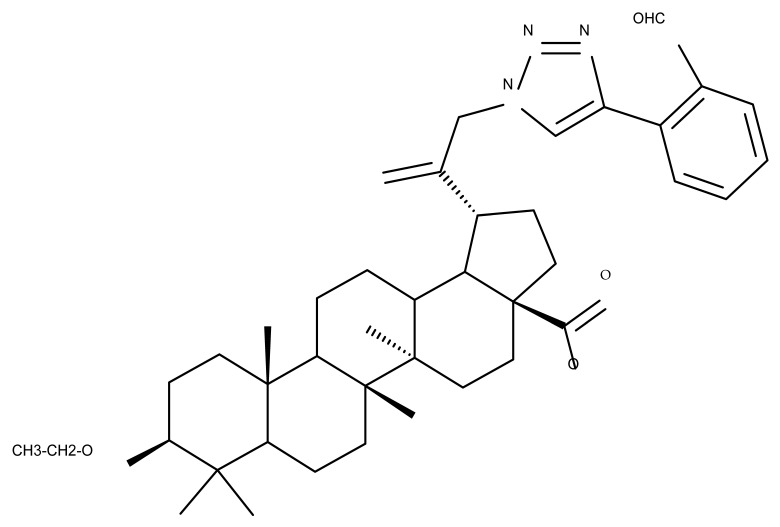	-	[91]
**BA 54–55**	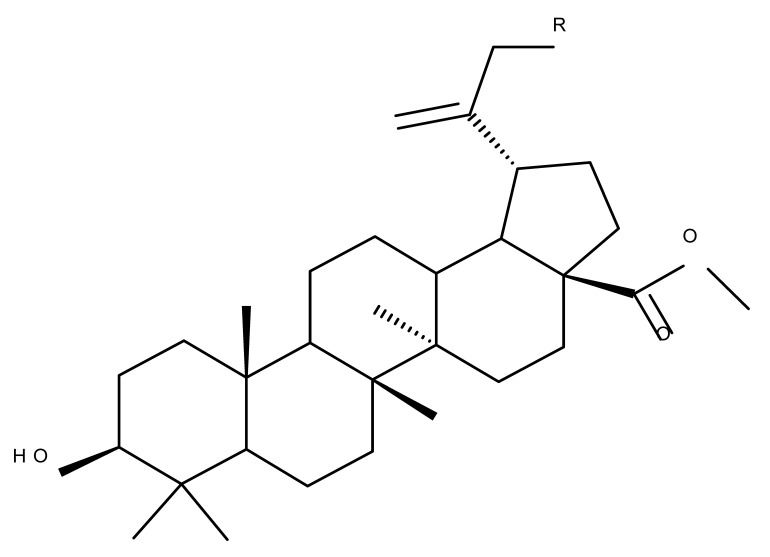	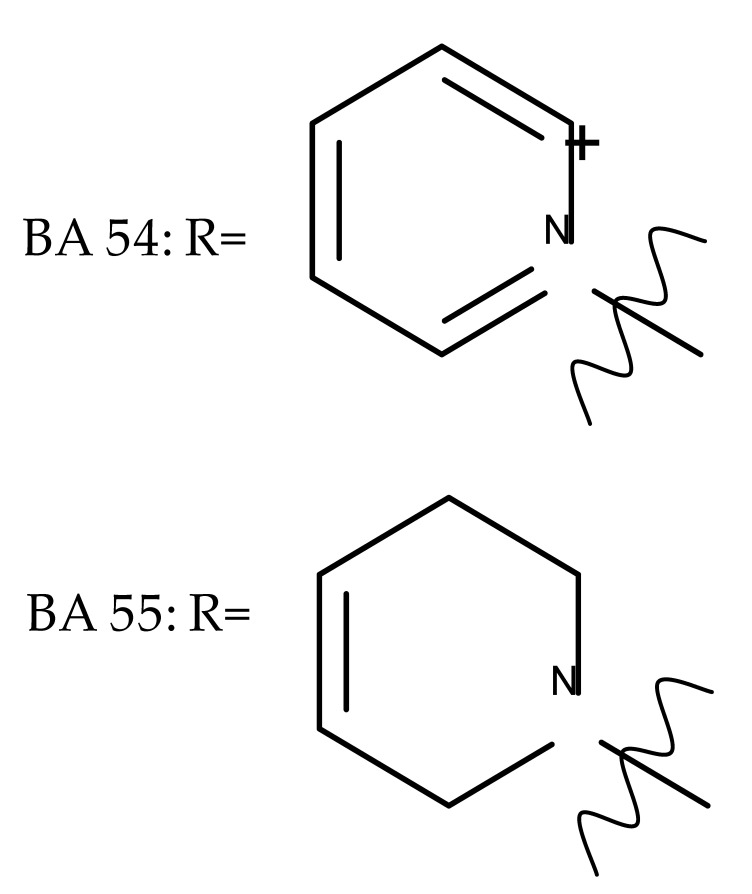	[92]
**BA 56–57**	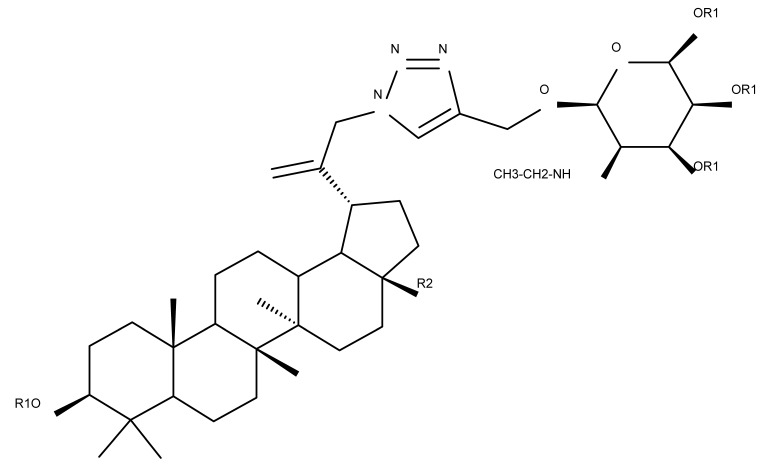	BA 56: R1= CHR-CH2-; R2= -COOH BA 57: R1= H; R2= -CH2-OH	[93]
**BA 58**	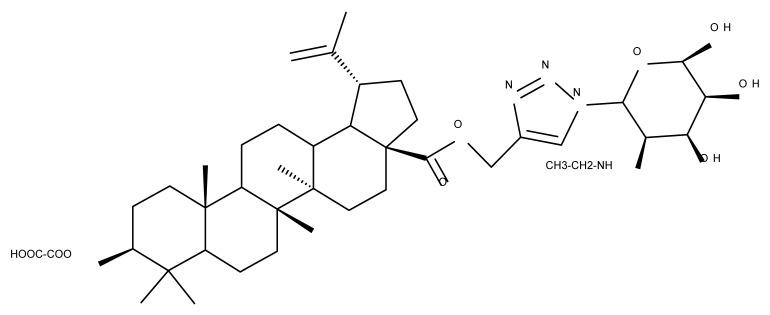	-	[94]
**SH479**	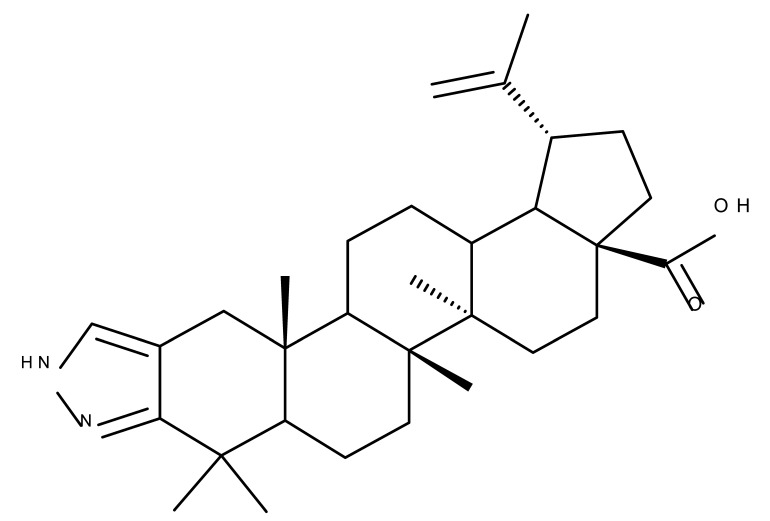	-	[95]

**Table 5:** Chemical structures of BoA derivatives.

**Compound**	**Structure**	**Substituents**	**Reference**
**BoA 1–2**	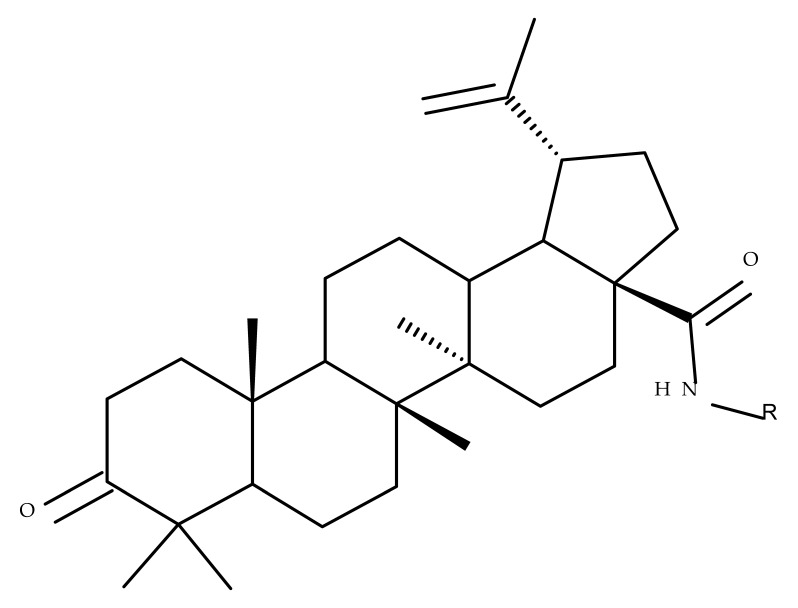	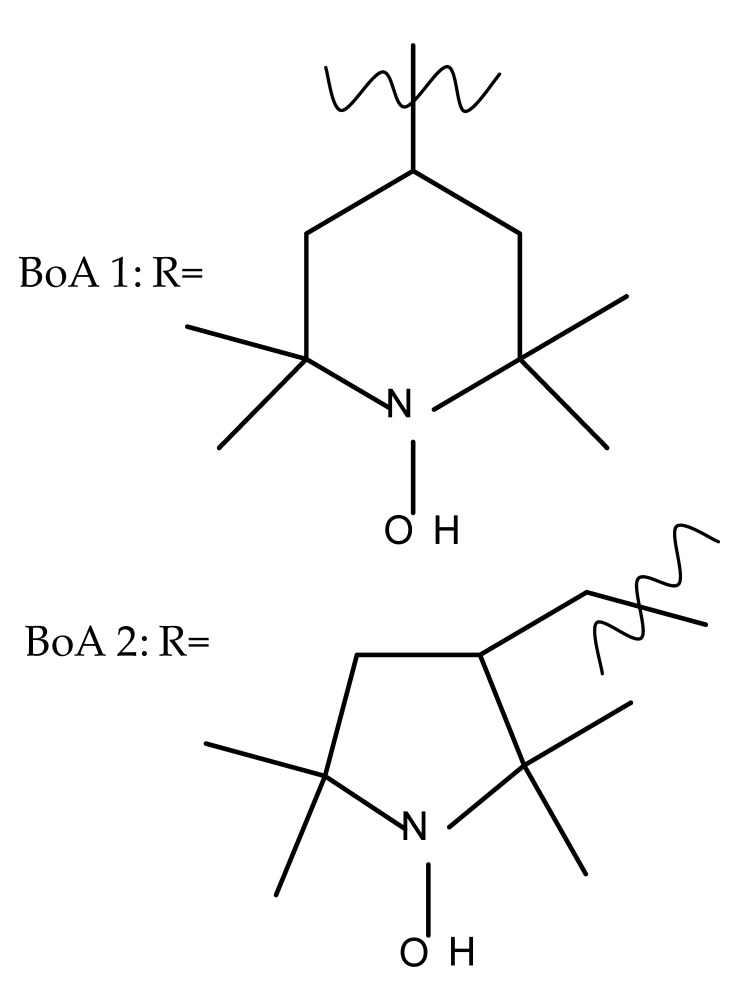	[102]
**BoA 3**	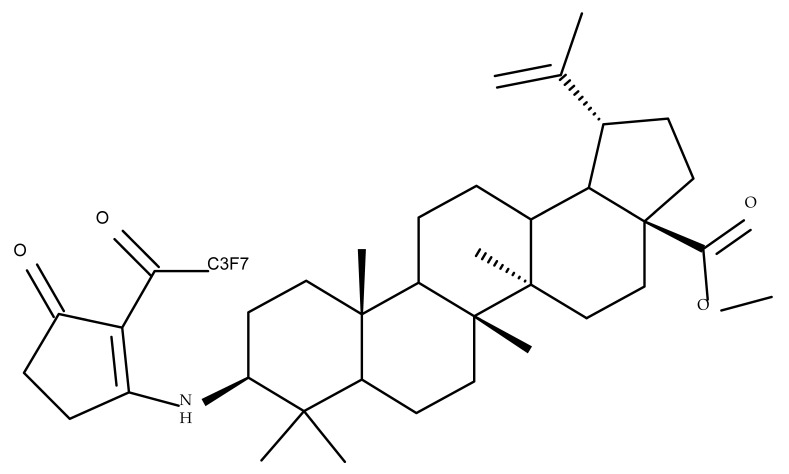	-	[103]
**BoA 4–5**	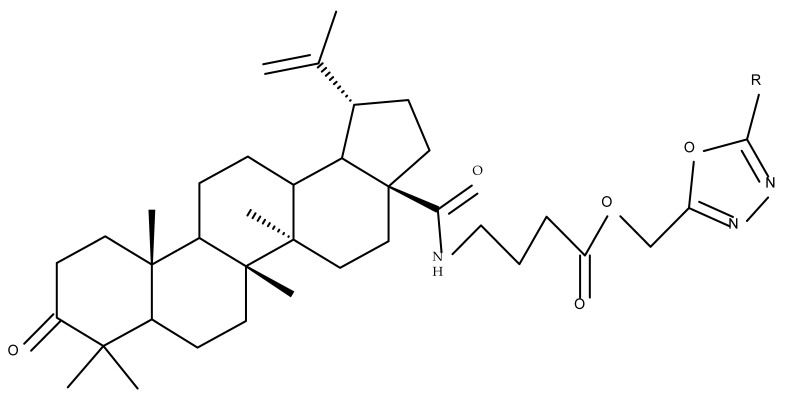	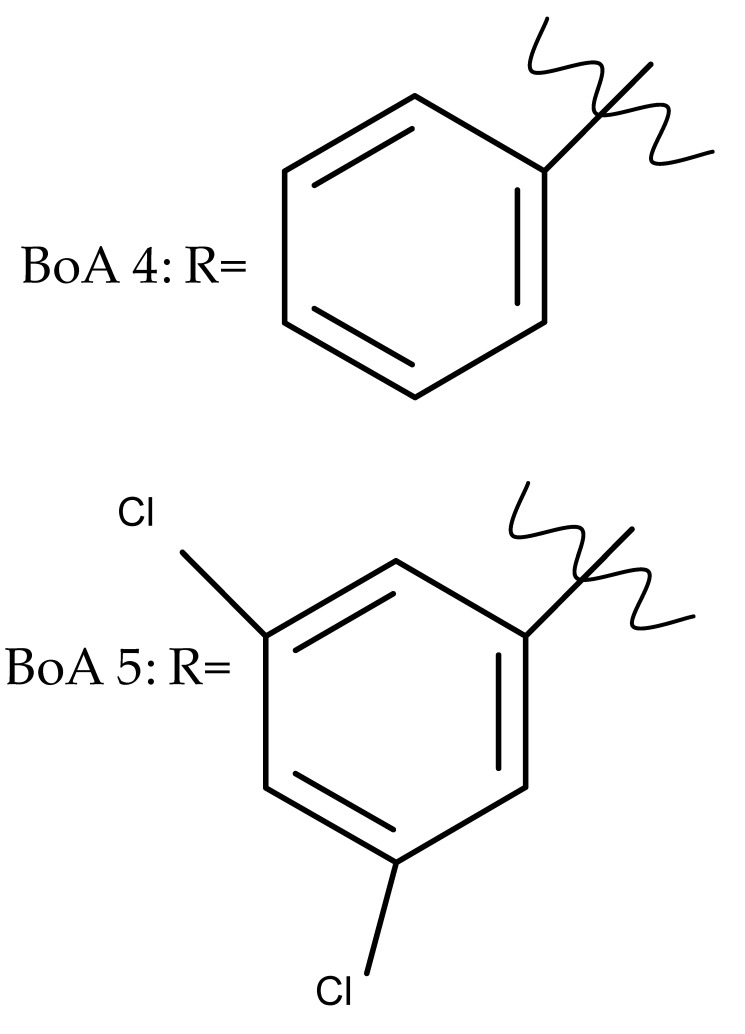	[104]
**BoA 6**	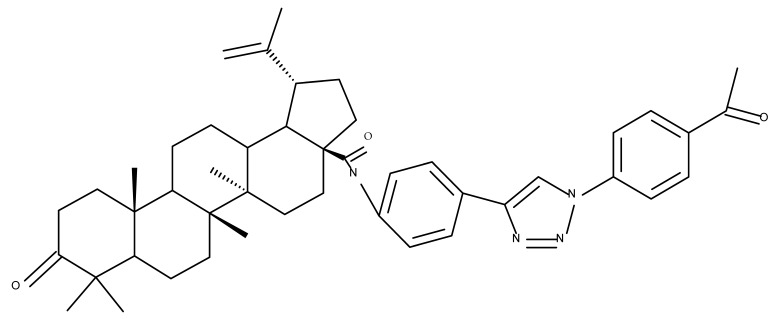	-	[105]
**BoA 7–8**	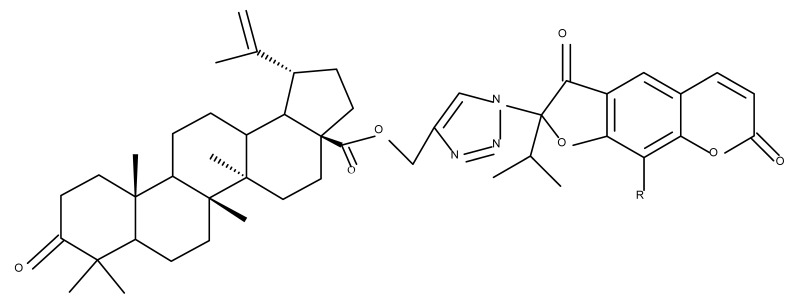	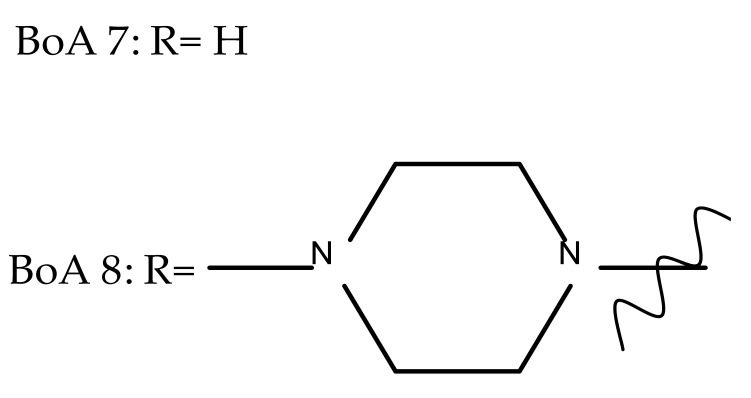	[106]
**BoA 9**	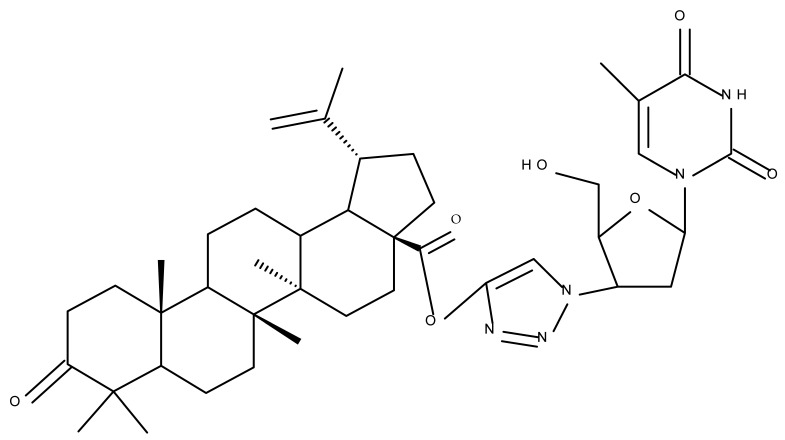	-	[107]
**BoA 10**	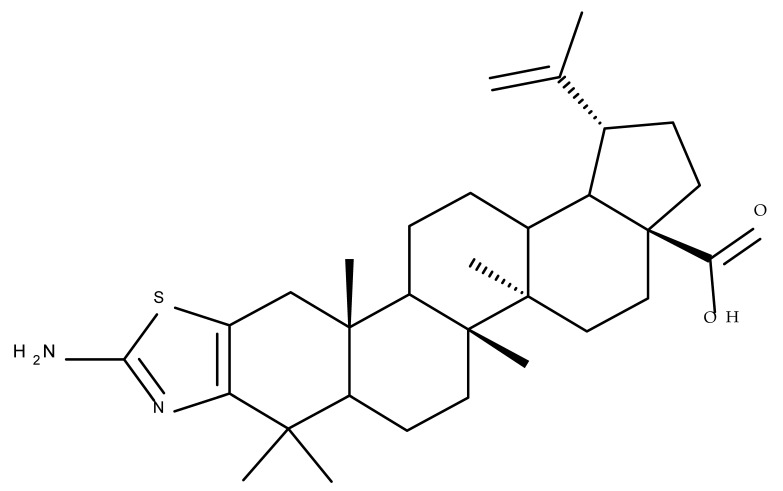	-	[108]
**BoA 11**	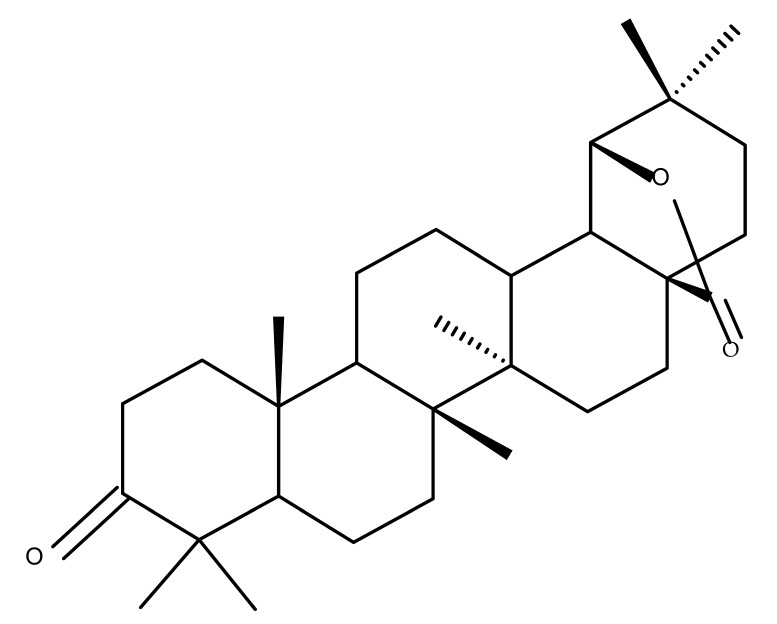		[109]
**BoA 12**	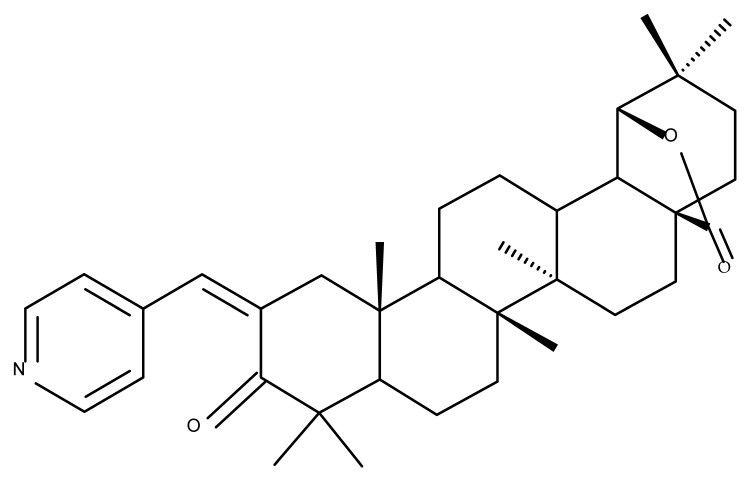		[109]
**BoA 13**	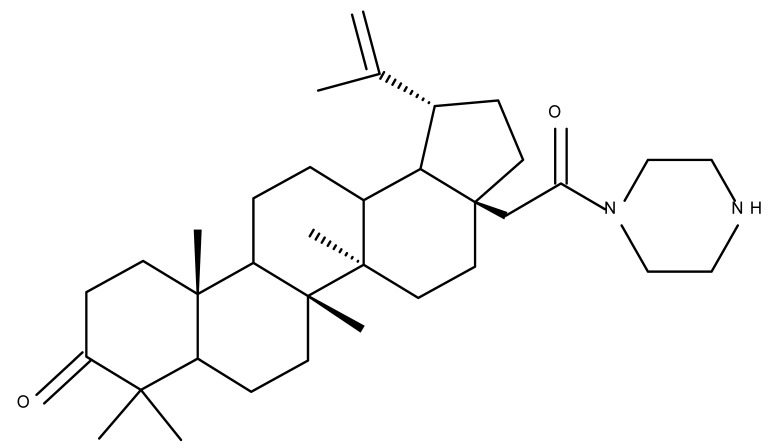	-	[110]
**BoA 14**	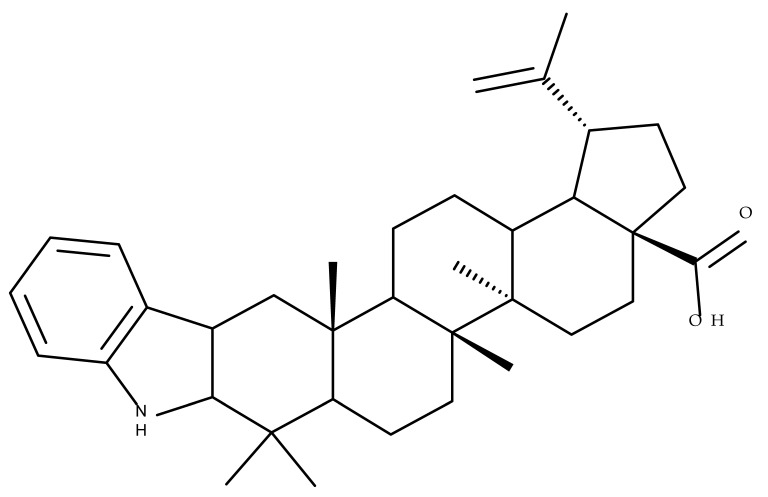	-	[111]

**Table 6:** The chemical structures of ursolic acid derivatives.

Compounds	Structure	Substituents	Reference
**UA 1–4**	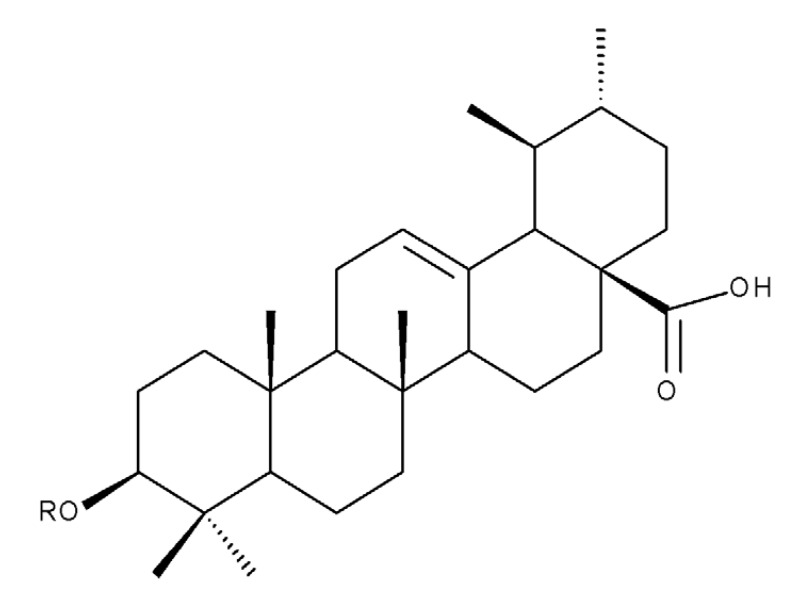	**UA 1** R: -Rha 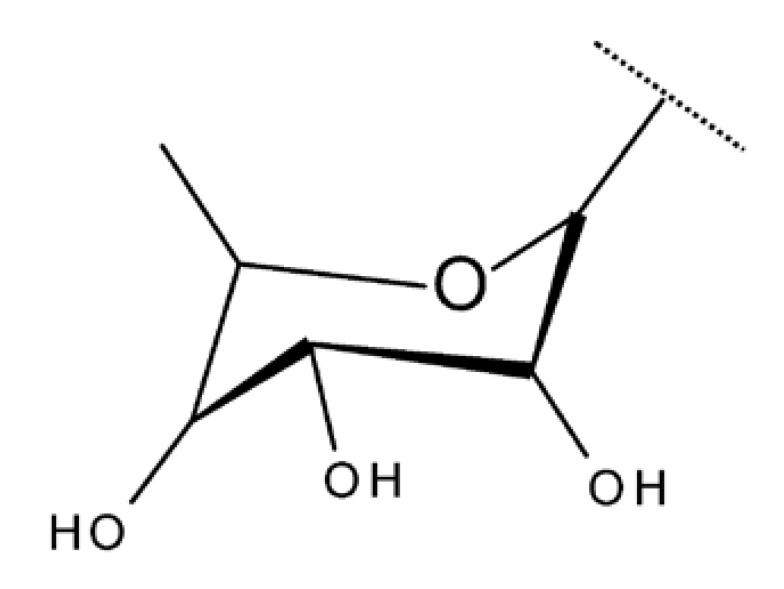 **UA 2** R: -diRha **UA 3** R: -triRha **UA 4** R: -tetraRha	[59]
**UA 5–6**	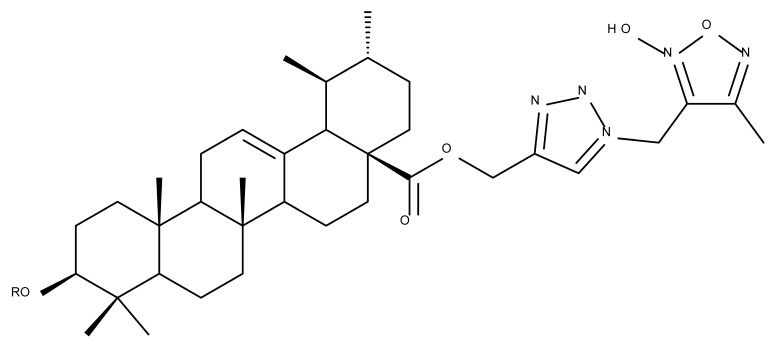	**UA 5** R: -H **UA 6 ** R: -C=O-CH_3_	[114]
**UA 7**	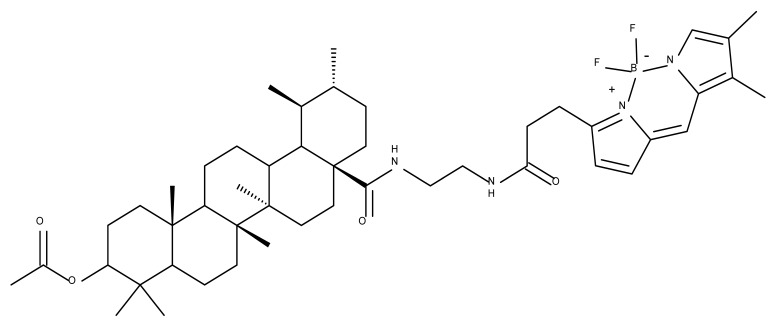	-	[115]

**Table 7:** The chemical structures of oleanolic acid derivatives.

**Number**	**Structure**	**Substituents**	**Reference**
**OA 1**	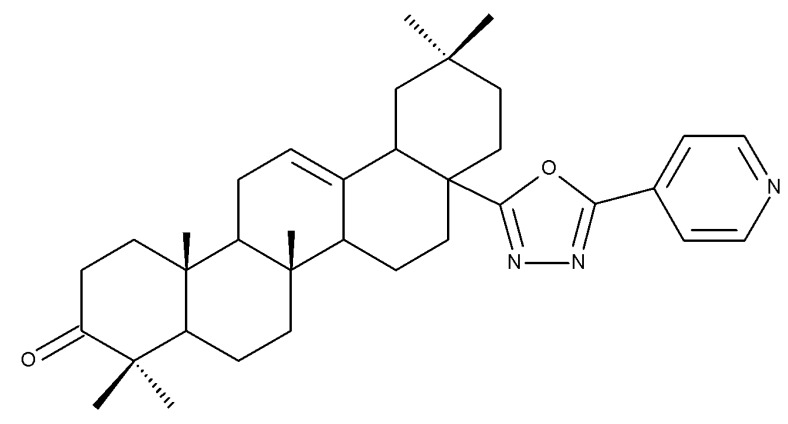		[122]
**OA 2**	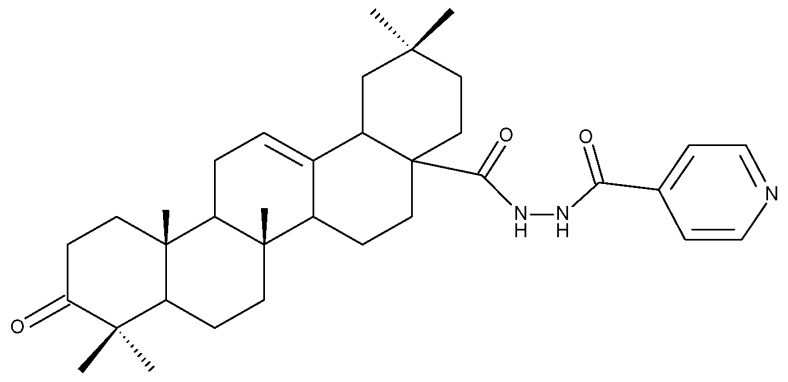		

## Data Availability

Not applicable.
